# Chimeric Protein Complexes in Hybrid Species Generate Novel Phenotypes

**DOI:** 10.1371/journal.pgen.1003836

**Published:** 2013-10-03

**Authors:** Elzbieta M. Piatkowska, Samina Naseeb, David Knight, Daniela Delneri

**Affiliations:** Faculty of Life Sciences, Michael Smith Building, University of Manchester, Manchester, United Kingdom; Aarhus University, Denmark

## Abstract

Hybridization between species is an important mechanism for the origin of novel lineages and adaptation to new environments. Increased allelic variation and modification of the transcriptional network are the two recognized forces currently deemed to be responsible for the phenotypic properties seen in hybrids. However, since the majority of the biological functions in a cell are carried out by protein complexes, inter-specific protein assemblies therefore represent another important source of natural variation upon which evolutionary forces can act. Here we studied the composition of six protein complexes in two different *Saccharomyces* “sensu stricto” hybrids, to understand whether chimeric interactions can be freely formed in the cell in spite of species-specific co-evolutionary forces, and whether the different types of complexes cause a change in hybrid fitness. The protein assemblies were isolated from the hybrids via affinity chromatography and identified via mass spectrometry. We found evidence of spontaneous chimericity for four of the six protein assemblies tested and we showed that different types of complexes can cause a variety of phenotypes in selected environments. In the case of *TRP2/TRP3* complex, the effect of such chimeric formation resulted in the fitness advantage of the hybrid in an environment lacking tryptophan, while only one type of parental combination of the MBF complex allowed the hybrid to grow under respiratory conditions. These phenotypes were dependent on both genetic and environmental backgrounds. This study provides empirical evidence that chimeric protein complexes can freely assemble in cells and reveals a new mechanism to generate phenotypic novelty and plasticity in hybrids to complement the genomic innovation resulting from gene duplication. The ability to exchange orthologous members has also important implications for the adaptation and subsequent genome evolution of the hybrids in terms of pattern of gene loss.

## Author Summary

The *Saccharomyces cerevisiae* “sensu stricto” group represent an excellent example of closely related species which can readily hybridise to occupy new ecological niches. Hybrids harbour the DNA of both parents and can display diverse pattern of gene expression. Less is known about the protein interactions that occur in hybrids, where two diverged proteome co-exist and are responsible for the correct execution of the biological function. In fact, hybrids could potentially form different chimeric variants of the same protein complex by using all the different combinations of parental alleles available. Chimeric interactions are expected to be sub-optimal and therefore discouraged since the members forming the protein complex are from different parents and have a different evolutionary history. Interestingly, here, we show experimentally that chimeric protein assemblies are spontaneously established in different yeast hybrids, and that such chimericity produces different phenotypic variants displaying loss or gain of fitness according to their genetic background and to the environment that they are exposed. These findings imply that the formation of chimeric complexes offers a new source of natural variation, widens the adaptation potential of the hybrids towards new nutritional contexts, and may influence genome evolution through selective retention of optimal alleles.

## Introduction

The *Saccharomyces sensu stricto* yeasts represent a diverse, monophyletic group of species that have the ability to produce viable and stable hybrids that can propagate mitotically. Hybrids among yeast species and strains seem to be common, especially amongst wine, and beer brewing yeasts [1], [2], but also within natural ecological niches [3]. When two parental genomes merge in yeast hybrids there is a potential for genetic novelty but also for a genetic conflict to occur. Dominant genetic incompatibilities do not seem to occur in the *S. cerevisiae sensu stricto* group [4], however evidence of recessive allelic incompatibilities between nuclear and mitochondrial genomes have recently been uncovered [5].

Hybridization can play an important role in evolution since hybrids could occupy a different niche from both parental species and eventually establish a new lineage. The presence of naturally occurring yeast hybrids isolated from specific environments seem to confirms this hypothesis [6], [7]. So far, many unique characteristics of the *Saccharomyces* “sensu stricto” species and hybrids have been attributed to changes in gene expression, including novel *cis*-*trans* interactions [8] and to divergence in regulatory regions [9]. Nevertheless, in the hybrid cellular environment, where two sets of homologous proteomes coexist, there is also the potential for the cell to form chimeric assemblies between homologus protein complexes. Analysis of large-scale proteomics data has shown that the majority of cellular processes are carried out by protein assemblies rather than single proteins and that over 60% of yeast proteins form obligate complexes [10]. Since the correct formation of a complex is essential to carry out the biological function, we would expect that any sub-optimal protein interaction would be detrimental to the cell and therefore discouraged by the cell. On the other hand, spontaneous chimeric assemblies may widen the adaptation potential of the cell, since several different combinations of the same protein complex can be used. Therefore, such situation can lead to new phenotypic variants that are beneficial to the hybrid in novel contexts. The primary aim of this work is to establish proof of principle that chimeric protein complexes can form freely in hybrids of *Saccharomyces* species despite the intra-specific co-evolutionary forces and to quantify the impact that such complexes can have on the overall fitness of the hybrids. In fact, chimericity in protein-protein interaction represents a potentially important mechanism for generating phenotypic diversity upon which evolutionary forces can act, and may constitute a molecular explanation of hybrid vigour.

## Results and Discussion

### Experimental strategy for the analysis of chimeric complexes in yeast hybrids

To test for the existence of natural chimeric complexes in yeast hybrids, we analysed six physically stable ‘obligatory’ protein complexes (Table S1) each of which have constitutively expressed members that were previously recovered by large-scale protein interaction studies and also by independent small-scale biochemical studies [11], [12].

**Figure 1:**
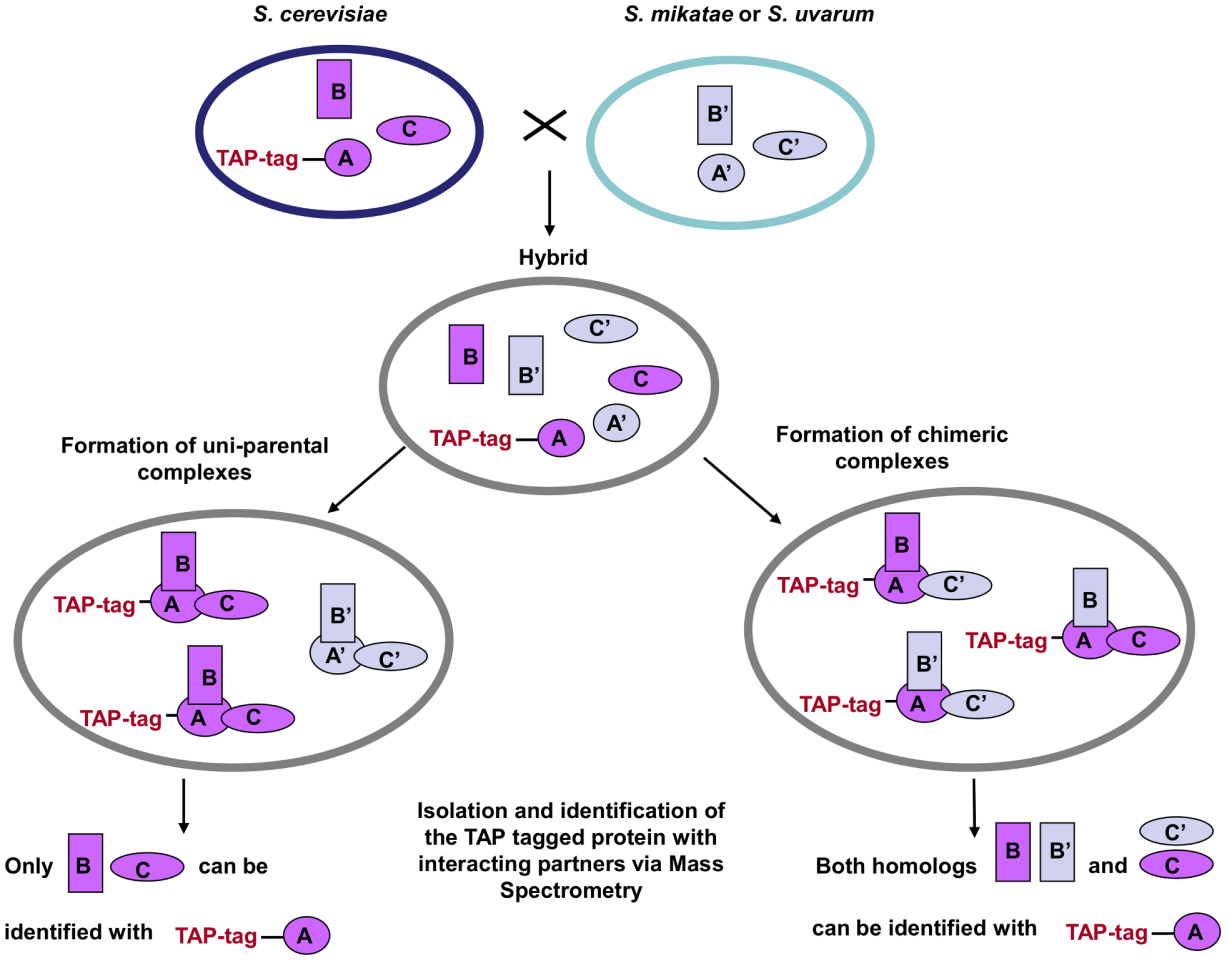
TAP-strategy for recovery and identification of hybrid protein complexes. *S. cerevisiae* strains with the TAP cassette inserted into the C-terminal of one member of the complex (TAP-tag A) were crossed with *S. mikatae* and *S. uvarum* species. The complexes that freely formed in the hybrids were then isolated and the interacting members identified via MS analysis. A', B' and C' represent the orthologs of the *S. cerevisiae* A, B, C proteins, respectively.

We created *S cerevisiae/S. mikatae* (*Sc/Sm*) and *S. cerevisiae/S. uvarum* (*Sc/Su*) hybrids by crossing either *S. mikatae* or *S. uvarum* with *S. cerevisiae* strains carrying a molecular tag (TAP-tag) at the C-terminus of a selected member of the protein complex (Figure S1). Tagged proteins, along with their interacting partners, were isolated via affinity chromatography and all the members of the protein complex were identified via mass spectrometry. If only species-specific parental complexes are established in the hybrid, just proteins from the species carrying the TAP-tag (*S. cerevisiae*) will be identified. However, if chimeric protein complexes are formed, proteins from the other parental species (*S. mikatae* or *S. uvarum*) will also be isolated and identified (Figure 1). The protein fractions were analyzed by mass spectrometry to identify tryptic peptides in a custom protein database of six *Saccharomyces sensu stricto* yeast proteomes. Species-specific peptides were distinguished from the shared peptides that are identical between the two parental species. As control experiment to test whether *in vitro* chimeric interactions were generated artefactually during the protein extraction procedure (as opposed to *in vivo* within the hybrid cellular environment), a mixture of parental cells (i.e. *S. cerevisiae* and *S. mikatae* or *S. uvarum*) were grown separately and mixed together just prior to cell lysis. To establish that both parental genomes were present, all hybrids were screened for chromosomal content via PCR using species-specific primers (Figure S2). To check for genomic alterations after hybridisation, meiosis was induced and spore viability was assessed. Hybrids between yeast species are sterile (<1% survival rate) but they can present a higher rate of spore viability if the cells undergo aneuploidy incrementing their chromosomes number. After dissecting 128 tetrads per hybrid background, no viable cells were detected (Figure S3), suggesting that the hybrids were 2n.

Transcription of the homologous members of the protein complexes in the hybrids was also confirmed via RT-PCR (Figures S4, S5, S6, S7, S8, S9).

### Analysis of the nature of the protein complexes in yeast hybrids

**Figure 2:**
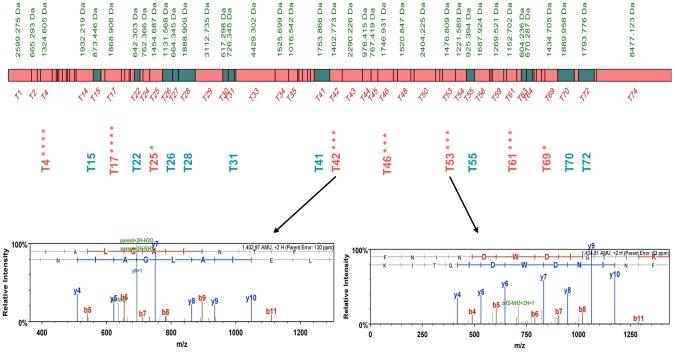
Peptide map of the *S. uvarum* Sec63p. The peptides in common for both *S. cerevisiae* and *S. uvarum* species are shown as green boxes, while *S. uvarum* specific peptides are shown as pink boxes. Unique peptides detected independently in different biological repeats are marked with asterisks. The MS spectra of unique *S. uvarum* ion peptides T42 and T53 are shown below.

The first complex we considered was the Sec 62/63 complex, a tetramer that is involved in the transport of proteins across the ER membrane, composed of two essential proteins, Sec62p and Sec63p and two non-essential proteins, Sec66p and Sec72p [13]. In both hybrids *Sc/Sm* and *Sc/Su*, the mass spectrometry analysis identified Sec63p and Sec72p from either *S. mikatae* or *S. uvarum*, respectively, demonstrating that in yeast hybrids the assembly of the Sec62/63 complex can be spontaneously chimeric (Figure 2, Figure S10, S11, S12, S13, S14, S15, S16, S17, Table S2 and S3).

Evidence of chimeric interactions were also detected between members of the *TRP2/TRP3* complex, involved in the tryptophan biosynthesis [14] (Figures S18 and S19, Tables S4 and S5) and the CTK complex, involved in transcription and translation regulation [15] (Ctk1p, Ctk2p, Ctk3p; see Figures S20 and S21, Table S6 and S7), in both *Sc/Sm* and *Sc/Su* hybrids.

In the case of the MBF complex, a dimer composed of two proteins, Mbp1 (a transcription factor responsible for DNA synthesis at the G1/S phase of the cell cycle) and Swi6p (a *trans*-activating component) [16], chimeric complexes were only identified in hybrids *Sc/Su*, while, surprisingly, no free interaction was detected in the hybrids of the more closely related species *S. cerevisiae* and *S. mikatae* (Figures S22 and S23, Tables S8 and S9). Targeted mass-spectrometry was also performed on *Sc/Sm* hybrid to seek specifically *S. mikatae* Swi6 peptides, which constituted the majority of the tryptic digest (ca 76% of all peptides). However, no specific *Sm* peptides were detected, indicating that this protein was not present in the complex at significant levels (Table S10). The level of expression of *Sm* Swi6p is higher than that that one of *Sc* Swi6p in *Sc/Sm* background, and is also higher than that one of *Su* Swi6p in *Sc/Su* hybrids, as showed by Real time PCR experiments (Figure S24), ruling out the lack of detection due to the insufficient expression of Swi6p in the *Sc/Sm* hybrid. This results indicates that, given the choice, Mbp1p from *Sc* prefer to form uni-specific complexes with Swi6p from *Sc* in *Sc/Sm* background. When considering protein-protein interactions the sequence identity of the binding interfaces is likely to be more important than the phylogenetic relationship. In fact, Swi6p shows greater gene sequence similarity between *S. cerevisiae* and *S. uvarum* than between *S. cerevisiae* and *S. mikatae*, despite their phylogeny (Figure S25).

The remaining two complexes tested, the RAM (Ram1p and Ram2p, farnesyltransferase complex involved in the prenylation of Ras proteins) [17] and KU (Yku70p and Yku80p), involved in double strand breaks repair and non-homologous end joining) [18], appeared unable to form chimeric complexes in any hybrid background (Tables S11, S12, S13, S14). In fact, using Yku70p as TAP-bait, no specific Yku80p peptides from *S. uvarum* and *S. mikatae* parental species were ever found in any biological replica tested, while numerous *S. cerevisiae* specific Yku80p peptides were consistently isolated. Although the failure to detect such interactions in mass spectrometry is not a definite proof that chimeric complexes are not at all assembled, this data suggests that chimericity within RAM and Ku complexes may at least occur rarely, and that the proteins forming such complexes tend to assemble in uni-specific manner if given the option. Interestingly, an independent study of the KU complex in hybrids of two diverged strains of *S. paradoxus* showed that negative epistatic interactions occur between the different homologues of Yku70p and Yku80p, suggesting either lack of assembly or functionality of the heterodimer [19]. The inability to detect spontaneous chimeric complex formation in both *Sc/Sm* and *Sc/Su* hybrids observed in this work support the idea that the prevention of complex formation could be the possible mechanism for the negative epistasis identified between Yku70p and Yku80p in the *S. paradoxus* strains.

### Phenotypic variations caused by different types of protein assemblies

We evaluated the impact that chimeric interactions have on fitness by forcing the hybrids to use only one specific type of complex to carry out the biological function. We chose to investigate the *TRP2/TRP3* ad the MBF complex, since the relationship between the functional complexes and the resulting output fitness could be clearly measured under tryptophan starvation and respiratory growth condition, respectively. In fact, the *TRP2/TRP3* complex is involved in the first step of the tryptophan biosynthesis [14], and null mutants of Mbp1p and Swi6p display a range of fitness defects including decrease rate of respiratory growth and abnormal mitochondrial morphology [20].

**Figure 3:**
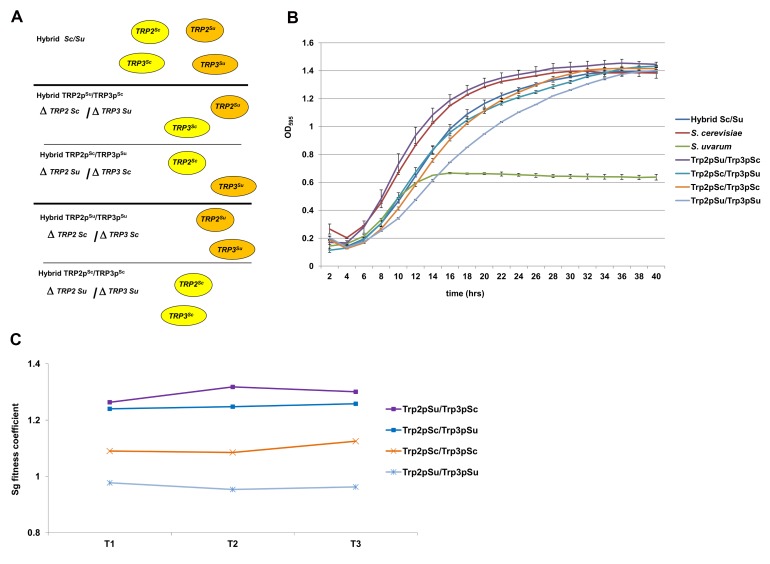
Fitness assays of the engineered *Sc/Su* hybrids carrying different type of *TRP2/TRP3* chimeric complexes. *Sc/Su* hybrids were genetically modified to carry either the two different chimeric complexes, Trp2p^Su^/Trp3p^Sc^ and Trp2p^Sc^/Trp3p^Su^, or the two parental hemizygous controls, Trp2p^Su^/Trp3p^Su^ and Trp2p^Sc^/Trp3p^Sc^ (panel A). The growth curves of *S. cerevisiae*, *S. uvarum*, the hybrid *Sc/Su* and the engineered hybrids shows that Trp2p^Su^/Trp3p^Sc^ grows better than the other combinations in SD media lacking tryptophan (panel B). The fitness competition assay between *Sc/Su* hybrids with different combination of the *TRP2/TRP3* complex and the GFP reference strain shows again that Trp2p^Su^/Trp3p^Sc^ grows faster (panel C). The competitive fitness coefficient Sg represents the difference between the ln of the ratio of hybrid and reference strain between final and initial time points, normalized for the number of generations. An equal fitness between hybrid and reference strains would be indicated by a value of zero (see Method section).

**Figure 4:**
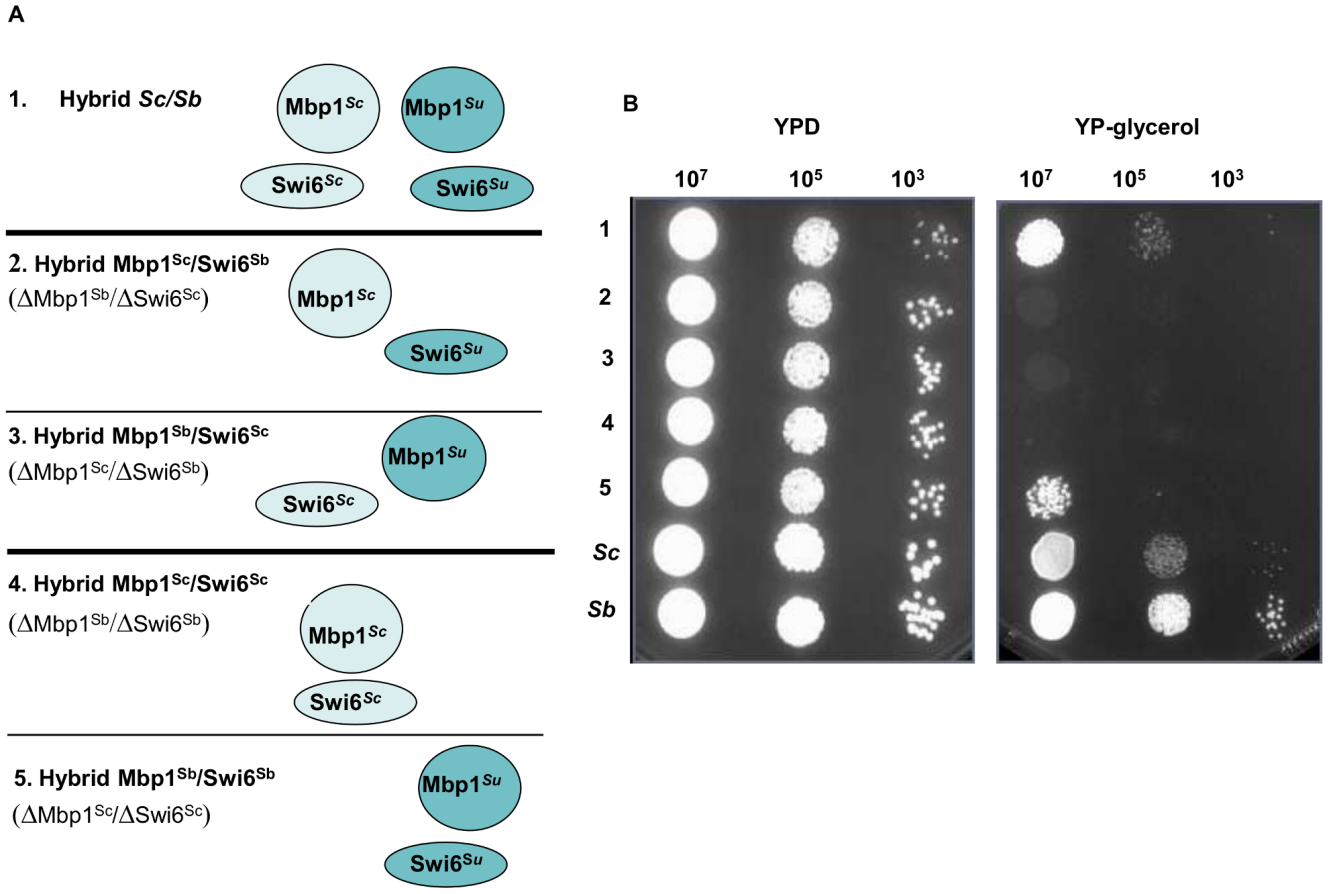
Growth assays of *Sc/Su* hybrids carrying different types of MBF chimeric complexes. *Sc/Su* hybrids were genetically modified either to carry the two different chimeric complexes, Mbp1^Su^/Swi6^Sc^ and Mbp1^Sc^/Swi6^Su^, or the two uni-parental controls, Mbp1^Su^/Swi6^Su^ and Mbp1^Sc^/Swi6^Sc^ (Panel A). The growth spot assay of the engineered hybrids in rich YPD and YP-glycerol media are shown in Panel B. The strain carrying the *S. uvarum* homologous Mbp1^Su^ and Swi6^Su^ is the only one that performs respiratory growth and grows normally in the presence of glycerol a sole carbon source.

We created different combinations of the *TRP2/TRP3* and MBF complexes by deleting different protein members in both *Sc/Sm* and *Sc/Su* hybrid backgrounds (Figures 3A and 4A), and then scored the growth rates of the hybrids carrying either uni-specific or chimeric complexes.

For the *TRP2/TRP3* complex in the *Sc/Su* background, a large range of fitness levels was detected for the different types of assemblies (Figure 3B). The *S. uvarum* parent grows poorly compared to the *S. cerevisiae* parent, while the hybrid shows an intermediate fitness (Figure 3B). When comparing the growth of the four strains bearing different combinations of *TRP2/TRP3* protein complexes (*i.e.* possessing the same *TRP2/TRP3* copy number in the same hybrid genetic background), we found that the strain with the Trp2p^Su^/Trp3p^Sc^ chimeric complex grew much better than all the other strains in a medium lacking tryptophan (Figure 3B). The uni-parental hemizygous controls Trp2p^Su^/Trp3p^Su^ showed the lowest fitness, while the chimeric Trp2p^Sc^/Trp3p^Su^ and the hemizygote Trp2p^Sc^/Trp3p^Sc^ showed an intermediate growth (Figure 3B). When tryptophan was added to the SD medium the phenotypic difference between the hybrids carrying different protein complexes was minimised (Figure S26).

The strain with the chimeric combination Trp2p^Su^/Trp3p^Sc^ seems to grow similarly to the *S. cerevisiae* parent and better than the original hybrid. It is possible that, in the parent hybrid, a higher percentage of uni-specific *S. uvarum* complexes are formed, which are the most unfit of all four combination (Trp2p^Su^/Trp3p^Su^, Figure 3B and C), and could therefore partially compromise the fitness of the hybrid. In fact, although the quantitative expression of the two *TRP2* orthologs is similar in the hybrid, the *S. uvarum TRP3* copy is more expressed than the *S. cerevisiae* counterpart (Figure S27).

To confirm the increased fitness of the strain expressing a Trp2p^Su^/Trp3p^Sc^ chimeric complex, competition experiments between the chimeric hybrids and a GFP reference strain was carried out using FACS analysis [21]. The results showed that strains with the chimeric Trp2p^Su^/Trp3p^Sc^ complex were more fit than those with the other chimeric complex (Trp2p^Sc^/Trp3p^Su^) and those with both uni-specific protein-protein interaction combinations (Figure 3C). Moreover, a competitive growth essay between the hybrid carrying the fittest chimeric complex Trp2p^Su^/Trp3p^Sc^ and the reference strain was carried out in SD medium with and without tryptophan. The fitness gain of the strain carrying Trp2p^Su^/Trp3p^Sc^ complex was lessened in the medium containing tryptophan (Figure S28).

For the MBF complex in the *Sc/Su* background all the engineered hybrids carrying the different type of complexes were able to grow on glucose medium, however only the hybrid carrying the uni-specific combination Mbp1p^Su^ and Swi6p^Su^ derived from *S. uvarum* was able to grow in media containing glycerol, a carbon source that can only be respired (Figure 4). The other parental combination of Mbp1p^Sc^/Swi6p^Sc^ could not be rescued by adding either Mbp1p^Su^ or Swi6p^Su^ to its genotype, showing that the presence of both *S. uvarum* members of the MBF complex is required for hybrid growth on glycerol (Figure S29). Interestingly, the restriction analysis of the mitochondrial genes *COX2* and *COX3* indicated that the *Sc/Su* hybrids harbour the *Su* mitochondrial DNA (data not shown). Recently, incompatibilities between nuclear and mitochondrial genes have been proposed as general mechanism causing reproductive isolation between species.

This is a type of Dobzhansky-Muller incompatibility involving lack of interaction or malfunctioning of interacting alleles derived from two different species. For example, the *S. uvarum* nuclear encoded mitochondrial protein Aep2p is unable to regulate the translation of the *S. cerevisiae* mitochondrial gene OLI [5], and the *S. cerevisiae* Mrs1p is not able to splice either the *S. paradoxus* or the *S. uvarum COX1* gene [22].

In the case of MBF complex, we have shown an example of phenotypic plasticity of different chimeric assemblies, and found a novel case of hybrid incompatibility between *S. cerevisiae* and *S. uvarum* when cells are grown on a non–fermentable medium and the mitochondria function become essential for cell viability.

Fitness variation between the different types of protein assemblies was not otherwise observed in *Sc/Sm* hybrids either for the *TRP2/TRP3* or for the MBF complex (Figure S30), underlying the dependency of these phenotypes on their genetic background (manifesting in *Sc/Su* but not in *Sc/Sm* hybrids). This background dependency is not entirely surprising given the fact that, even between two strains belonging to the same *S. cerevisiae* species (*i.e.* BY4743 and Sigma 1278b) several conditional essential genes have been discovered [23].

### Conclusions

Here we have shown that protein complexes in hybrids of *S. cerevisiae/S. mikatae* and *S. cerevisiae/S. uvarum* are able to spontaneously exchange components for inter-specific orthologs, and, while this manuscript was under review, a study on protein-protein interactions among members of the nuclear pore complex and the RNA polymerase II complex in other *S. cerevisiae* “sensu stricto” hybrids (i.e. *S. cerevisiae/S. kudriazvevii*) also concluded that chimeric protein complexes could assemble [24].

Out of the six complexes studied four were convincingly found to form natural chimeric protein assemblies in either one or both genetic hybrid background (i.e. Sec62–63, *TRP2/TRP3*, *MBF*, and CTK complex). These results provide evidence that chimeric protein interactions in hybrids can arise to generate evolutionary novelty in protein-protein interaction networks, providing a new evolutionary mechanism to complement innovation by gene duplication [25].

We also found that some complexes prefer to form species-specific configurations in the natural hybrid cell environment (i.e. Ku and RAM complex). The lack of spontaneous chimeric assembly in these cases could be due to less favourable changes in the binding interfaces of the proteins, or to stoichiometry imbalance between homologous proteins in the hybrid [26]. The inability to create chimeric interaction can be responsible for some negative epistatic effect seen in hybrids [19].

We showed that different type of complexes can cause a variety of phenotypes in selected environments. In the case of *TRP2/TRP3*, we find that chimeric complex formation can lead to hybrid vigour, reinforcing the idea that the ability to form different types of protein assemblies could be advantageous to the hybrid in specific nutritional contexts. We can speculate that the advantage of the chimeric combination can be due to a more harmonious expression of some alleles leading to a better stoichiometry of that specific type of complex. Alternatively, the chimeric complex may be more efficient in its biological function in the hybrid background.

In the case of MBF complex only one parental combination of protein-protein interaction was compatible with cell viability under respiratory condition, highlighting a new case of allelic incompatibilities in yeast hybrids. These phenotypes were proved to be dependent on both genetic and environmental backgrounds since we did not observe any fitness change in *Sc/Sm* hybrids and the advantages could be lost or gained in different media, such as in the case of the strains carrying different combination of the MBF complex grown in YPD or YP-glycerol (Figure 4B).

Ultimately, this study proposes a novel molecular mechanism for creating phenotypic variation within a hybrid cell, with important implications for understanding the evolutionary forces that govern the reshaping of hybrid genomes. The genomic fate of the homolog genes will in fact be influenced by the ability or not of the hybrid to create inter-specific protein assemblies (Figure S31). Moreover, chimeric complexes may be able to recruit new proteins and evolve new functions in the cell [27]. In the future, the genomic information of naturally occurring hybrids (like *S. pastorianus* strains) will provide insight into the nature of how the formation of chimeric interactions influences selective gene retention of members of protein complexes and networks.

## Materials and Methods

### Generation of yeast hybrids

All the TAP-tagged constructs, based on *S. cerevisiae* MGD353-13D strain, were obtained from the EUROSCARF strains collection (http://web.uni-frankfurt.de/fb15/mikro/euroscarf/cellzome.html). Hybrids between *S. cerevisiae* strains (bearing the TAP-tag in selected members of different protein complexes) and wild-type *S. mikatae* 1815 and *S. uvarum* NCYC2669 species were generated using a Singer Instruments MSM micromanipulator as previously described [28]. To enable selection of hybrid colonies, we made the *S. cerevisiae* TAP strains geneticin-resistant by inserting a kanMX in the neutral *AAD3* locus. Hybrid colonies were then selected on minimal media containing geneticin G418 (see Figure S1). The nature of the chromosomes were verified by chromosomal PCR using genomic DNA from the hybrid as template and species-specific primers designed to distinguish between *S. cerevisiae*, *S. mikatae* and *S. uvarum* alleles (see Figure S2 and Table S15, S16, S17).

After the hybrid was created it took ca. 24 generations (growing in two different selective plates) to select the hybrids before the PCR was made to check the chromosomes, and another 16 generations before the TAP tagging experiment (total of about 40 generations since the production of the hybrid). The hybrid was then maintained in glycerol stock at −80 C.

Hybrid genomic DNA and RNA was isolated using the DNasy Blood & Tissue kit and the RNeasy mini kit (Qiagen, Crawley, UK), respectively.

### Expression analysis by real-time quantitative PCR

The expression levels of *S. cerevisiae*, *S. uvarum* and *S. mikatae SWI6*, *TRP2* and *TRP3* alleles in *Sc/Su* and *Sc/Sm* background were performed on the cDNA samples amplified using the Quantitect real time PCR kit from Qiagen. Optimized reactions were carried out using 10 ng/µl of cDNA, 5 pmoles of each primer and syber green according to the manufacturer instructions (Table S18). Actin (*ACT1*) was used as a housekeeping reference gene. The expression of each gene was estimated using the Ct Values.

### Purification of protein complexes from yeast hybrids and mass spectrometry analysis

Purification of the protein complexes was carried out using the standard TAP protocol [29] optimized for these specific classes of proteins. In particular, two affinity binding steps, the IgG Sepharose and Calmodulin Binding Protein (CBP) binding and TEV protease cleavage were carried out for 2 hours at 4°C instead of 16°C. The protein mixtures were resolved using 1D gel electrophoresis, stained with Coomassie Bio Safe (Bio-Rad) and digested with trypsin (Promega). The trypsin digest was carried out overnight at 37°C according to Shevchenko, A. *et al.* [30]. The digested protein mixture was separated by the high performance liquid chromatography (HPLC) and analyzed by tandem mass spectrometry (ESI MS/MS) (Micromass CapLC-Q-ToF, Waters, Manchester, UK). The system was either used in a discovery manner with the system selecting peptides automatically or in a targeted manner with the system selecting peptides directed from a list of peptides of interest. Spectra acquired for every protein complex member were compared against a custom database containing all proteins from *S. cerevisiae* “sensu stricto” species, using Mascot version 2.2.06 (Matrix Science Inc., Boston, MA). Scaffold (Scaffold_2_01_00, Proteome Software Inc., Portland, OR) was used to validate MS/MS based peptide identification. A peptide match was acknowledged if it could be established at greater than 50.0% probability as specified by the Peptide Prophet algorithm [31]. The peptide criteria were set to 50% as we were looking specifically at homologous proteins and shared peptides are generally given lower confidence scores because it cannot be determined which protein the peptides originate from. Significant peptides were checked manually to ensure all the major fragments were matched and a contiguous series of at least 4 y or b ions were present. Protein identifications were accepted if they could be established at greater than 95.0% probability by Protein Prophet and contained at least 2 identified peptides. The Liverpool Peptide Mapping Tool (http://www.liv.ac.uk/pfg/Tools/Pmap/pmap.html) was used to generate proteolytic peptide maps of protein complex members. The peptide maps were generated with one trypsin miscleavage per site after lysine and arginine (K-X, R-X) but not at lysine-proline and arginine-proline (K-P, R-P) sites.

### Generation of chimeric protein complexes in *Sc/Sm* and *Sc/Su* hybrids and fitness assays

Chimeric and unispecific versions of the *TRP2/TRP3* and MBF complexes in both *Sc/Sm* and *Sc/Su* hybrids were generated by PCR-mediated gene deletion strategy using hygromycin (*HPH*) and nourseothricin (NAT) as selectable markers [32]. The *S. cerevisiae TRP2* and *TRP3* copies were replaced with *HPH* while the *S. uvarum* ones were deleted using *NAT* (see Figure 3). Similarly for the MBF complex, the *S. cerevisiae* orthologs of Mbp1 and Swi6 were disrupted using *HPH*, while the *S. uvarum* copies of Mbp1 and Swi6 were deleted using *NAT* (see Figure 4). Yeast hybrids were grown in YPD, SD and minimal F1 media [33] at 30°C for 40 hours with continuous shaking. Growth rates were measured by absorbance at OD_595_ at 5 minutes intervals using Fluostar Optima bioscreen workstation (BMG Labtech).

Fitness competition assays were carried out by FACS analysis according to Lang *et al.* [21]. As reference strain we used the FY3 strains bearing the GFP tag at the C-terminus of CDC33p (generated for the purpose of this experiment), and the competition was carried out in SD media lacking tryptophan. The hybrids strains were mixed with the reference strain in 4∶1 ratio, and a total of 1×10^5^ cells, counted on a cellometer (Auto M10, Nexcelom), were inoculated into a 1 ml of fresh medium. The strains were allowed to grow for 12 hours and then the ratio of the number of hybrid cells over the fluorescent reference was determined using the Dako CyAn flow cytometer, with a total counting total 50,000 cells for each time point. Three biological and three technical replicates were performed for each fitness measurement. The s_g_ fitness coefficient was calculated using the following equation:
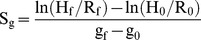
where, H and R are the cell number of the hybrid and reference strain and g_0_ and g_f_ are the number of generations at the beginning and after a time interval (12 hours).

## Supporting Information

Figure S1 Hybrid generation and selection on the selective SD medium with urea and G418. List of hybrid strains generated by crossing *S. cerevisiae* TAP strains with *S. mikatae* and *S. uvarum* (Panel A), and manual crossing of *S. cerevisiae* haploid cells with dissected spores and subsequent selection on SD+G418 (Panel B). Crosses were generated on YPD rich plates and replica plated on selective media. The growth pattern of 2∶2 is expected and was selected for further analysis.
(DOC)

Figure S2 The chromosomal PCR of *Sc/Sm* and *Sc/Su* hybrids. Panel A shows the chromosomal PCR verification of the *Sc/Sm* hybrid. Panel B shows the chromosomal verification of the *Sc/Su* hybrids. The PCR was performed with species- specific primers for both parental species for each chromosome. The lane M is the marker Hyperladder I, and the lanes 1–16 show the PCR products corresponding to the 16 chromosomes from *S. cerevisiae* (higher bands) and *S. mikatae* or *S. uvarum* (lower bands).
(DOC)

Figure S3 The dissection plate of *Sc*/*Sm 1815* hybrid species. Hybrid *Sc/Sm* spore dissection plate after 5 days. No viable spores were detected after dissecting 128 tetrads. Similar results were obtained for *Sc/Su* hybrids (data not shown).
(DOC)

Figure S4 RT-PCR of members of the MBF complex. Panel A shows the amplification of the *MBP1* and *SWI6* cDNA fragments specific to *S. cerevisiae* and *S. mikatae* carried out in the two parental strains and in the hybrid background *Sc/Sm*. Panel B shows the amplification of the *MBP1* and *SWI6* cDNA fragments specific to *S. cerevisiae* and *S. uvarum* carried out in both parental strains and in the hybrid background *Sc/Su*. Panel C shows the control for potential cross-hybridization of the species-specific primers. The RT-PCR using the *S. cerevisiae* MBF specific primers was carried out in either *S. mikatae* or *S. uvarum* background (and vice-versa). No cross-hybridization was detected.
(DOC)

Figure S5 RT-PCR of members of the *TRP2/TRP3* complex. Panel A shows the amplification of the *TRP2* and *TRP3* cDNA fragments specific to *S. cerevisiae* and *S. mikatae* and *S. uvarum* carried out in the parental strains and in the hybrid background *Sc/Sm* and *Sc/Su*. Panel B shows the control for potential cross-hybridization of the species-specific primers. The RT-PCR using the *S. cerevisiae* MBF specific primers was carried out in either *S. mikatae* or *S. uvarum* background (and vice-versa). No cross-hybridization was detected.
(DOC)

Figure S6 RT-PCR of members of the KU complex. Panel A shows the amplification of the *KU70* and *KU80* cDNA fragments specific to *S. cerevisiae* and *S. mikatae* and *S. uvarum* carried out in the parental strains. Panel B shows the amplification of the *Ku70* and *KU80* cDNA fragments specific to *S. cerevisiae S. mikatae* and *S. uvarum* carried out in both hybrid backgrounds *Sc/Sm* and *Sc/Su*. Panel C shows the control for potential cross-hybridization of the species-specific primers. The RT-PCR using the *S. cerevisiae* KU specific primers was carried out in either *S. mikatae* or *S. uvarum* background (and vice-versa). No cross-hybridization was detected.
(DOC)

Figure S7 RT-PCR of members of the SEC62–63 complex. Panel A shows the amplification of the *SEC62*,*SEC63*, *SEC66* and *SEC72* cDNA fragments specific to *S. cerevisiae*, *S. mikatae* and *S. uvarum* carried out in the parental strains. Panel B shows the amplification of the *SEC*62–63 cDNA fragments specific to *S. cerevisiae*, *S. mikatae* and *S. uvarum* carried out in both hybrid backgrounds *Sc/Sm* and *Sc/Su*. Panel C shows the control for potential cross-hybridization of the species-specific primers. The RT-PCR using the *S. cerevisiae SEC*62/63 specific primers was carried out in either *S. mikatae* or *S. uvarum* background (and vice-versa). No cross-hybridization was detected.
(DOC)

Figure S8 RT-PCR of members of the CTK complex. Panel A shows the amplification of the *CTK1*, *CTK2* and *CTK3* cDNA fragments specific to *S. cerevisiae*, *S. mikatae* and *S. uvarum* carried out in the parental strains. Panel B shows the amplification of the *CTK* cDNA fragments specific to *S. cerevisiae*, *S. mikatae* and *S. uvarum* carried out in both hybrid backgrounds *Sc/Sm* and *Sc/Su*. Panel C shows the control for potential cross-hybridization of the species-specific primers. The RT-PCR using the *S. cerevisiae CTK* specific primers was carried out in either *S. mikatae* or *S. uvarum* background (and vice-versa). No cross-hybridization was detected.
(DOC)

Figure S9 RT-PCR of members of the RAM complex. Panel A shows the amplification of the *RAM1* and *RAM2* cDNA fragments specific to *S. cerevisiae* and *S. mikatae* carried out in the parental strains and in the *Sc/Sm* hybrid. Panel B shows the amplification of the RAM cDNA fragments specific to *S. cerevisiae* and *S. uvarum* carried out in the parental strains and in *Sc/Su* hybrid. Panel C shows the control for potential cross-hybridization of the species-specific primers. The RT-PCR using the *S. cerevisiae RAM* specific primers was carried out in either *S. mikatae* or *S. uvarum* background (and vice-versa). No cross-hybridization was detected.
(DOC)

Figure S10 Product ion spectra of *S. cerevisiae* specific peptides characteristic for the Sec62p detected in *Sc/Sm* hybrid. Panel A shows the product spectrum of the 884.47 Da peptide. The sequence of the peptide is AQMVIPK. Panel B shows the product spectrum of the 1385.81 Da peptide. The sequence of the peptide is QPEIYPTIPSNK.
(DOC)

Figure S11 Product ion spectra of *S. cerevisiae* specific peptides characteristic for the Sec62p detected in *Sc/Su* hybrid. Panel A shows the product spectrum of 884.47 Da peptide. The sequence of the peptide is AQMVIPK. Panel B shows the product spectrum of 1385.81 Da peptide. The sequence of the peptide is QPEIYPTIPSNK.
(DOC)

Figure S12 Product ion spectra of *S. uvarum*-specific peptides characteristic for the Sec63 protein in *Sc/Su* hybrid. Panels A show the product spectrum of the 1152.87 Da peptide. The sequence of the peptide is LLQTPIIVEK. Panel B shows the product spectrum of the 1324.58 Da peptide. The sequence of the peptide is LNDEYTSNEIK. Panel C shows the product spectrum of the 1476.80 Da peptide. The sequence of the peptide is QPLLPTNLIPEDK. Panel D shows the product spectrum of the 1747.07 Da peptide. The sequence of the peptide is LFTLEDSQIGDVLGIK. Panel E shows the product spectrum of the 1868.95 Da peptide. The sequence of the peptide is LFDPYEILGISSSASDR.
(DOC)

Figure S13 The peptide map of the *S. mikatae* Sec63p in *Sc/Sm* hybrids. The peptides common to *S. cerevisiae* and *S. mikatae* species are shown as green and *S. mikatae* specific peptides shown as pink. Unique peptides repeatedly detected in experimental MS repeats are marked with asterisks.
(DOC)

Figure S14 Product ion spectra of *S. mikatae*-specific peptides characteristic for the Sec63 protein in *Sc/Sm* hybrid. Panels A show the product spectrum of the 1338.81 Da peptide. The sequence of the peptide is LNEQYTSDEIK. Panel B shows the product spectrum of the 1348.80 Da peptide. The sequence of the peptide is LTEPQDFESQR. Panel C shows the product spectrum of the 1373.86 Da peptide. The sequence of the peptide is INSNEAIQDAATK. Panel D shows the product spectrum of the 1476.80 Da peptide. The sequence of the peptide is QPLLPTNLIPEDK. Panel E shows the product spectrum of the 1560.97 Da peptide. The sequence of the peptide is QFLPELQPADFEK.
(DOC)

Figure S15 Product ion spectra of *S. cerevisiae* specific peptides characteristic for the Sec66p detected in *Sc/Sm* and *Sc/Su* hybrids. Panel A shows the spectrum of the 960.42 Da peptide detected in *Sc/Sm* hybrid. The sequence of the spectrum is DTLQEAER. Panel B shows the spectrum of the 1832.96 Da peptide detected in *Sc/Su* hybrid. The sequence of the peptide is LIELEFKDTLQEAER. Panel C shows the spectrum of 907.42 Da peptide detected in *Sc/Su* hybrid. The sequence of the peptide is RFETEVK.
(DOC)

Figure S16 Product ion spectra of *S. cerevisiae* and *S. mikatae* specific peptides characteristic for the Sec72p detected in Sc/Sm hybrid. Panel A shows the product spectrum of the 1137.61 Da peptide specific for *S. cerevisiae* Sec72p. The sequence of the peptide is VTLEYNANSK. Panel B shows the product spectrum of the 987.49 Da characteristic for *S. mikatae* Sec72p. The sequence of the peptide is LGQWEEAR. Panel C shows the product spectrum of the 1321.57 Da peptide characteristic for *S. mikatae* Sec72p. The sequence of the peptide is MVTLEYNPNNK.
(DOC)

Figure S17 Product ion spectra of *S. cerevisiae* specific peptides characteristic for the Sec72p detected in *Sc/Su* hybrid. Panel A shows the product spectrum of the 1043.40 Da peptide. The sequence of the peptide is GLALAPEDMK. Panel B shows the spectrum of the 1137.50 Da peptide. The sequence of the peptide is VTLEYNANSK.
(DOC)

Figure S18 The peptide map of the *S. uvarum* Trp3p in *Sc/Su* hybrid. The peptides common for both *S. cerevisiae* and *S. uvarum* species are shown as green and *S. uvarum* specific peptides are shown as pink. Unique peptides detected in different MS repeats are marked with asterisks.
(DOC)

Figure S19 Product ion spectra of *S. uvarum* specific peptides characteristic for the Trp3 protein in *Sc/Su* hybrids. Panel A show the product spectrum of the 1371.97 Da peptide. The sequence of the peptide is NTLLIALSGITTR. Panel B shows the product spectrum of the 1487.83 Da peptide. The sequence of the peptide is DLDMEPLVEVNSK. Panel C shows the product spectrum of the 1627.91 Da peptide detected. The sequence of the peptide is NEGVHGFLVGEALMR. Panel D shows the product spectrum of the 2571.97 Da peptide. The sequence of the peptide is NILSINGGNWEENGSSPSNSILDR.
(DOC)

Figure S20 Product ion spectra of *S. mikatae* specific peptides characteristic for the Ctk2 protein in *Sc/Sm* hybrids. Panels A show the product spectrum of the 1219.83 Da peptide detected. The sequence of the peptide is INTEILENFK. Panel B shows the product spectrum of the 1347.95 Da peptide. The sequence of the peptide is INTEILENFKK. Panel C shows the product spectrum of the 1483.73 Da peptide. The sequence of the peptide is NAGPEFGLPQIADR.
(DOC)

Figure S21 Product ion spectra of *S. cerevisiae* and *S. mikatae* specific peptides characteristic for the Ctk3p detected in *Sc/Sm* hybrid. Panel A shows the spectrum of the 963.75 Da peptide characteristic for *S. cerevisiae* Ctk3p. The sequence of the peptide is ELFLDLSK. Panel B shows the spectrum of the 1406.81 Da peptide characteristic for *S. mikatae* Ctk3p. The sequence of the peptide is TQPTNTNILLHR.
(DOC)

Figure S22 Product ion spectra of *S. uvarum* specific peptides characteristic for the Swi6 protein in *Sc/Su* hybrids. Panels A show the product spectrum of the 1253.77 Da peptide. The sequence of the peptide is LQTDYDGDISK. Panel B shows the product spectrum of the 1517.79 Da peptide. The sequence of the peptide is DYESETIQYNEK. Panel C shows the product spectrum of the 2288.89 Da peptide. The sequence of the peptide is LLFPEIQEMPASLNNESTTR. Panel D shows the product spectrum of the 2486.47 Da peptide. The sequence of the peptide is TAEPIVTFTHDLTSEFLNNPLK.
(DOC)

Figure S23 Peptide map of Swi6p from *S. mikatae* (Panel A) and *S. uvarum* (Panel B) species. The peptides common to *S. cerevisiae* and *S. mikatae* and to *S. cerevisiae* and *S. uvarum* species are shown as green boxes, while *S. mikatae* and *S. uvarum* specific peptides are shown as pink boxes in Panel A and B, respectively. No unique *S. mikatae* species-specific peptide were detected in *Sc/Sm* hybrids, while in *Sc/Su* hybrid background, several unique *S. uvarum* peptides (T17, T22, T47, T60) were detected independently in different biological repeats (marked with asterisks).
(DOC)

Figure S24 The quantitative PCR (qPCR) of *SWI6* alleles in *Sc/Sm* and *Sc/Su* hybrids.
(DOC)

Figure S25 The sequence alignment and gene tree of *SWI6* gene. Sequence alignments of *SWI6* of *S. cerevisiae*, *S. mikatae* and *S. uvarum* (Panel A) and the relative gene tree (Panel B).The *sensu lato* species S. *castelli* was used as outgroup.
(DOC)

Figure S26 Growth curves for strains bearing the different types of *TRP2/TRP3* protein complex in absence (A) and presence (B) of tryptophan.
(DOC)

Figure S27 Quantitative PCR of *TRP2* and *TRP3* alleles in *Sc/Su* hybrid background.
(DOC)

Figure S28 Competition fitness assay between TRP2pSu/TRP3pSc strain and the reference strain in presence (red line) or absence (blue line) of tryptophan. Three biological replicas were tested (A, B and C).
(DOC)

Figure S29 Fitness of *Sc/Su* hybrids carrying a deletion of one of the member of the MBF complex. The construction of *Sc/Su* hybrids carrying different type of deletions of homologous member of the MBF complex is shown in Panel A. The growth of such strains in both YPD and YP-glycerol is shown in Panel B. Deletion of either Mbp1^Su^ or Swi6^Su^ (4 and 5) affect the growth of the hybrids when glycerol is present as sole carbon source.
(DOC)

Figure S30 Fitness of parental strains (*Sc* and *Sm*) and *Sc/Sm* hybrids carrying different combination of members of the MBF complex. The construction of *Sc/Sm* hybrids carrying different type of MBF complexes, either chimeric or uni-specific is shown in Panel A. The growth of such strains in both YPD and YP-glycerol is shown in Panel B. No difference in fitness is detected among hybrids carrying the different complexes (1–5) and the parental strains (*Sc* and *Sm*).
(DOC)

Figure S31 Evolutionary perspective of chimeric protein interaction in hybrids. In yeast hybrids, where two proteomes co-exist, there could be preferential formation of uni-parental protein complexes (A) or the potential to establish chimeric interactions (B). The ability or not to form fully functional chimeric complexes will have an impact on gene loss during genome evolution, and on the adaptability potential of the cells, since different types of complexes can confer diverse phenotypic traits to the hybrids (represented by the different colours of the yeast cell wall), upon which natural selection may act.
(DOC)

Table S1 Protein complexes selected for analysis. The complexes encompass different biological functions and orthologs members have sufficient divergence in terms of tryptic digest profile.
(DOCX)

Table S2 Summary table of the biochemical and MS data for the Sec 62–63 protein complex in the *Sc/Sm* hybrid.
(DOCX)

Table S3 Summary table of biochemical and MS data for the Sec 62–63 protein complex in the *Sc/Su* hybrid.
(DOCX)

Table S4 Summary table of biochemical and MS data for the *TRP2/TRP3* complex in the *Sc/Sm* hybrid.
(DOCX)

Table S5 Summary table of biochemical and MS data for the TRP complex in the *Sc/Su* hybrid.
(DOCX)

Table S6 Summary table of biochemical and MS data for the CTK complex in the *Sc/Sm* hybrid.
(DOCX)

Table S7 Summary table of biochemical and MS data for the CTK complex in the *Sc/Su* hybrid.
(DOCX)

Table S8 Summary table of biochemical and MS data for the MBF protein complex in the *Sc/Sm* hybrid.
(DOCX)

Table S9 Summary table of biochemical and MS data for the MBF protein complex in the *Sc/Su* hybrid.
(DOCX)

Table S10 Targeted MS of *Sm* Swi6p in *Sc/Sm* background. The table includes a selection of peptides specific to Swi6p that were used to determine whether the protein was present. Mass to charge ratios that are underlined and in bold were used to direct the mass spectrometer via an inclusion list.
(DOCX)

Table S11 Summary table of biochemical and MS data for the RAM complex in the *Sc/Sm* hybrid.
(DOCX)

Table S12 Summary table of biochemical and MS data for the RAM complex in the *Sc/Su* hybrid.
(DOCX)

Table S13 Summary table of biochemical and MS data for the KU complex in the *Sc/Sm* hybrid.
(DOCX)

Table S14 Summary table of biochemical and MS data for the KU complex in the *Sc/Su* hybrid.
(DOCX)

Table S15 List of primers for the specific amplification of the 16 *S. cerevisiae* chromosomes.
(DOCX)

Table S16 List of primers for the specific amplification of the 16 *S. mikatae* chromosomes.
(DOCX)

Table S17 List of primers for the specific amplification of the 16 *S. uvarum* chromosomes.
(DOCX)

Table S18 List of primers for Real Time PCR.
(DOCX)
