# Supplementary material for: Chimeric Protein Complexes in Hybrid Species Generate Novel Phenotypes
Source: PLoS Genet. 2013 Oct 3;9(10):e1003836. doi: 10.1371/journal.pgen.1003836 (PMC3789821; doi:10.1371/journal.pgen.1003836)
Supplement: Figure S1 — Hybrid generation and selection on the selective SD medium with urea and G418. List of hybrid strains generated by crossing S. cerevisiae TAP strains with S. mikatae and S. uvarum (Panel A), and manual crossing of S. cerevisiae haploid cells with dissected spores and subsequent selection on SD+G418 (Panel B). Crosses were generated on YPD rich plates and replica plated on selective media. The growth pattern of 2∶2 is expected and was selected for further analysis. (DOC) [file pgen.1003836.s001.doc]

**Figure S1**

**B**

*S. cerevisiae MAT* a

Ascus from *S. mikatae* or *S. uvarum*

Tetrad dissection

a

a

a

a

a

a

α

α

SD G418 selection


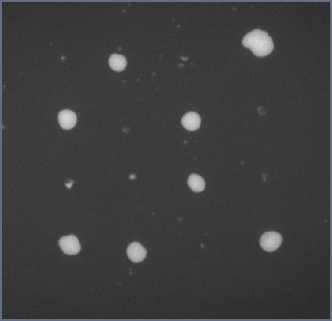


**A**

| **Protein complex tagged** | **EUROSCARF  *S. cerevisiae* TAP-tag strains** | **Species crossed**  **with *S. cerevisiae*** |
| --- | --- | --- |
| **Control (no tag)** | SC0000 | *S. mikatae 1815* |
|  |  | *S. uvarum* NCYC2669 |
| **Sec62-63** | SC0394 | *S. mikatae 1815* |
|  |  | *S. uvarum* NCYC2669 |
| **MBF** | SC0777 | *S. mikatae 1815* |
|  |  | *S. uvarum* NCYC2669 |
| **KU** | SC1097 | *S. mikatae 1815* |
|  |  | *S. uvarum* NCYC2669 |
| **RAM** | SC0821 | *S. mikatae 1815* |
|  |  | *S. uvarum* NCYC2669 |
| **TRP** | SC1662 | *S. mikatae 1815* |
|  |  | *S. uvarum* NCYC2669 |
| **CTK** | SC1218 | *S. mikatae 1815* |
|  |  | *S. uvarum* NCYC2669 |

.
