# Supplementary figures and images for: Chimeric Protein Complexes in Hybrid Species Generate Novel Phenotypes
Source: PLoS Genet. 2013 Oct 3;9(10):e1003836. doi: 10.1371/journal.pgen.1003836 (PMC3789821; doi:10.1371/journal.pgen.1003836)

Figure S2


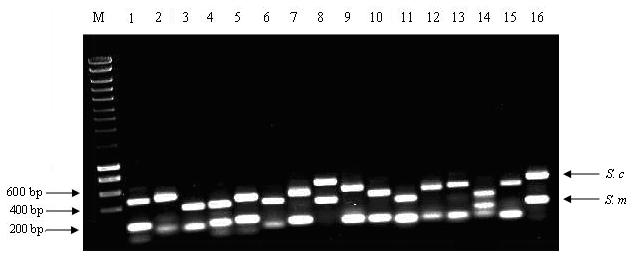

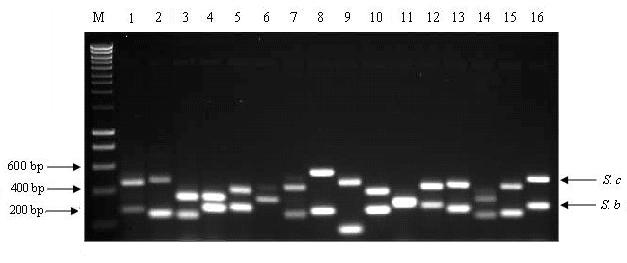


**A**

**B**

M 1 2 3 4 5 6 7 8 9 10 11 12 13 14 15 16

M 1 2 3 4 5 6 7 8 9 10 11 12 13 14 15 16

Supplement: Figure S2 — The chromosomal PCR of Sc/Sm and Sc/Su hybrids. Panel A shows the chromosomal PCR verification of the Sc/Sm hybrid. Panel B shows the chromosomal verification of the Sc/Su hybrids. The PCR was performed with species- specific primers for both parental species for each chromosome. The lane M is the marker Hyperladder I, and the lanes 1–16 show the PCR products corresponding to the 16 chromosomes from S. cerevisiae (higher bands) and S. mikatae or S. uvarum (lower bands). (DOC) [file pgen.1003836.s002.doc]

Figure S3

Dissected Spores

Tetrads *Sc/Sm*


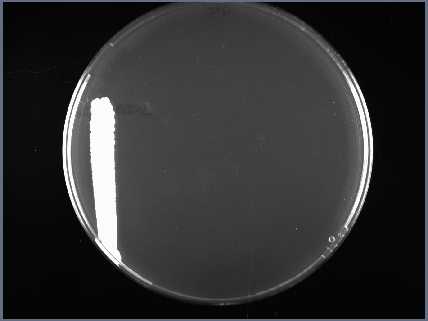

Supplement: Figure S3 — The dissection plate of Sc/Sm 1815 hybrid species. Hybrid Sc/Sm spore dissection plate after 5 days. No viable spores were detected after dissecting 128 tetrads. Similar results were obtained for Sc/Su hybrids (data not shown). (DOC) [file pgen.1003836.s003.doc]

Figure S10

A


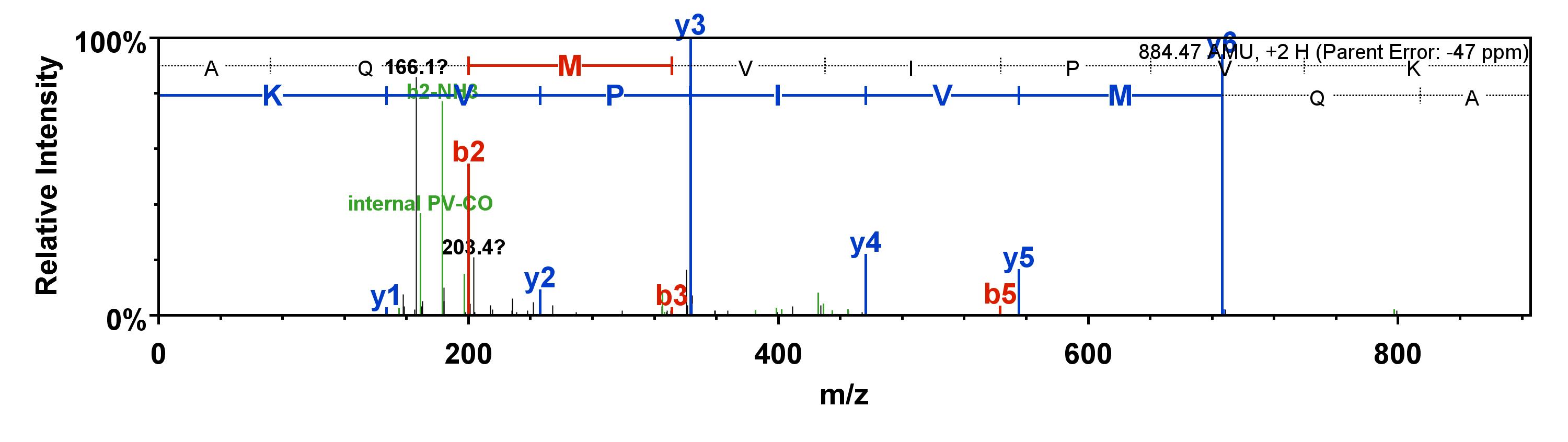


B


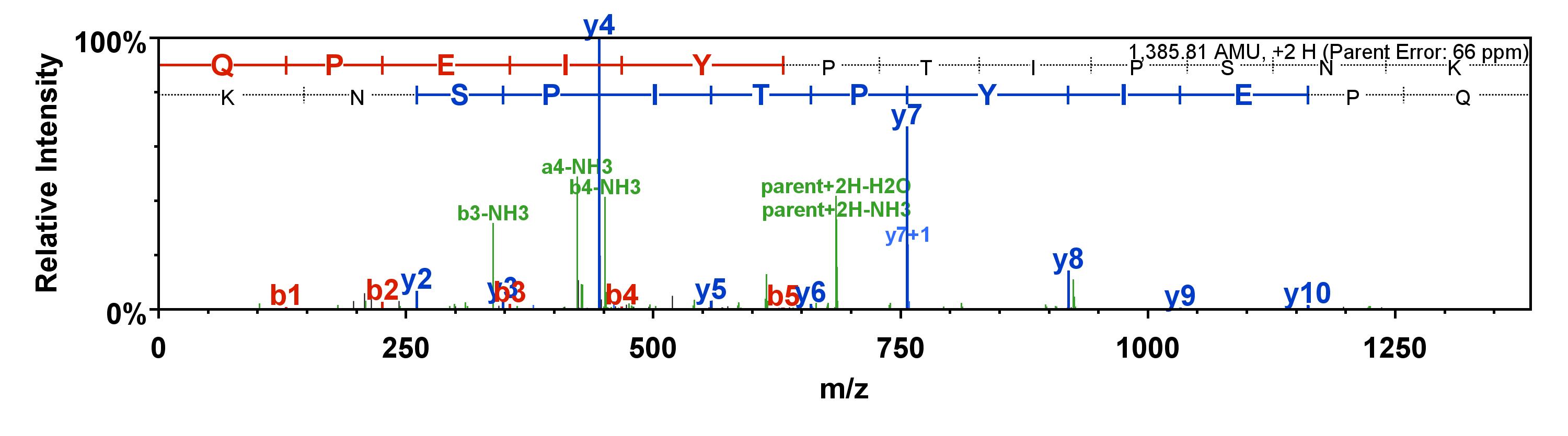

Supplement: Figure S10 — Product ion spectra of S. cerevisiae specific peptides characteristic for the Sec62p detected in Sc/Sm hybrid. Panel A shows the product spectrum of the 884.47 Da peptide. The sequence of the peptide is AQMVIPK. Panel B shows the product spectrum of the 1385.81 Da peptide. The sequence of the peptide is QPEIYPTIPSNK. (DOC) [file pgen.1003836.s010.doc]

Figure S11

A


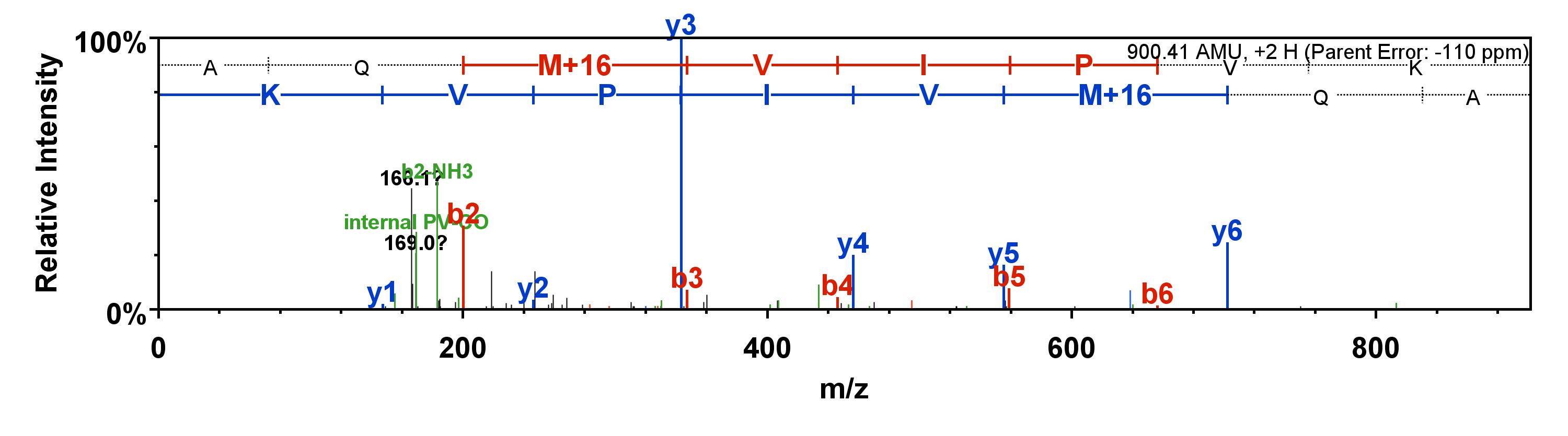


B


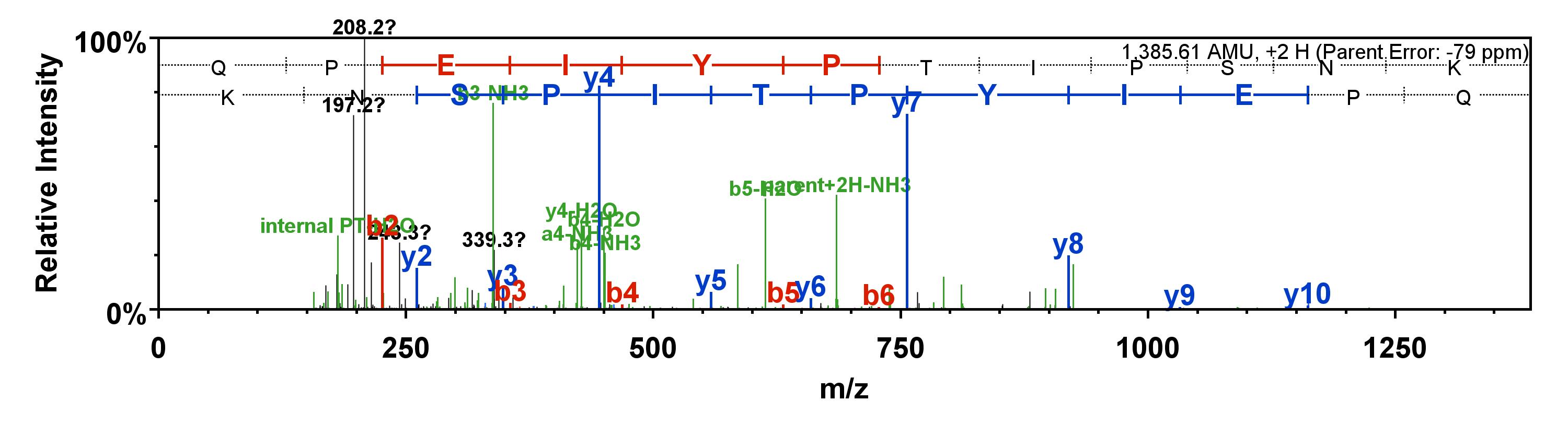

Supplement: Figure S11 — Product ion spectra of S. cerevisiae specific peptides characteristic for the Sec62p detected in Sc/Su hybrid. Panel A shows the product spectrum of 884.47 Da peptide. The sequence of the peptide is AQMVIPK. Panel B shows the product spectrum of 1385.81 Da peptide. The sequence of the peptide is QPEIYPTIPSNK. (DOC) [file pgen.1003836.s011.doc]

Figure S12

A


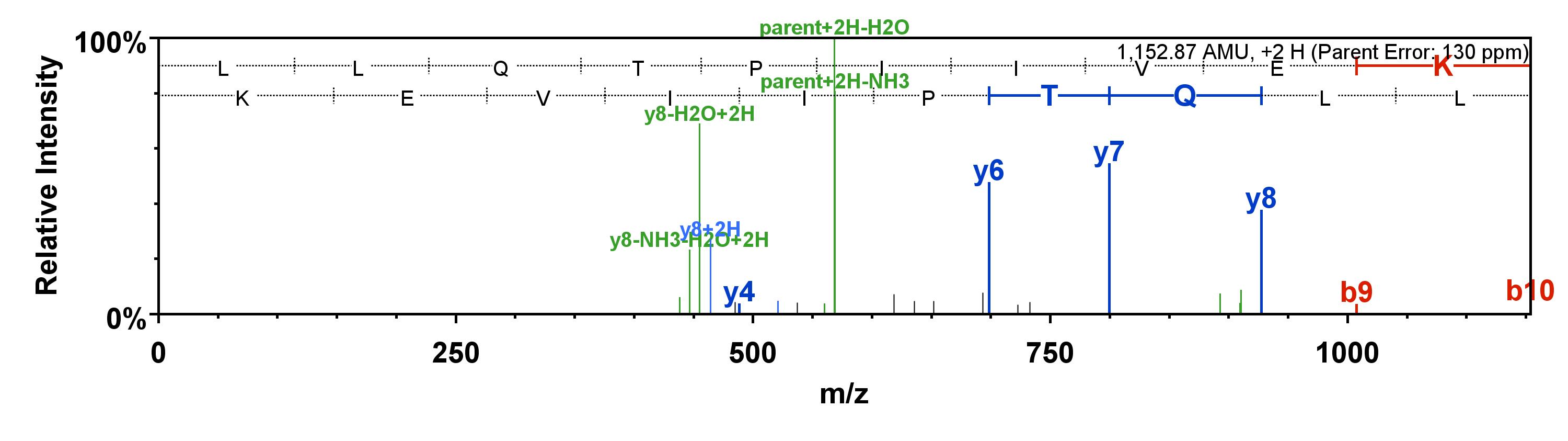


B


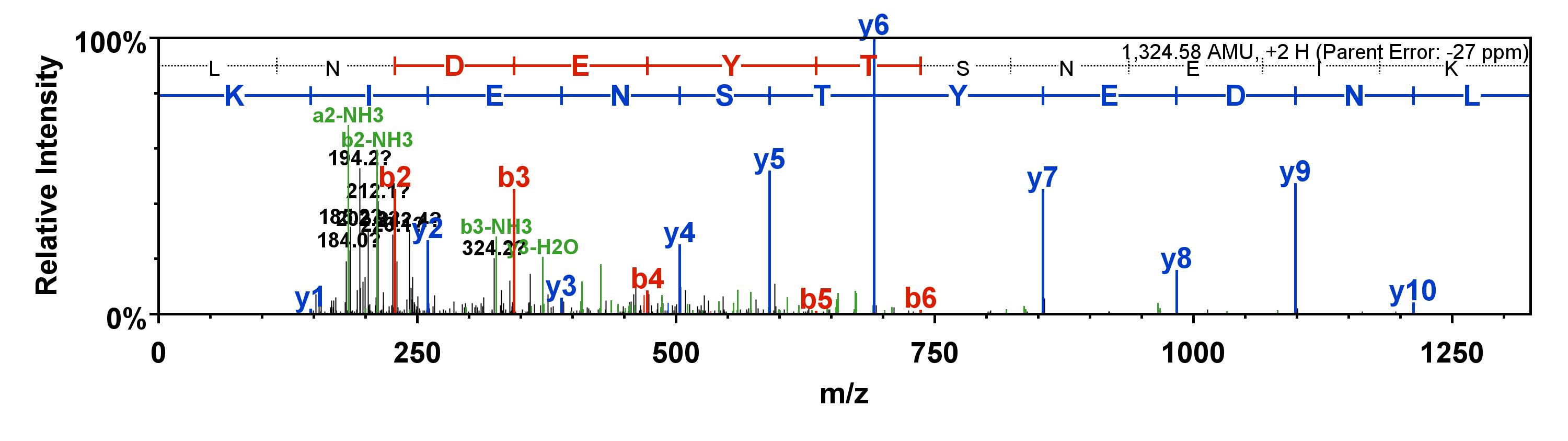


C


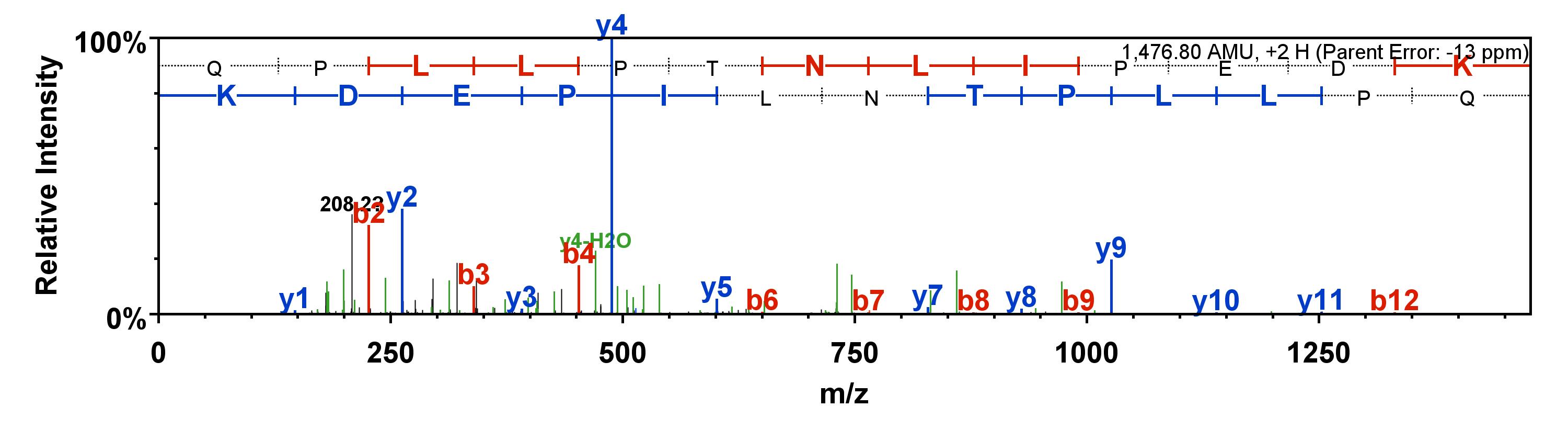


D


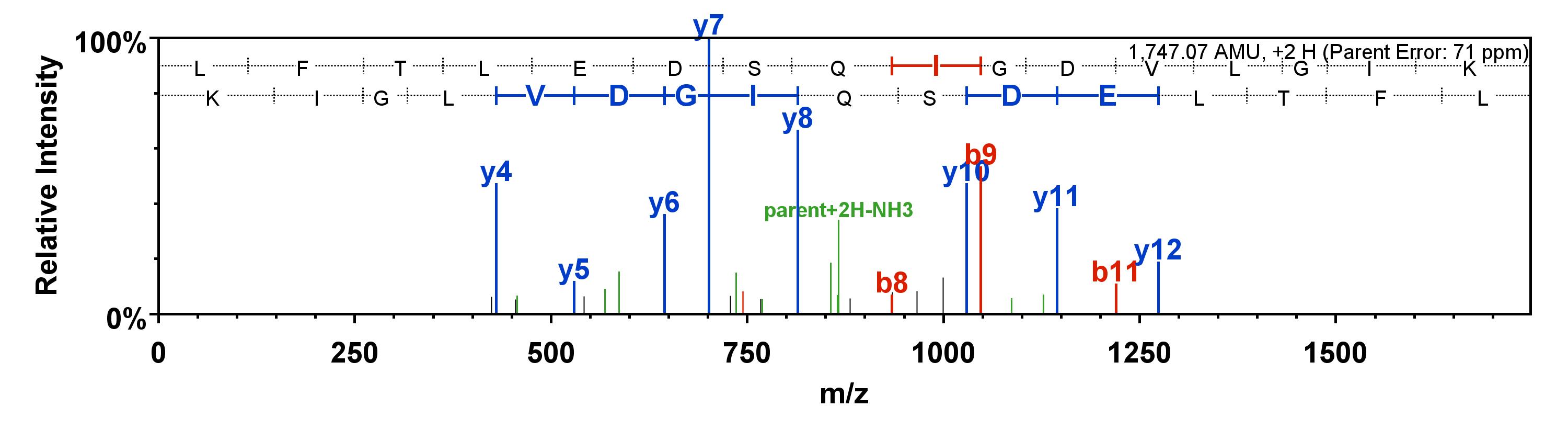


E


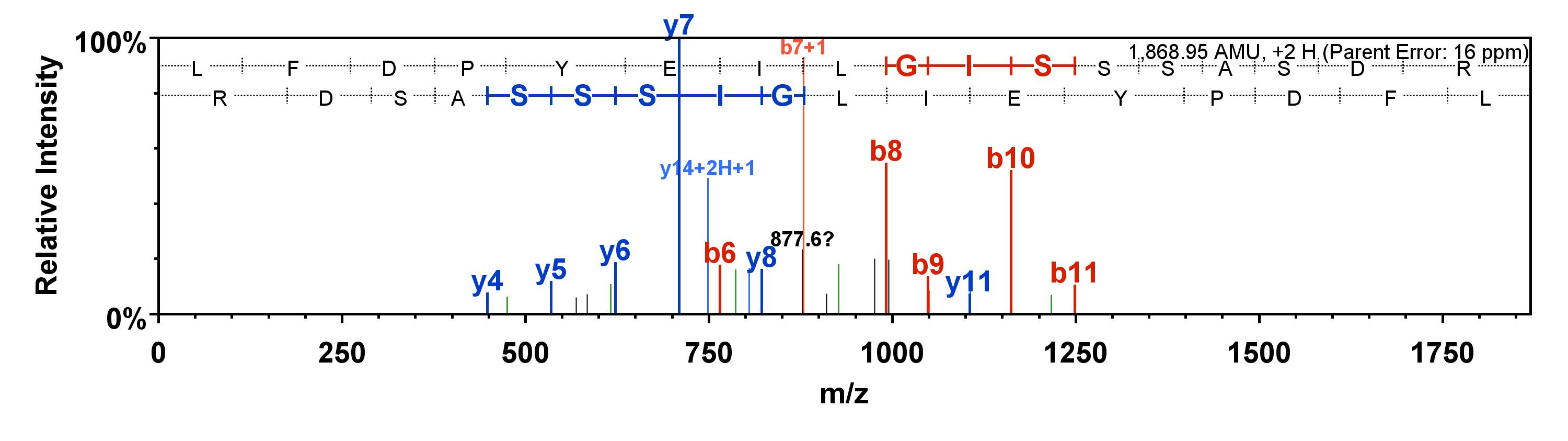

Supplement: Figure S12 — Product ion spectra of S. uvarum-specific peptides characteristic for the Sec63 protein in Sc/Su hybrid. Panels A show the product spectrum of the 1152.87 Da peptide. The sequence of the peptide is LLQTPIIVEK. Panel B shows the product spectrum of the 1324.58 Da peptide. The sequence of the peptide is LNDEYTSNEIK. Panel C shows the product spectrum of the 1476.80 Da peptide. The sequence of the peptide is QPLLPTNLIPEDK. Panel D shows the product spectrum of the 1747.07 Da peptide. The sequence of the peptide is LFTLEDSQIGDVLGIK. Panel E shows the product spectrum of the 1868.95 Da peptide. The sequence of the peptide is LFDPYEILGISSSASDR. (DOC) [file pgen.1003836.s012.doc]

Figure S13


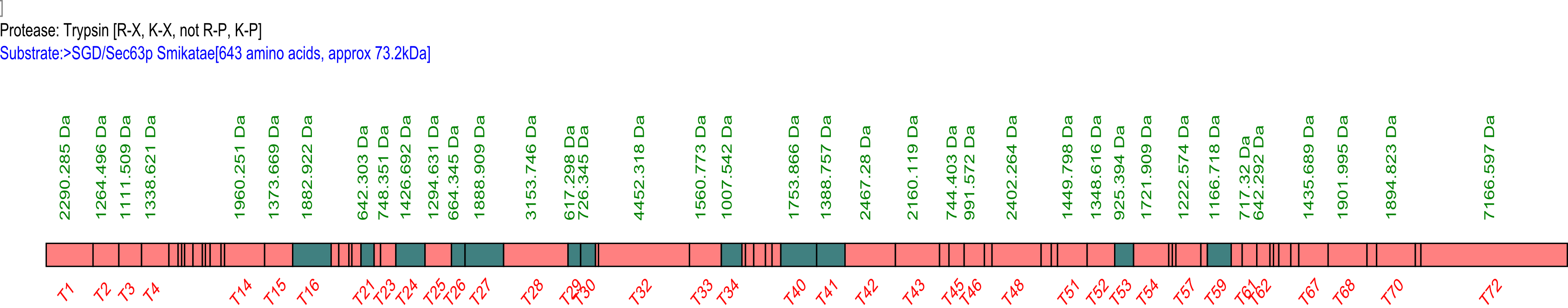


**T15 * * * ***

**T4 * * * ***

**T3 * ***

**T33 * * ***

**T51 * * ***

**T52 * * ***

**T67 ***

**T70 ***

**T16**

**T24**

**T27**

**T34**

**T41**

**T53**

**T59**

Supplement: Figure S13 — The peptide map of the S. mikatae Sec63p in Sc/Sm hybrids. The peptides common to S. cerevisiae and S. mikatae species are shown as green and S. mikatae specific peptides shown as pink. Unique peptides repeatedly detected in experimental MS repeats are marked with asterisks. (DOC) [file pgen.1003836.s013.doc]

Figure S14

A


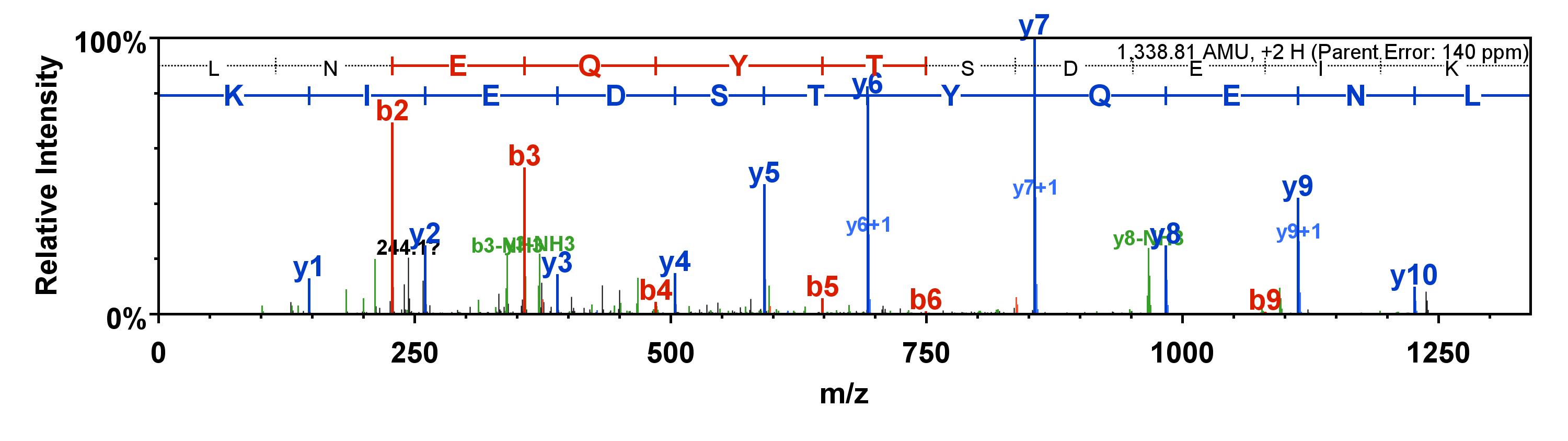


B


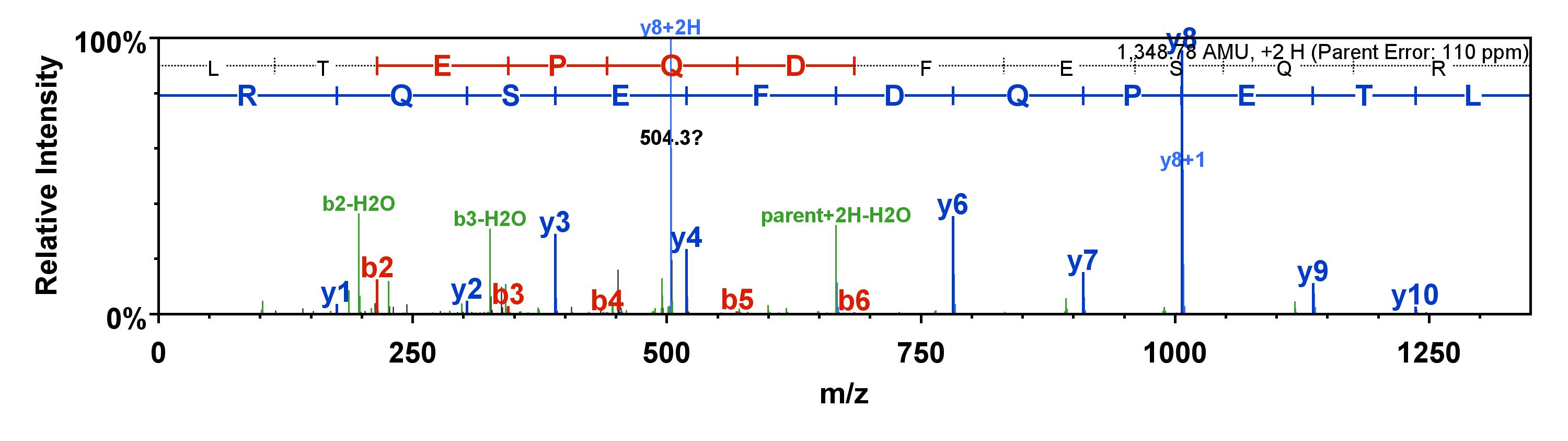


C


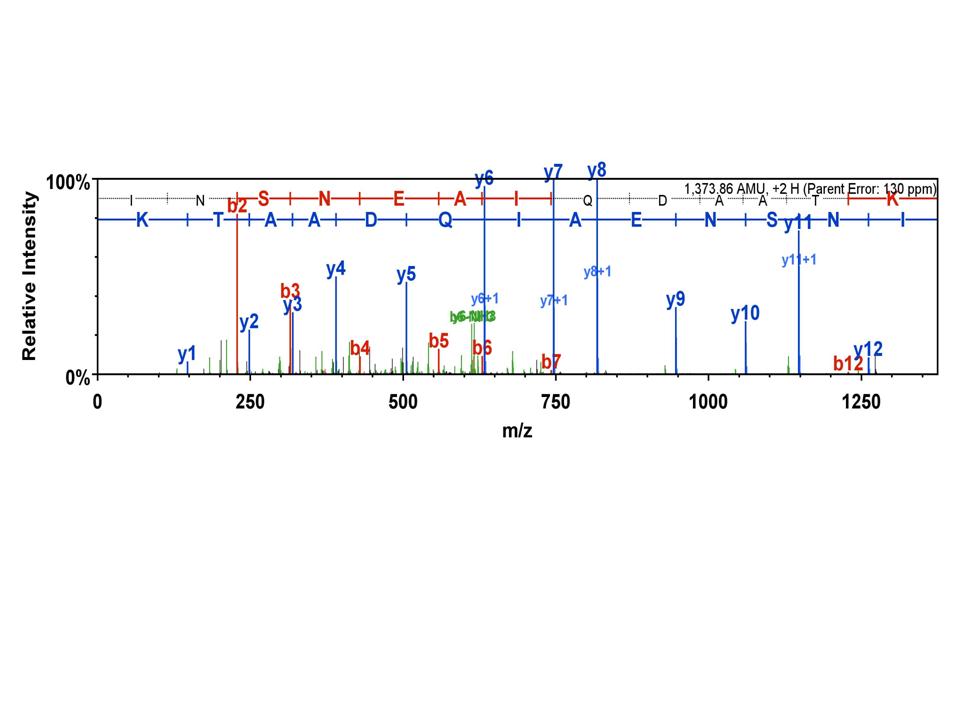


D


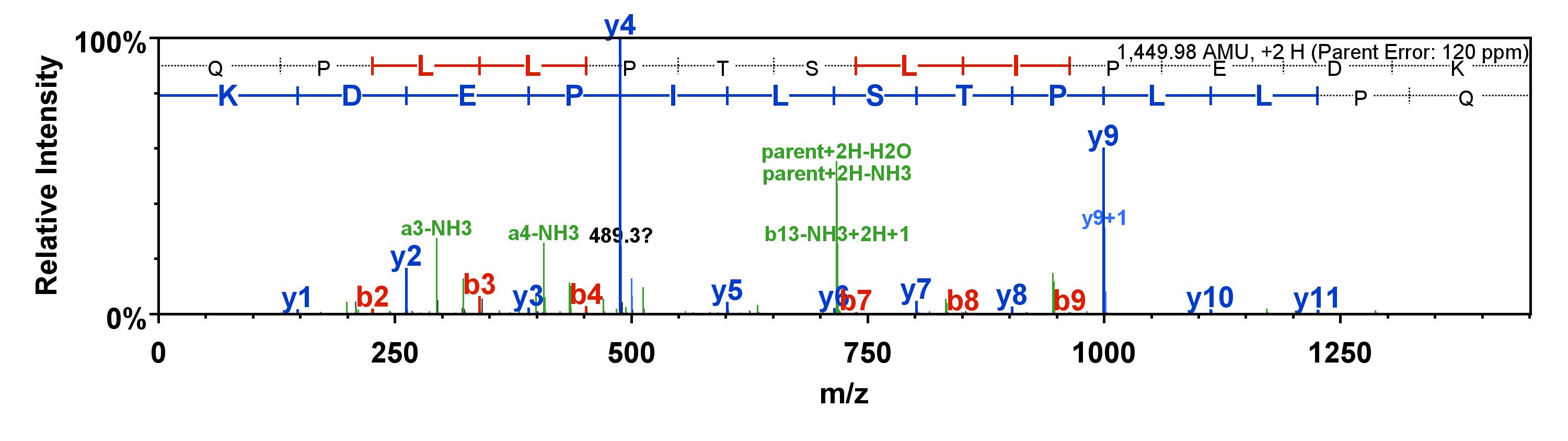


E


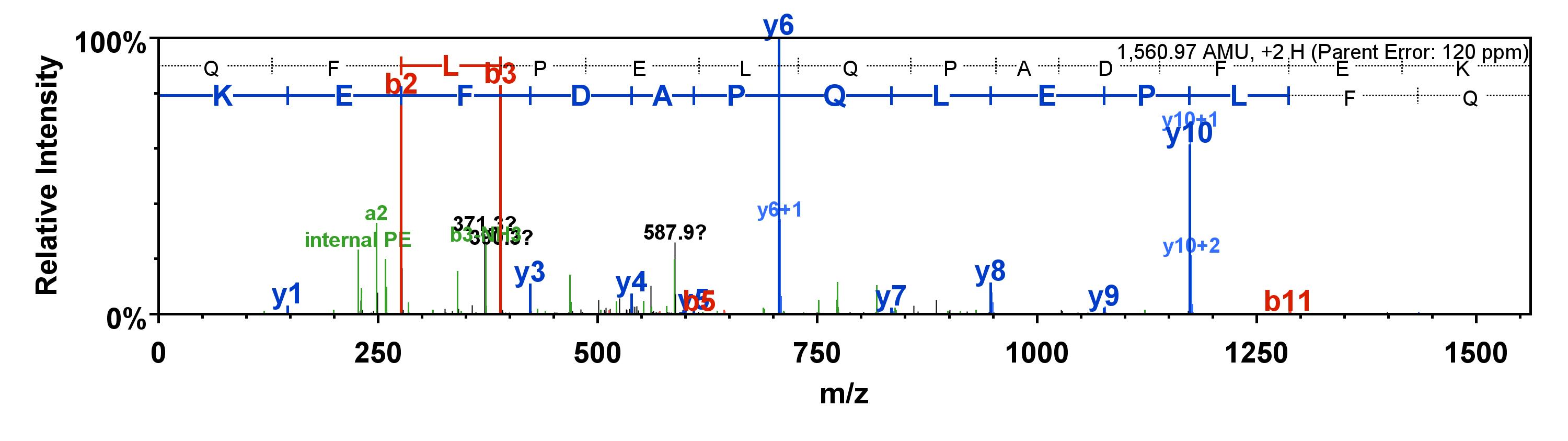

Supplement: Figure S14 — Product ion spectra of S. mikatae-specific peptides characteristic for the Sec63 protein in Sc/Sm hybrid. Panels A show the product spectrum of the 1338.81 Da peptide. The sequence of the peptide is LNEQYTSDEIK. Panel B shows the product spectrum of the 1348.80 Da peptide. The sequence of the peptide is LTEPQDFESQR. Panel C shows the product spectrum of the 1373.86 Da peptide. The sequence of the peptide is INSNEAIQDAATK. Panel D shows the product spectrum of the 1476.80 Da peptide. The sequence of the peptide is QPLLPTNLIPEDK. Panel E shows the product spectrum of the 1560.97 Da peptide. The sequence of the peptide is QFLPELQPADFEK. (DOC) [file pgen.1003836.s014.doc]

Figure S15

A


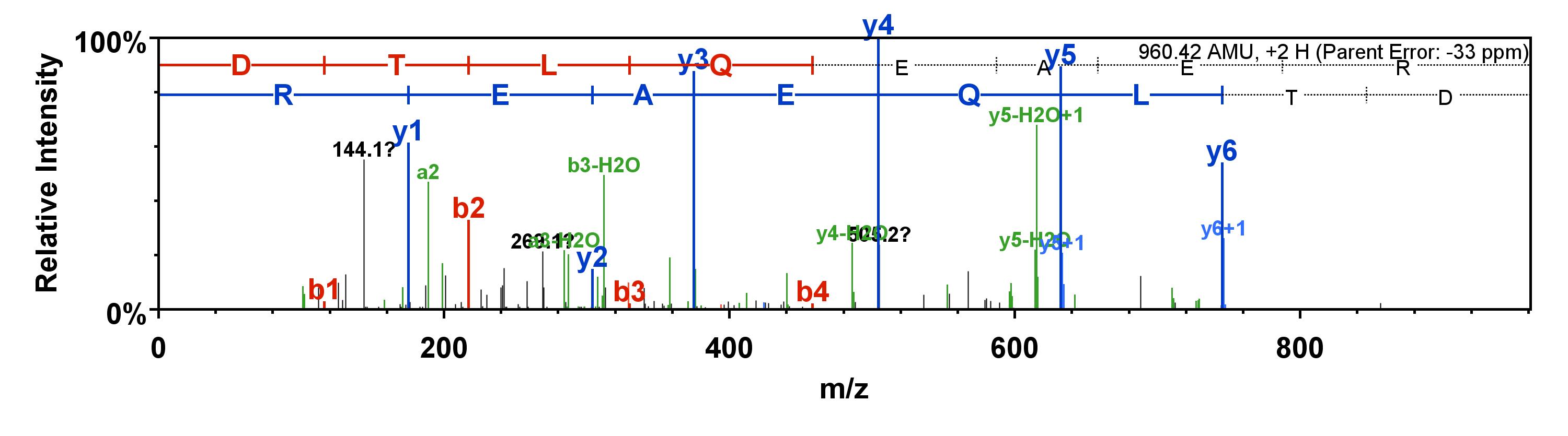


B


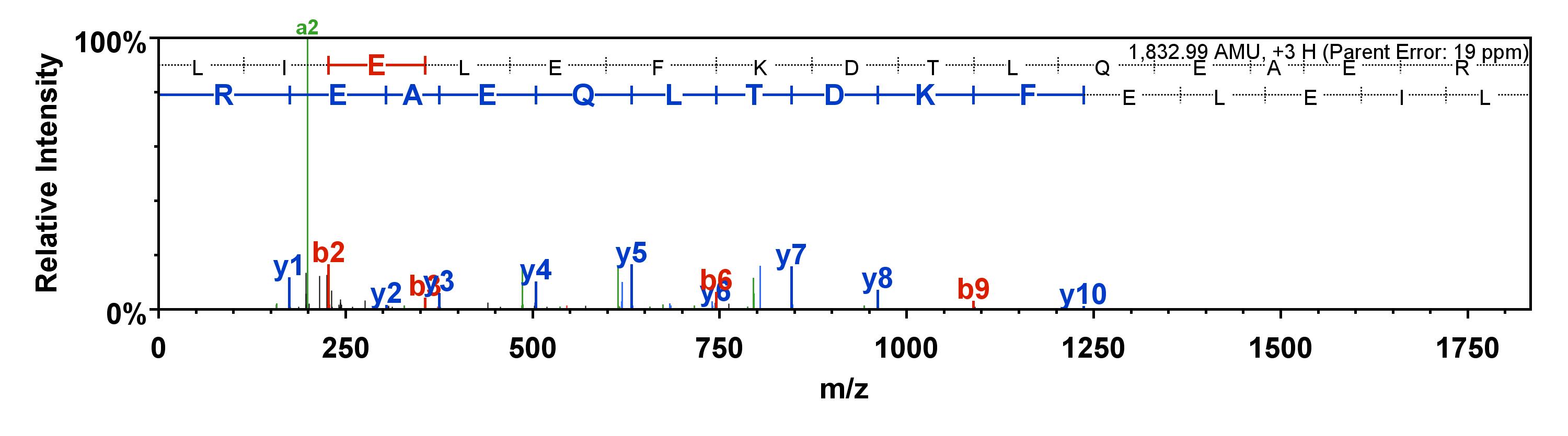


C


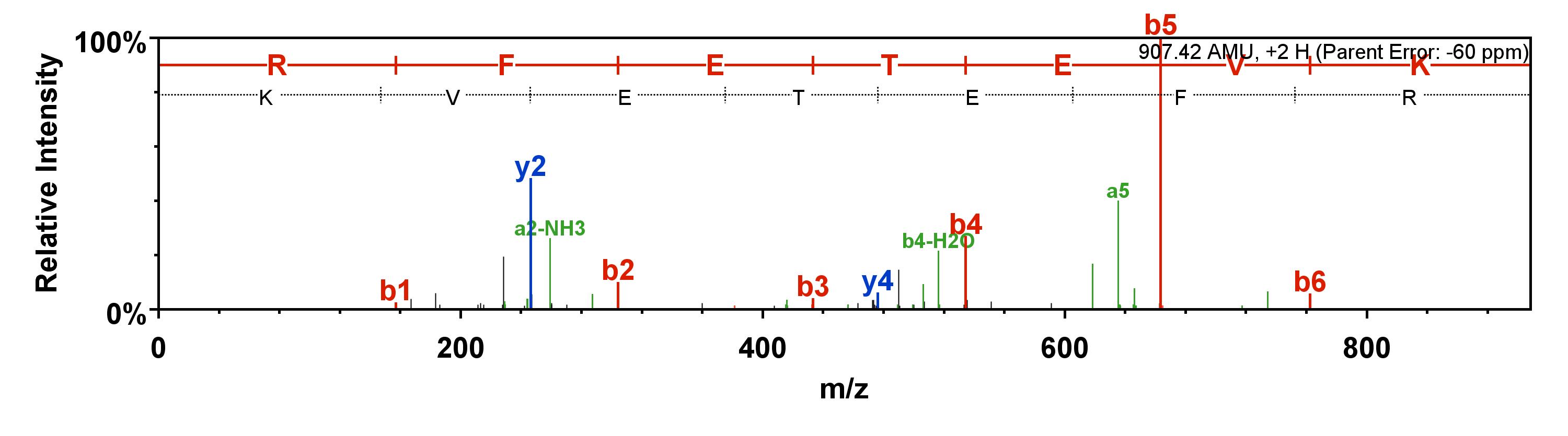

Supplement: Figure S15 — Product ion spectra of S. cerevisiae specific peptides characteristic for the Sec66p detected in Sc/Sm and Sc/Su hybrids. Panel A shows the spectrum of the 960.42 Da peptide detected in Sc/Sm hybrid. The sequence of the spectrum is DTLQEAER. Panel B shows the spectrum of the 1832.96 Da peptide detected in Sc/Su hybrid. The sequence of the peptide is LIELEFKDTLQEAER. Panel C shows the spectrum of 907.42 Da peptide detected in Sc/Su hybrid. The sequence of the peptide is RFETEVK. (DOC) [file pgen.1003836.s015.doc]

Figure S17

A


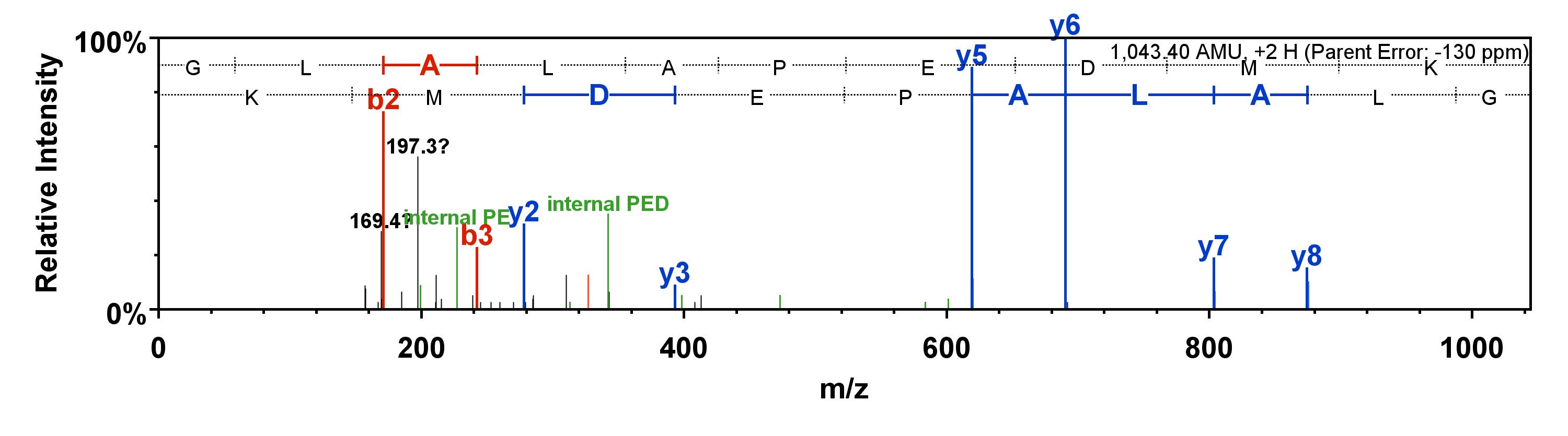


B


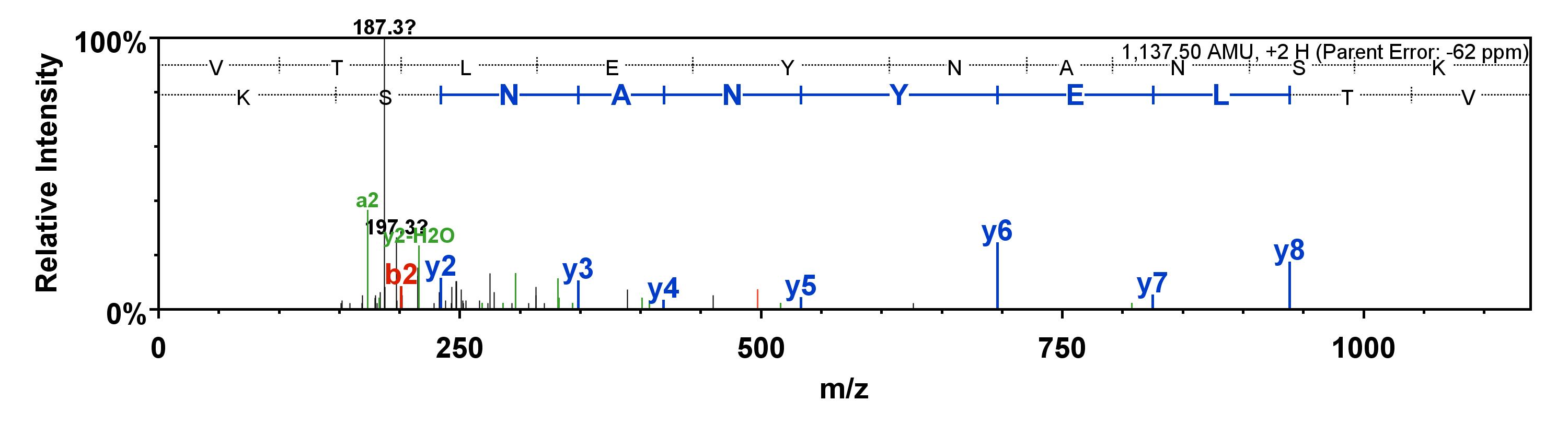

Supplement: Figure S17 — Product ion spectra of S. cerevisiae specific peptides characteristic for the Sec72p detected in Sc/Su hybrid. Panel A shows the product spectrum of the 1043.40 Da peptide. The sequence of the peptide is GLALAPEDMK. Panel B shows the spectrum of the 1137.50 Da peptide. The sequence of the peptide is VTLEYNANSK. (DOC) [file pgen.1003836.s017.doc]

Figure S18


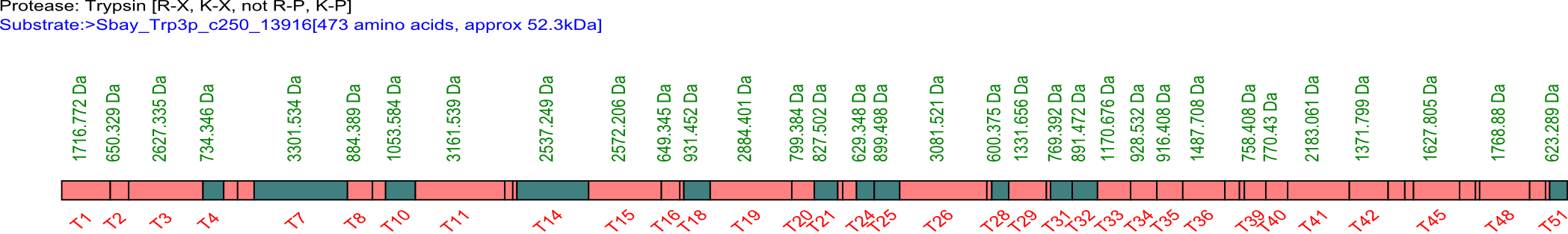


**T15 * * ***

**T36 * * ***

**T4**

**T10**

**T18**

**T25**

**T32**

**T42 * ***

**T45 ***

Supplement: Figure S18 — The peptide map of the S. uvarum Trp3p in Sc/Su hybrid. The peptides common for both S. cerevisiae and S. uvarum species are shown as green and S. uvarum specific peptides are shown as pink. Unique peptides detected in different MS repeats are marked with asterisks. (DOC) [file pgen.1003836.s018.doc]

Figure S19

A


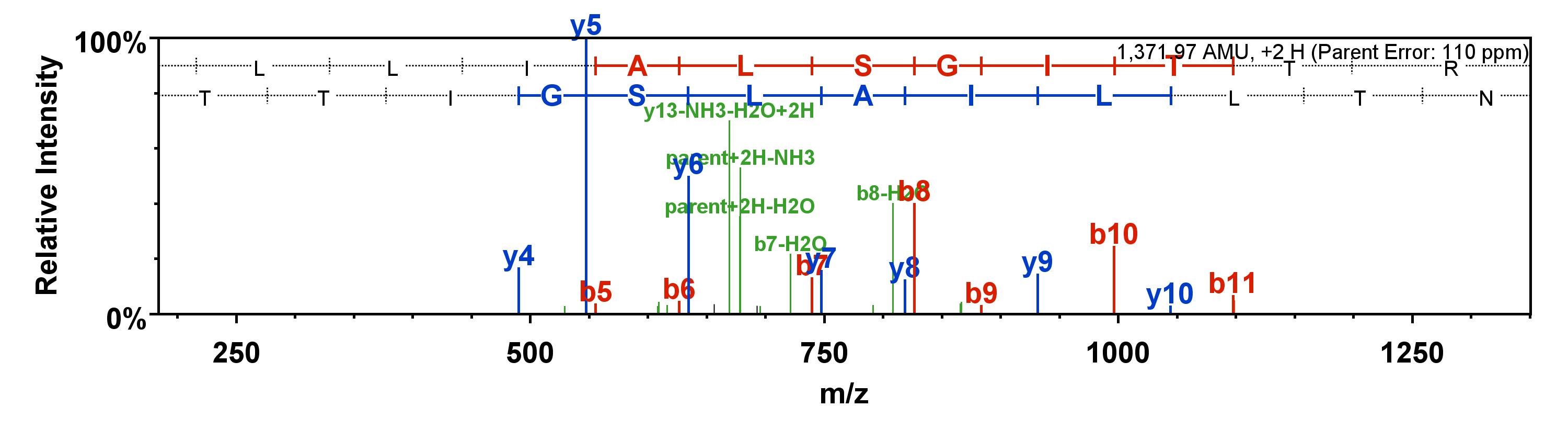


B


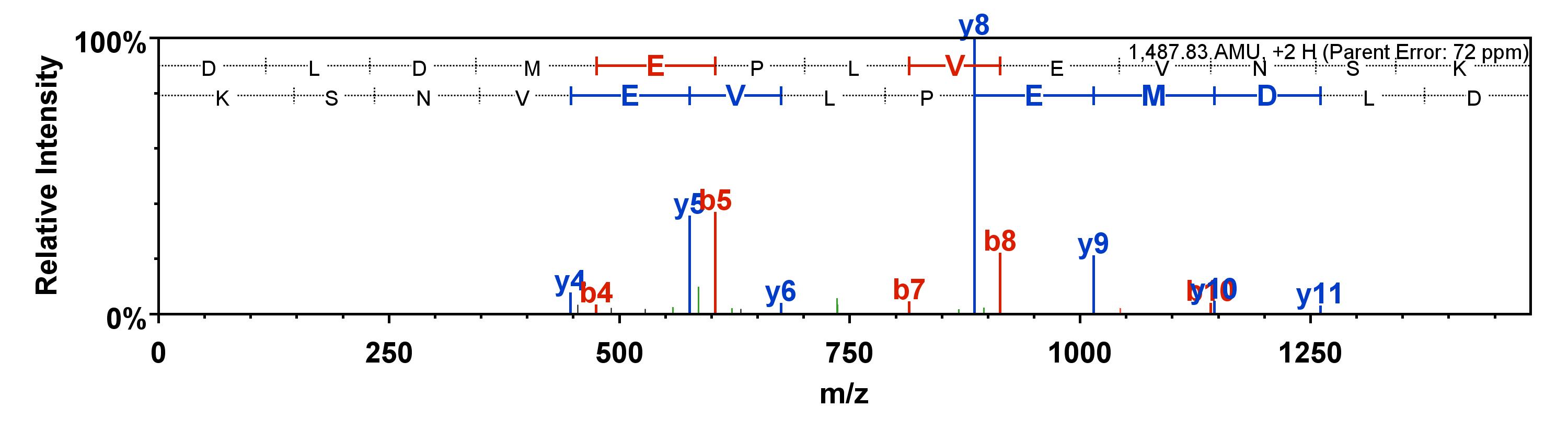


C


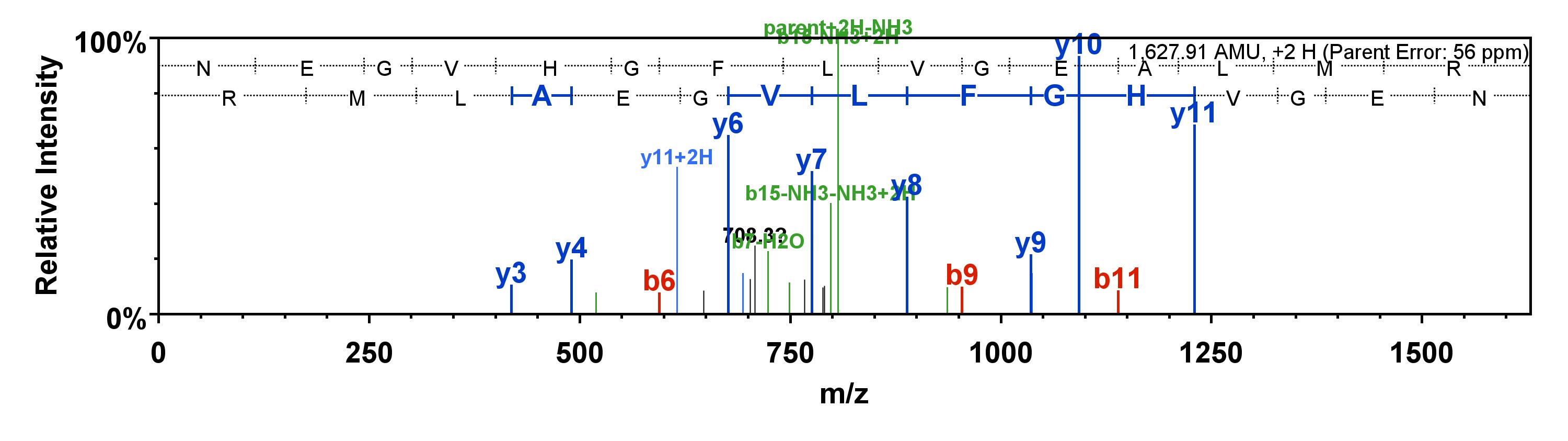


D


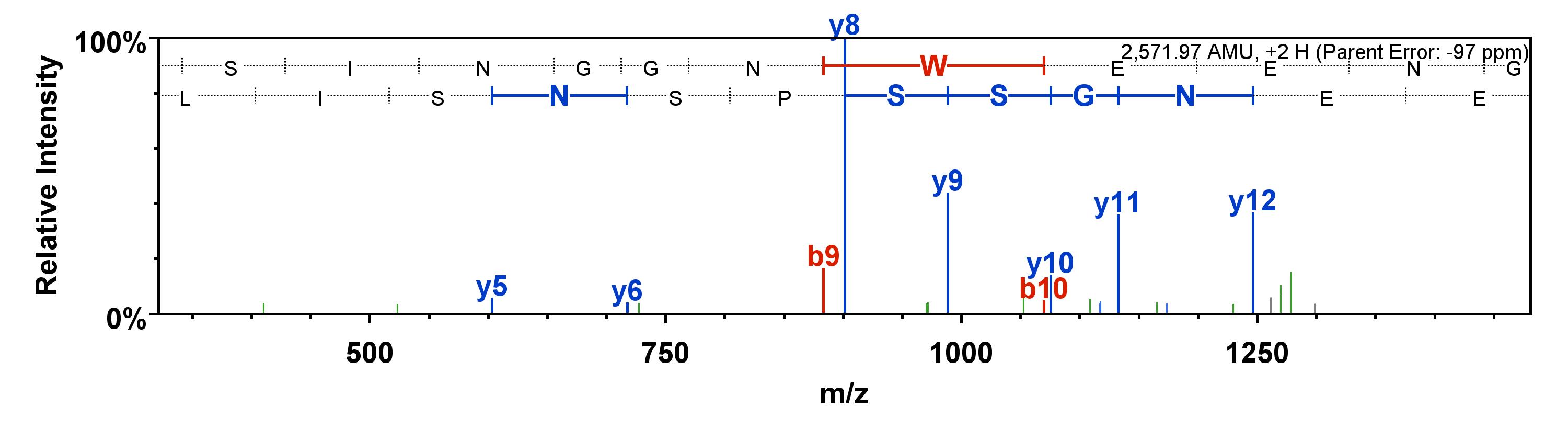

Supplement: Figure S19 — Product ion spectra of S. uvarum specific peptides characteristic for the Trp3 protein in Sc/Su hybrids. Panel A show the product spectrum of the 1371.97 Da peptide. The sequence of the peptide is NTLLIALSGITTR. Panel B shows the product spectrum of the 1487.83 Da peptide. The sequence of the peptide is DLDMEPLVEVNSK. Panel C shows the product spectrum of the 1627.91 Da peptide detected. The sequence of the peptide is NEGVHGFLVGEALMR. Panel D shows the product spectrum of the 2571.97 Da peptide. The sequence of the peptide is NILSINGGNWEENGSSPSNSILDR. (DOC) [file pgen.1003836.s019.doc]

Figure S20

**A**


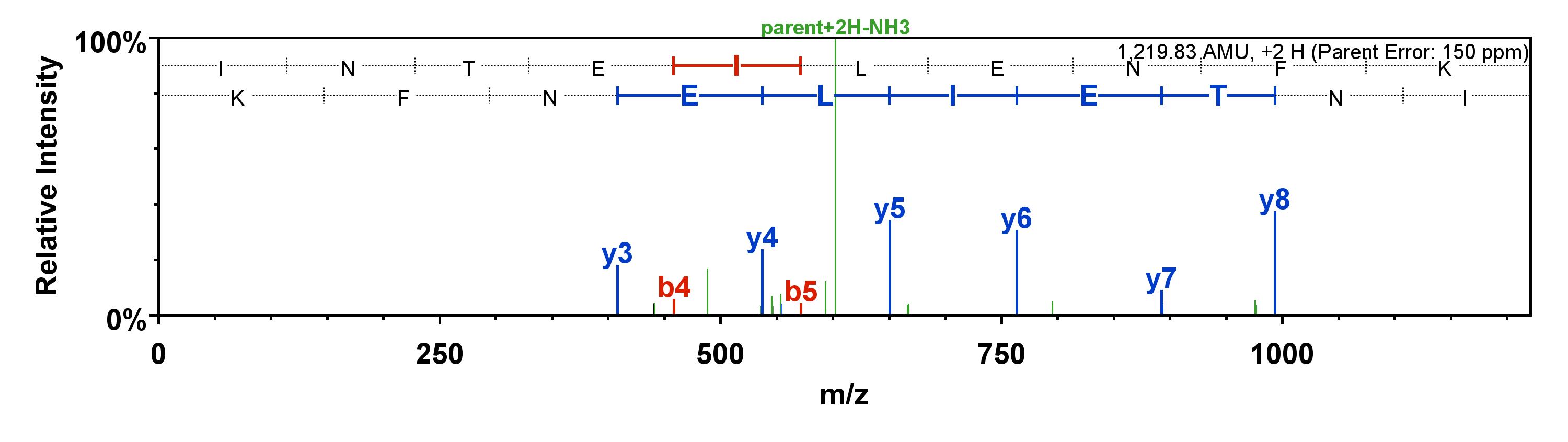


**B**


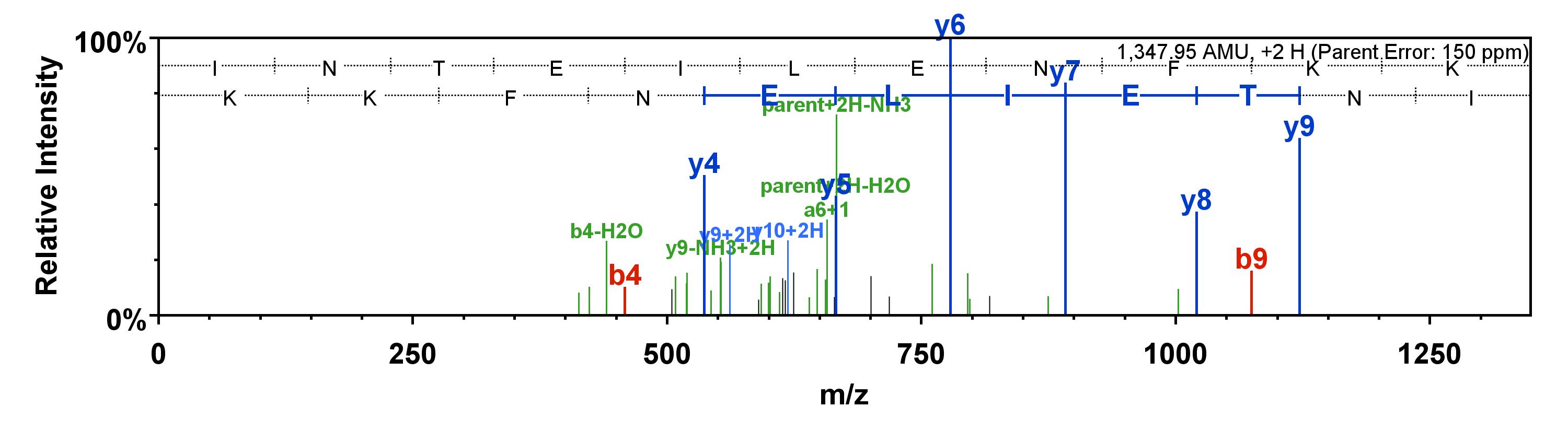


**C**


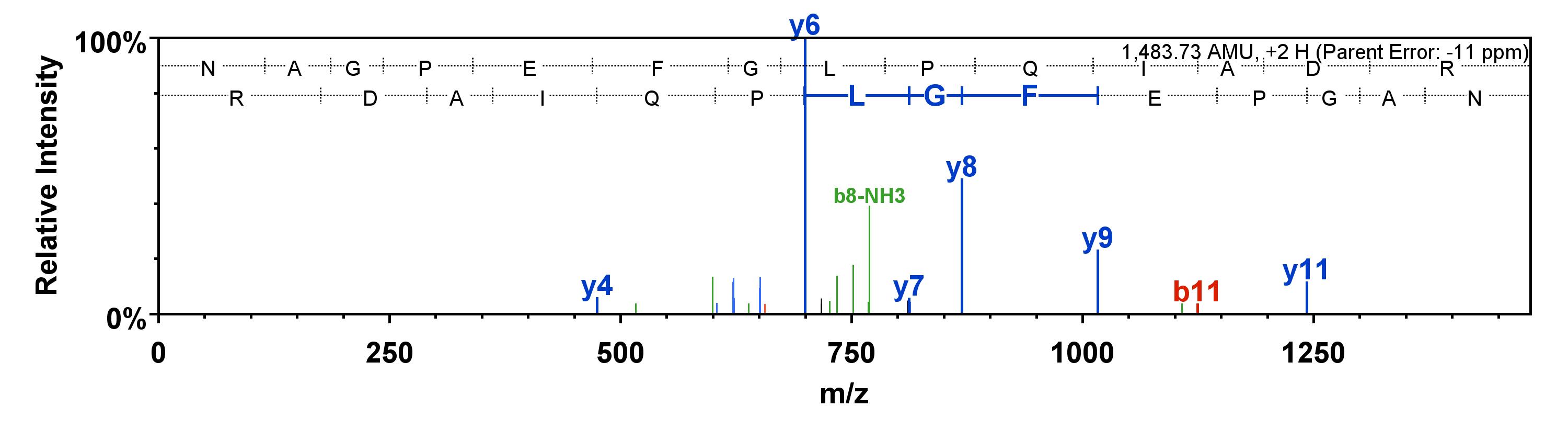

Supplement: Figure S20 — Product ion spectra of S. mikatae specific peptides characteristic for the Ctk2 protein in Sc/Sm hybrids. Panels A show the product spectrum of the 1219.83 Da peptide detected. The sequence of the peptide is INTEILENFK. Panel B shows the product spectrum of the 1347.95 Da peptide. The sequence of the peptide is INTEILENFKK. Panel C shows the product spectrum of the 1483.73 Da peptide. The sequence of the peptide is NAGPEFGLPQIADR. (DOC) [file pgen.1003836.s020.doc]

Figure S21

A


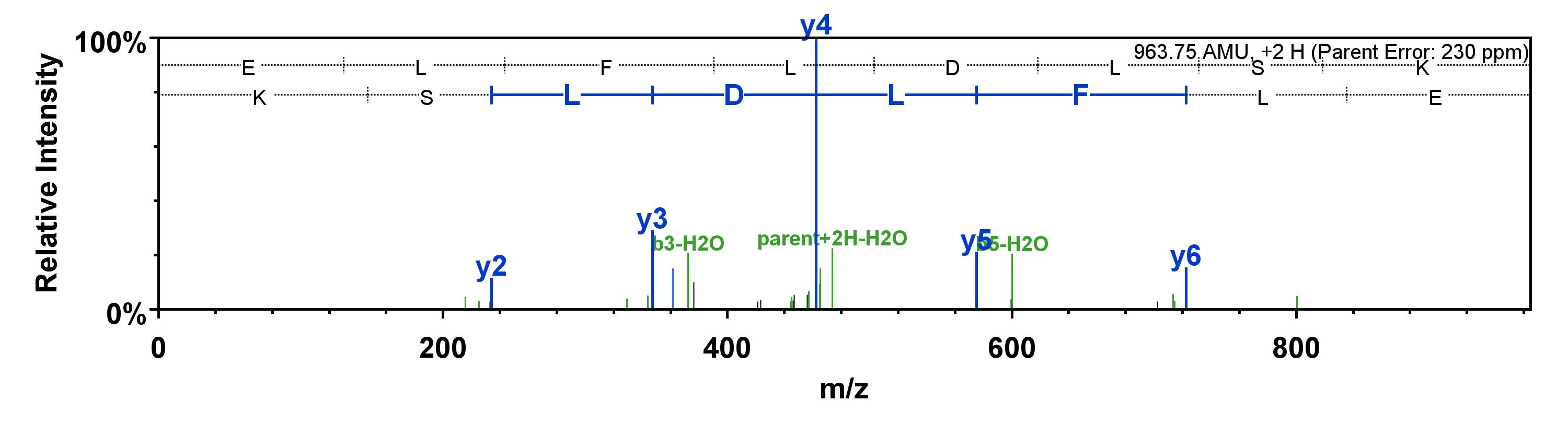


B


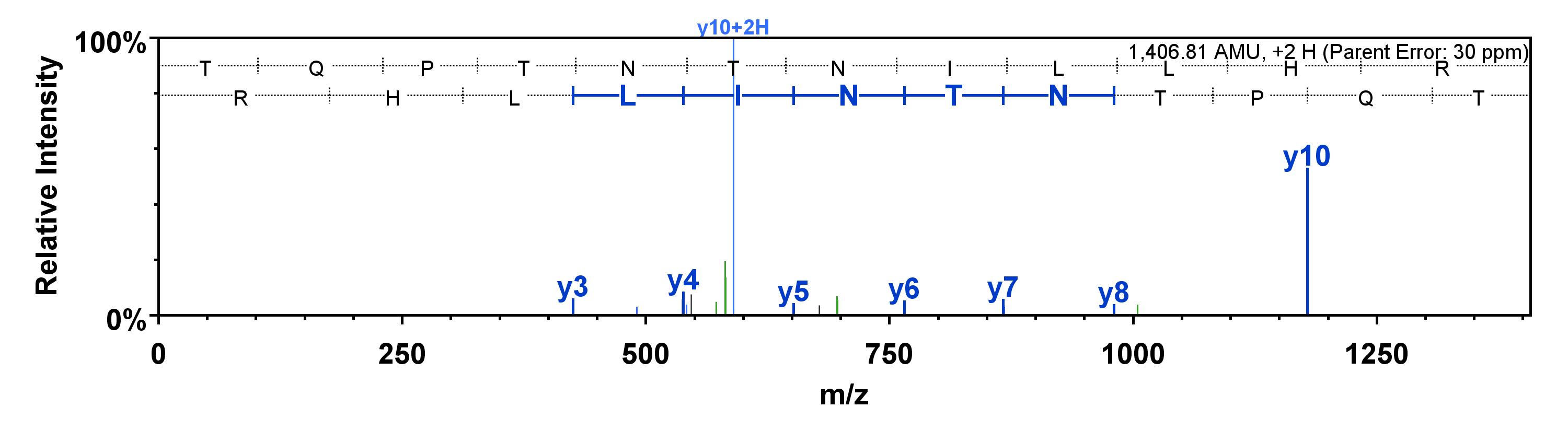

Supplement: Figure S21 — Product ion spectra of S. cerevisiae and S. mikatae specific peptides characteristic for the Ctk3p detected in Sc/Sm hybrid. Panel A shows the spectrum of the 963.75 Da peptide characteristic for S. cerevisiae Ctk3p. The sequence of the peptide is ELFLDLSK. Panel B shows the spectrum of the 1406.81 Da peptide characteristic for S. mikatae Ctk3p. The sequence of the peptide is TQPTNTNILLHR. (DOC) [file pgen.1003836.s021.doc]

Figure S22

**A**


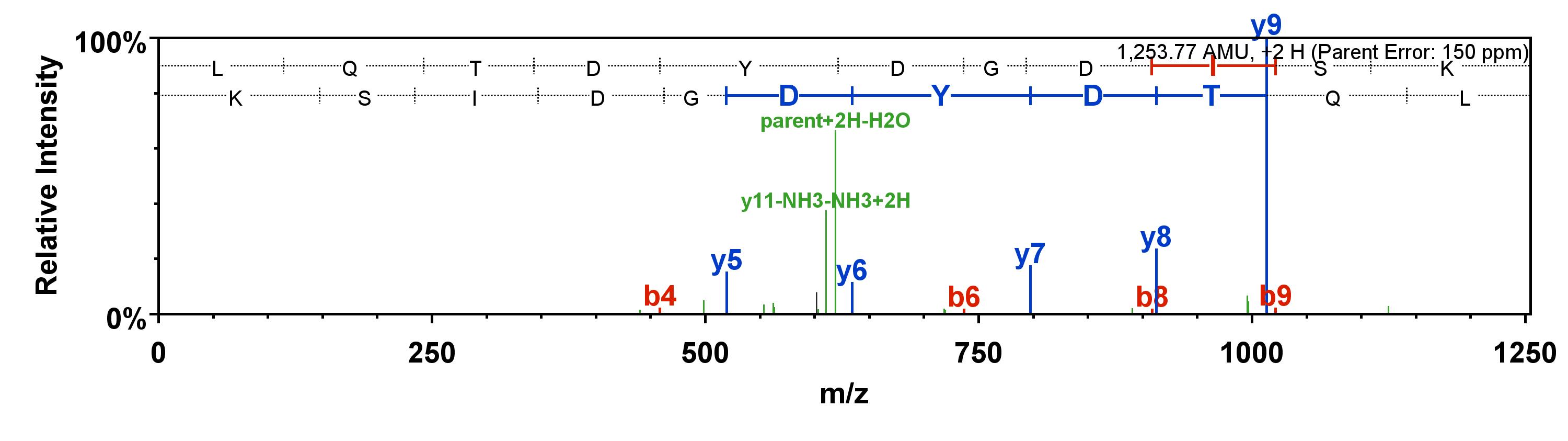


**B**


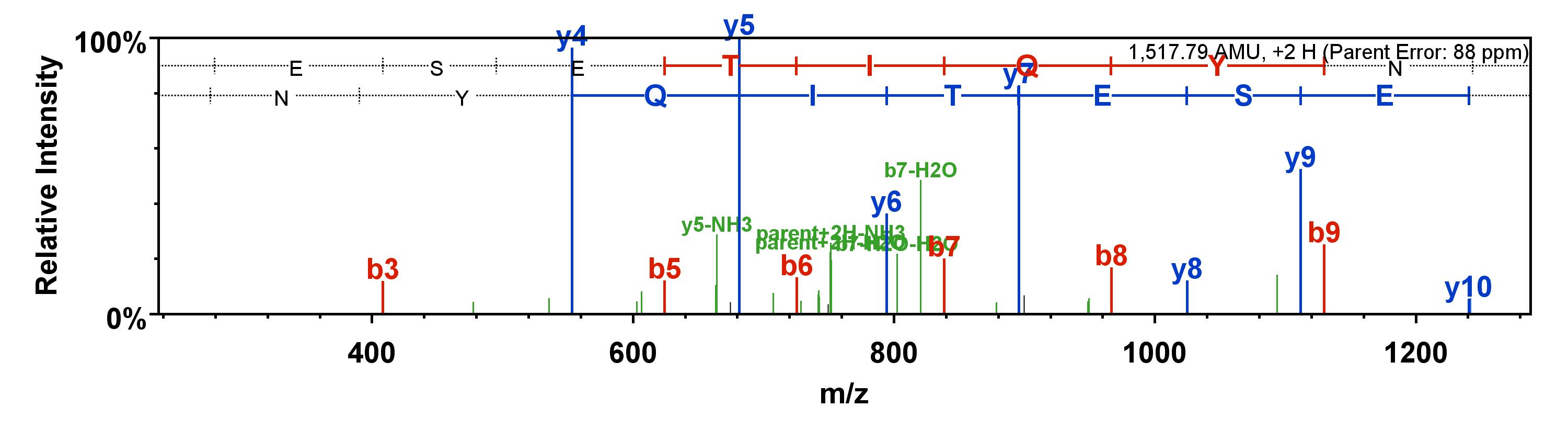


**C**


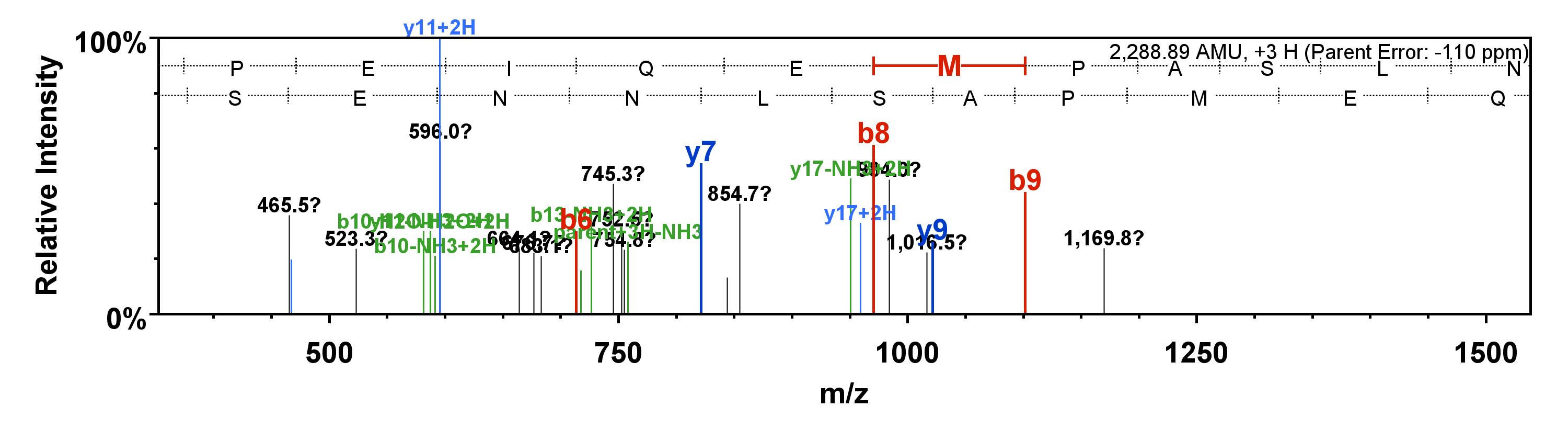


D


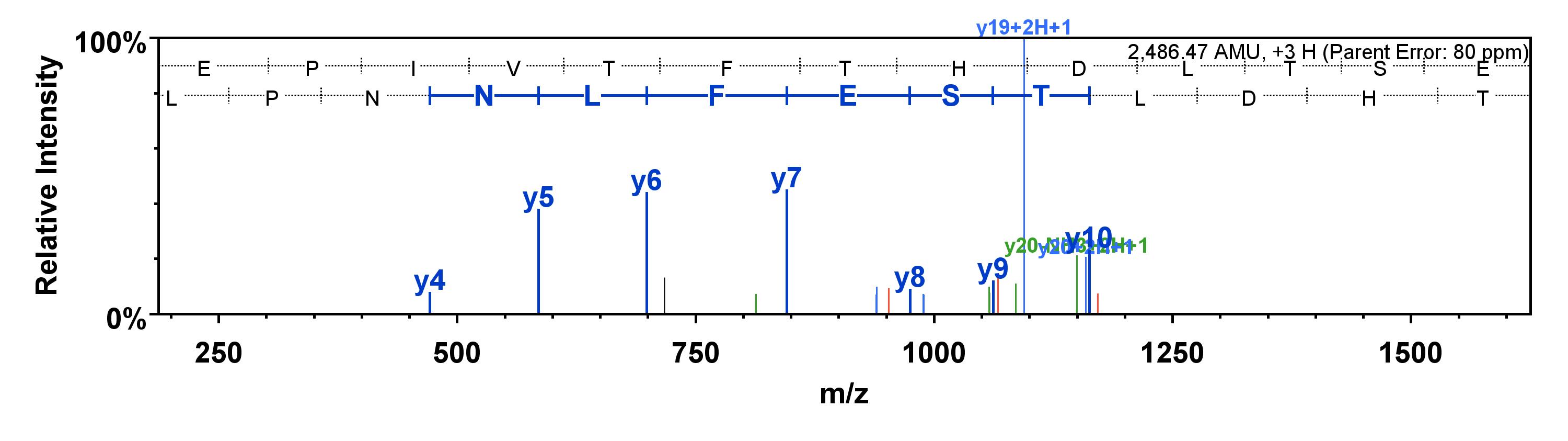

Supplement: Figure S22 — Product ion spectra of S. uvarum specific peptides characteristic for the Swi6 protein in Sc/Su hybrids. Panels A show the product spectrum of the 1253.77 Da peptide. The sequence of the peptide is LQTDYDGDISK. Panel B shows the product spectrum of the 1517.79 Da peptide. The sequence of the peptide is DYESETIQYNEK. Panel C shows the product spectrum of the 2288.89 Da peptide. The sequence of the peptide is LLFPEIQEMPASLNNESTTR. Panel D shows the product spectrum of the 2486.47 Da peptide. The sequence of the peptide is TAEPIVTFTHDLTSEFLNNPLK. (DOC) [file pgen.1003836.s022.doc]

Figure S24

**B**


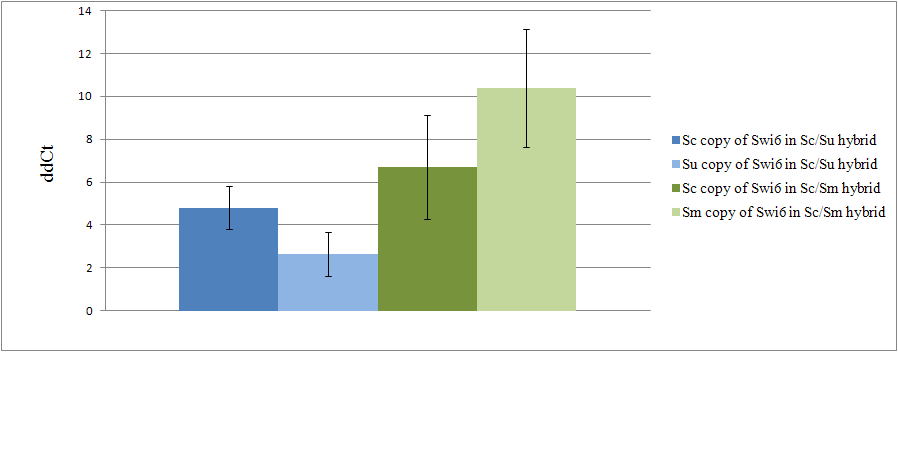


*Sc/Su* hybrid

*Sc/Sm* hybrid

Supplement: Figure S24 — The quantitative PCR (qPCR) of SWI6 alleles in Sc/Sm and Sc/Su hybrids. (DOC) [file pgen.1003836.s024.doc]
