# Supplementary material for: Chimeric Protein Complexes in Hybrid Species Generate Novel Phenotypes
Source: PLoS Genet. 2013 Oct 3;9(10):e1003836. doi: 10.1371/journal.pgen.1003836 (PMC3789821; doi:10.1371/journal.pgen.1003836)
Supplement: Figure S4 — RT-PCR of members of the MBF complex. Panel A shows the amplification of the MBP1 and SWI6 cDNA fragments specific to S. cerevisiae and S. mikatae carried out in the two parental strains and in the hybrid background Sc/Sm. Panel B shows the amplification of the MBP1 and SWI6 cDNA fragments specific to S. cerevisiae and S. uvarum carried out in both parental strains and in the hybrid background Sc/Su. Panel C shows the control for potential cross-hybridization of the species-specific primers. The RT-PCR using the S. cerevisiae MBF specific primers was carried out in either S. mikatae or S. uvarum background (and vice-versa). No cross-hybridization was detected. (DOC) [file pgen.1003836.s004.doc]

Figure S4

**A**

**
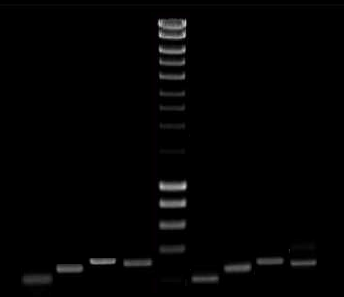
**

*MBP1* Sm

*MBP1* Sc

*MBP1* Sc

*SWI6* Sm

*SWI6* Sc

*SWI6* Sm

M

*MBP1* Sm

*SWI6* Sc

*Sm*

*Sc*

*Sc/Sm*

*MBP1* Sc

*MBP1* Su

*MBP1* Sc

*MBP1* Su

*SWI6* Su

*SWI6* Sc

*SWI6* Su

*SWI6* Sc

**B**

M

**
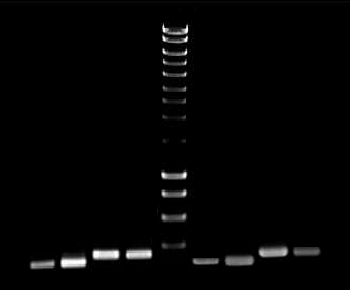
**

**C**

*Sc*

*Su*

*Sc/Su*

*MBP1* Sc

*MBP1* Sc

*MBP1* Sm

*MBP1* Su

*SWI6* Sc

*SWI6* Sm

SWI6 Sc

*SWI6* Su

M

**
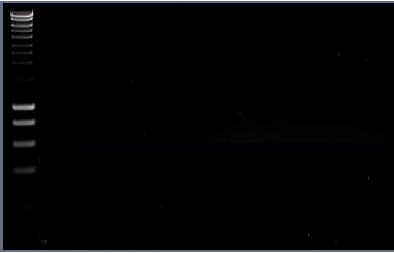
**

*Sm*

*Sc*

*Su*

*Sc*
