# Supplementary material for: Chimeric Protein Complexes in Hybrid Species Generate Novel Phenotypes
Source: PLoS Genet. 2013 Oct 3;9(10):e1003836. doi: 10.1371/journal.pgen.1003836 (PMC3789821; doi:10.1371/journal.pgen.1003836)
Supplement: Figure S5 — RT-PCR of members of the TRP2/TRP3 complex. Panel A shows the amplification of the TRP2 and TRP3 cDNA fragments specific to S. cerevisiae and S. mikatae and S. uvarum carried out in the parental strains and in the hybrid background Sc/Sm and Sc/Su. Panel B shows the control for potential cross-hybridization of the species-specific primers. The RT-PCR using the S. cerevisiae MBF specific primers was carried out in either S. mikatae or S. uvarum background (and vice-versa). No cross-hybridization was detected. (DOC) [file pgen.1003836.s005.doc]

**Figure S5**

*TRP2* Sc

*TRP2* Sm

*TRP3* Sc

*TRP3* Sm

*TRP2* Sc

*TRP3* Sc

*TRP2* Su

*TRP3* Su

*TRP2* Sc

*TRP3* Sc

*TRP2* Sm

*TRP3* Sm

*TRP2* Sc

*TRP3* Sc

*TRP2* Su

*TRP3* Su

M

**
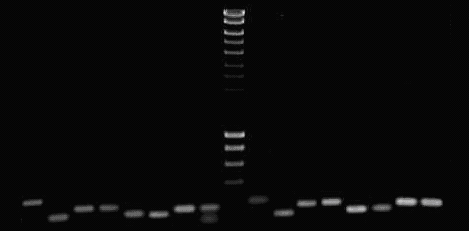
**

**A**

*Sm*

*Sc/Sm*

*Sc/Su*

*Sc*

*Sc*

*Su*

*TRP2* Sc

*TRP3* Sc

*TRP2* Sm

*TRP3* Sm

*TRP2* Sc

*TRP3* Sc

*TRP2* Su

*TRP2* Su

**B**

M

**
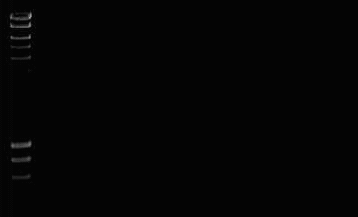
**

*Sm*

*Sc*

*Su*

*Sc*
