# Supplementary material for: Chimeric Protein Complexes in Hybrid Species Generate Novel Phenotypes
Source: PLoS Genet. 2013 Oct 3;9(10):e1003836. doi: 10.1371/journal.pgen.1003836 (PMC3789821; doi:10.1371/journal.pgen.1003836)
Supplement: Figure S6 — RT-PCR of members of the KU complex. Panel A shows the amplification of the KU70 and KU80 cDNA fragments specific to S. cerevisiae and S. mikatae and S. uvarum carried out in the parental strains. Panel B shows the amplification of the Ku70 and KU80 cDNA fragments specific to S. cerevisiae S. mikatae and S. uvarum carried out in both hybrid backgrounds Sc/Sm and Sc/Su. Panel C shows the control for potential cross-hybridization of the species-specific primers. The RT-PCR using the S. cerevisiae KU specific primers was carried out in either S. mikatae or S. uvarum background (and vice-versa). No cross-hybridization was detected. (DOC) [file pgen.1003836.s006.doc]

Figure S6

**A**

*KU80* Su

*KU70* Su

*KU80* Sc

*KU70* Sc

*KU80* Sm

*KU70* Sm

*KU80* Sc

*KU70* Sc

M


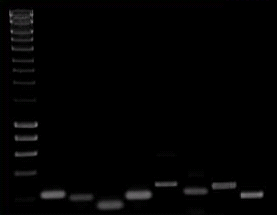


*Su*

*Sc*

*Sm*

*Sc*

**B**

*KU70 Su*

*KU80* Su

*KU70* Sc

*KU80* Sc

*KU80* Sm

*KU70* Sc

*KU80* Sc

*KU70* Sm

M


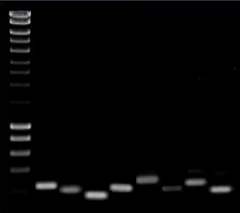


*Sc/Sm*

*Sc/Su*

*KU70* Su

*KU80* Su

**C**

*KU70* Sc

*KU80* Sc

*KU70* Sm

*KU80* Sm

*KU70* Sc

*KU80* Sc

M


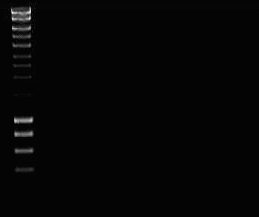


*Sm*

*Sc*

*Su*

*Sc*
