# Supplementary material for: Chimeric Protein Complexes in Hybrid Species Generate Novel Phenotypes
Source: PLoS Genet. 2013 Oct 3;9(10):e1003836. doi: 10.1371/journal.pgen.1003836 (PMC3789821; doi:10.1371/journal.pgen.1003836)
Supplement: Figure S7 — RT-PCR of members of the SEC62–63 complex. Panel A shows the amplification of the SEC62,SEC63, SEC66 and SEC72 cDNA fragments specific to S. cerevisiae, S. mikatae and S. uvarum carried out in the parental strains. Panel B shows the amplification of the SEC62–63 cDNA fragments specific to S. cerevisiae, S. mikatae and S. uvarum carried out in both hybrid backgrounds Sc/Sm and Sc/Su. Panel C shows the control for potential cross-hybridization of the species-specific primers. The RT-PCR using the S. cerevisiae SEC62/63 specific primers was carried out in either S. mikatae or S. uvarum background (and vice-versa). No cross-hybridization was detected. (DOC) [file pgen.1003836.s007.doc]

Figure S7

*SEC62* Sc

*SEC63* Sc

*SEC66* Sc

*SEC72* Sc

*SEC62* Sm

*SEC63* Sm

*SEC66* Sm

*SEC72* Sm

*SEC62* Sc

*SEC63* Sc

*SEC66* Sc

SEC72 Sc

*SEC62* Su

*SEC62* Su

*SEC66* Su

*SEC72* Su

M

**
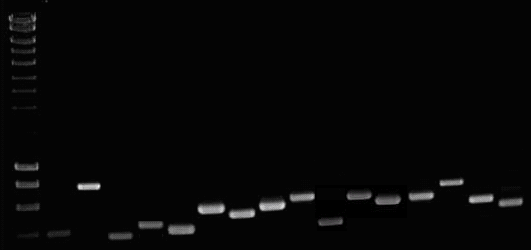
**

**A**

*SEC62* Sc

*SEC63* Sc

*SEC66* Sc

*SEC72* Sc

*SEC62* Sm

*SEC63* Sm

*SEC66* Sm

*SEC72* Sm

*SEC62* Sc

*SEC63* Sc

*SEC66* Sc

SEC72 Sc

*SEC62* Su

*SEC62* Su

*SEC66* Su

*SEC72* Su

*Sc*

*Sc*

*Sm*

*Su*

**B**

M


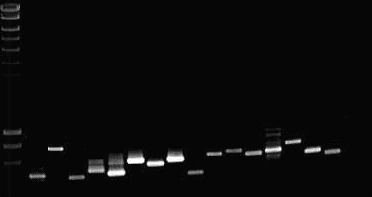


*SEC62* Sc

*SEC63* Sc

*SEC66* Sc

SEC72 Sc

*SEC62* Sm

*SEC63* Sm

*SEC66* Sm

*SEC72* Sm

*SEC62* Sc

*SEC63* Sc

*SEC66* Sc

*SEC72* Sc

*SEC62* Su

*SEC63* Su

*SEC66* Su

*SEC72* Su

*Sc/Su*

*Sc/ Sm*

**C**

M

**
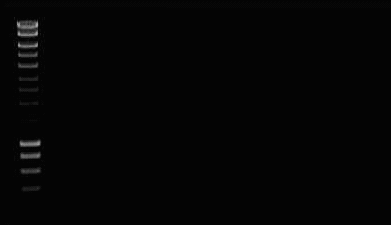
**

*Sm*

*Sc*

*Su*

*Sc*
