# Supplementary material for: Chimeric Protein Complexes in Hybrid Species Generate Novel Phenotypes
Source: PLoS Genet. 2013 Oct 3;9(10):e1003836. doi: 10.1371/journal.pgen.1003836 (PMC3789821; doi:10.1371/journal.pgen.1003836)
Supplement: Figure S8 — RT-PCR of members of the CTK complex. Panel A shows the amplification of the CTK1, CTK2 and CTK3 cDNA fragments specific to S. cerevisiae, S. mikatae and S. uvarum carried out in the parental strains. Panel B shows the amplification of the CTK cDNA fragments specific to S. cerevisiae, S. mikatae and S. uvarum carried out in both hybrid backgrounds Sc/Sm and Sc/Su. Panel C shows the control for potential cross-hybridization of the species-specific primers. The RT-PCR using the S. cerevisiae CTK specific primers was carried out in either S. mikatae or S. uvarum background (and vice-versa). No cross-hybridization was detected. (DOC) [file pgen.1003836.s008.doc]

Figure S8

*CTK1* Sc

*CTK2* Sc

*CTK3* Sc

*CTK1* Sm

*CTK2* Sm

*CTK3* Sm

*CTK1* Sc

*CTK2* Sc

*CTK3* Sc

*CTK1* Su

M


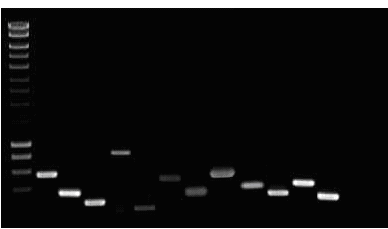


*CTK2* Su

*CTK3* Su

**A**

**B**

*Su*

*Sm*

*Sc*

*Sc*

*CTK1* Sc

*CTK2* Sc

*CTK3* Sc

*CTK1* Sm

*CTK2* Sm

*CTK3* Sm

CTK1 Sc

CTK2 Sc

CTK3 Sc

*CTK1* Su

*CTK2* Su

*CTK3* Su

M


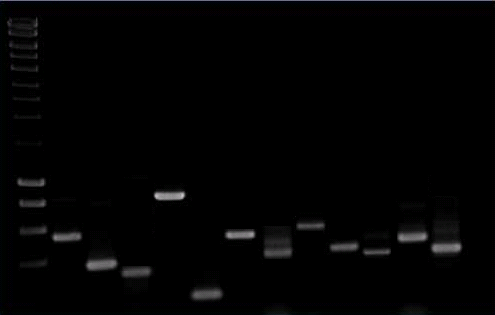


*Sc/Sm*

*Sc/Su*

*CTK1* Sc

*CTK2* Sc

*CTK3* Sc

*CTK1* Sm

*CTK2* Sm

*CTK3* Sm

*CTK1* Sc

*CTK2* Sc

*CTK3* Sc

*CTK1* Su

*CTK2* Su

*CTK3* Su

**C**

M


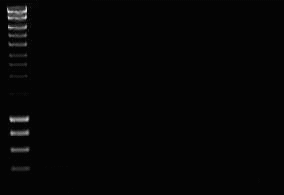


*Sc*

*Sm*

*Sc*

*Su*
