# Supplementary material for: Chimeric Protein Complexes in Hybrid Species Generate Novel Phenotypes
Source: PLoS Genet. 2013 Oct 3;9(10):e1003836. doi: 10.1371/journal.pgen.1003836 (PMC3789821; doi:10.1371/journal.pgen.1003836)
Supplement: Figure S9 — RT-PCR of members of the RAM complex. Panel A shows the amplification of the RAM1 and RAM2 cDNA fragments specific to S. cerevisiae and S. mikatae carried out in the parental strains and in the Sc/Sm hybrid. Panel B shows the amplification of the RAM cDNA fragments specific to S. cerevisiae and S. uvarum carried out in the parental strains and in Sc/Su hybrid. Panel C shows the control for potential cross-hybridization of the species-specific primers. The RT-PCR using the S. cerevisiae RAM specific primers was carried out in either S. mikatae or S. uvarum background (and vice-versa). No cross-hybridization was detected. (DOC) [file pgen.1003836.s009.doc]

Figure S9

*RAM2* Sc

*RAM1* Sc

*RAM2* Sc

*RAM1* Sc

*RAM2* Su

*RAM2* Su

*RAM1* Su

*RAM1* Su

**A**

M


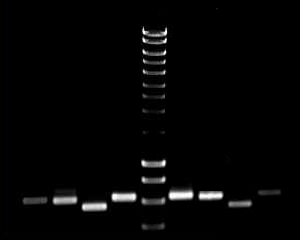


*Sm*

*Sc*

*Sc/Sm*

*RAM2* Sc

*RAM2* Su

*RAM1* Sc

*RAM1* Sc

*RAM2* Sc

*RAM1* Su

*RAM2* Su

*RAM2* Su

**B**

M

**
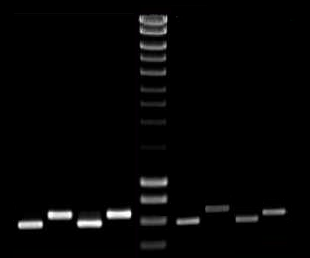
**

*Sc*

*RAM2* Sc

*RAM1* Sc

*RAM2* Sm

*RAM1* Sm

*RAM2* Sc

*RAM1* Sc

*RAM1* Su

*RAM2* Su

*Su*

*Sc/ Su*

**C**

M

**
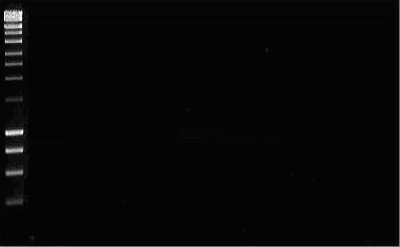
**

*Sm*

*Sc*

*Su*

*Sc*
