# Supplementary material for: Chimeric Protein Complexes in Hybrid Species Generate Novel Phenotypes
Source: PLoS Genet. 2013 Oct 3;9(10):e1003836. doi: 10.1371/journal.pgen.1003836 (PMC3789821; doi:10.1371/journal.pgen.1003836)
Supplement: Figure S16 — Product ion spectra of S. cerevisiae and S. mikatae specific peptides characteristic for the Sec72p detected in Sc/Sm hybrid. Panel A shows the product spectrum of the 1137.61 Da peptide specific for S. cerevisiae Sec72p. The sequence of the peptide is VTLEYNANSK. Panel B shows the product spectrum of the 987.49 Da characteristic for S. mikatae Sec72p. The sequence of the peptide is LGQWEEAR. Panel C shows the product spectrum of the 1321.57 Da peptide characteristic for S. mikatae Sec72p. The sequence of the peptide is MVTLEYNPNNK. (DOC) [file pgen.1003836.s016.doc]

**Figure S16**

A
