# Supplementary material for: Chimeric Protein Complexes in Hybrid Species Generate Novel Phenotypes
Source: PLoS Genet. 2013 Oct 3;9(10):e1003836. doi: 10.1371/journal.pgen.1003836 (PMC3789821; doi:10.1371/journal.pgen.1003836)
Supplement: Figure S23 — Peptide map of Swi6p from S. mikatae (Panel A) and S. uvarum (Panel B) species. The peptides common to S. cerevisiae and S. mikatae and to S. cerevisiae and S. uvarum species are shown as green boxes, while S. mikatae and S. uvarum specific peptides are shown as pink boxes in Panel A and B, respectively. No unique S. mikatae species-specific peptide were detected in Sc/Sm hybrids, while in Sc/Su hybrid background, several unique S. uvarum peptides (T17, T22, T47, T60) were detected independently in different biological repeats (marked with asterisks). (DOC) [file pgen.1003836.s023.doc]

Figure S23
