# Supplementary material for: Chimeric Protein Complexes in Hybrid Species Generate Novel Phenotypes
Source: PLoS Genet. 2013 Oct 3;9(10):e1003836. doi: 10.1371/journal.pgen.1003836 (PMC3789821; doi:10.1371/journal.pgen.1003836)

Figure S25: The sequence alignment and gene tree of *SWI6* gene. Sequence alignments of *SWI6* of *S. cerevisiae*, *S. mikatae* and *S. uvarum* (Panel A) and the relative gene tree (Panel B).The *sensu lato* species S. *castelli* was used as outgroup.

**A**

Scer_SWI6 ---------ATGGCGTTGGAAGAAGTGGTACGATACTTAGGACCTCACAATGAGATCCCT 51

Sbay_SWI6 ---------ATGGCGTTGGAAGAAGTGGTACGATACTTGGGACCTCATAACGAGATCCCT 51

Smik_SWI6 ---------ATGGCATTAGAAGAAGTGGTACGATACTTAGGACCTCATAACGAGATTCCC 51

Scas_SWI6 ATGAGCATCATAGAAGTCACTAAAGTGATTGAAGACCCTGCGTCCTCGGAGAGCGTGTCA 60

** * * ***** * * ** * * * * *

Scer_SWI6 TTGACACTCACTAGAGACTCGGAGACTGGCCATTTCCTCCTGAAACATTTTCTGCCCATT 111

Sbay_SWI6 TTGACGCTGACTAGAGACTCTGAGACTGGCAATTTCCTCCTGAAACATTTCCTACCTATT 111

Smik_SWI6 TTGACACTAACTAGGGACTCTGAGACTGGCCGTTTCCTCCTGAAGCATTTTCTACCCATT 111

Scas_SWI6 TTGACTTTGCAAAGGAATACAGAGACTGGATATTTTCTTCTAAGACCTCTCGT-CCCATT 119

***** * ** * * ******** *** ** ** * * * * * ** ***

Scer_SWI6 TTGCAGCAATATCATGACACGGGGAATATTAACGAGACCAACCCCGATAGTTTCCCCACT 171

Sbay_SWI6 TTGCAGCAGTATCATGATACAGGAAATATAAATGAAACAAACCCCGATAAATTCCCTACT 171

Smik_SWI6 TTGCAACAATACCATGACACGGGGAACATTAACGTGACTAACCCTGATAATTTCCCAACT 171

Scas_SWI6 --GTTATCACATTATGAAGGGAAACGTGTGAACGTGGACCAGAGTGATGAGGTACGTA-- 175

* * **** * ** * * *** * * *

Scer_SWI6 GATGAGGAAAGAAATAAATTACTGGCACATTATGGAATTGCTGTAAATACAGACGACCGA 231

Sbay_SWI6 GATGAGGAAAGAGATAGATTACTGGCTCATTATGAGATTGTTGTGACTACAAACGACCAA 231

Smik_SWI6 GATGAGGACAGGAATAAATTGCTGGCACATTATGGGATTGATGTGACTACCGACAGTCAA 231

Scas_SWI6 -AGGAGGAGGAGGATAGATTATGTGCAAAATATGGGATCCTTGTTGATACTGATAAGGAC 234

* ***** *** *** ** * **** ** *** *** *

Scer_SWI6 GGTGAGTTATGGAT-AGAGCTGGAAAAATGTTTACAATTATTAAACATGCTAAATTTATT 290

Sbay_SWI6 GGTGAGTTATGGAT-AGAATTAGAGAAATGTTTGCAATTATTAAACATGTTAAATCTGTT 290

Smik_SWI6 GGTGAGCTATGGAT-AGAATTGGAAAAATGCTTACAATTATTAAACATGTTGAATTTATT 290

Scas_SWI6 GGTGAGAAATGGATTACTAGTGATAAAGTGTTT-CAGCTGCTTGATATGTTGAATCTTTT 293

****** ****** * * ** ** ** ** * * * *** * *** * **

Scer_SWI6 CGGTTTGTTCCAGGATGCATTCGAATTTGAGGAGCCTGAAACAGATCAGGATGAAGAAGA 350

Sbay_SWI6 TGCTTTGTTCCAGGACGCATTTGAATTCGAAGAGCCTGAAACAGATCAGGATGAGGAGGA 350

Smik_SWI6 TGGTCTGTTTCAGGACGCATTTGAGTTTGAGGAGCCTGAAACAGATCAAGATGAAGAGGA 350

Scas_SWI6 GGATTTGTTTAAAGATGATTTTGAT--------GCCTTGAACGTGACTGG-TGAACAAGA 344

* * **** * ** * ** ** **** *** * * *** * **

Scer_SWI6 ---TCCCAGCCATTCGAAACTACCAGAAAACAAGACCAAAAGTGAAAATTCTAAGGATAA 407

Sbay_SWI6 ---TTCTGAACATACTAAAGGAACAGAGAACAATATAAAAAATGAGAACACCACAAATAG 407

Smik_SWI6 CGATCCTGGCCATTCAAAAGAAAGAGAAAACAACATCAAAAGTGAAAACTCTACCAACAG 410

Scas_SWI6 ------------------------AAGGAATAATATGAAACGTGTGAAT-----GGATTG 375

* ** ** * *** ** ** *

Scer_SWI6 TATCAGCTCAAAGAGGATTAATAATTTACAAGATATGAGCCTGGATTCTGATGCACACAG 467

Sbay_SWI6 TGCCAGTTCTAAAAGGATCAATAATTTACAAGATATGGATGTAAATTCTGATGATCATAG 467

Smik_SWI6 TATCAACTCTAAGAGGGTTAACGATTTACAAAATGTTGATGCAGATTCAGATACTCATAG 470

Scas_SWI6 ------------GAGGA------ATTT----GGGATGAGTTTGGATTCTCACAGA---GG 410

*** **** * **** * *

Scer_SWI6 AGAATTAGGCTCTCCTTTAAAAAAACTAAAAATAGATACCTCTGTAATAGATGCTGAGAG 527

Sbay_SWI6 AGAATTGGGCTCTCCTTTGAAAAAGCTGAAAATCGATACTTCCGCGATAGAGTCAGAAAG 527

Smik_SWI6 AGAACTAGGCTCTCCCTTGAAGAAGTTGAAAATTGACACCTCTGCGATAGATCCGGAAAA 530

Scas_SWI6 AGAACTAGGATCACCTTTGAAGAAATTGAAGATGGACA---------------------- 448

**** * ** ** ** ** ** ** * ** ** ** *

Scer_SWI6 TGACTCCACTCCGAATACTGCCAGAGGCAAGCCTAACGATGATATTAATAAGGGCCCTAG 587

Sbay_SWI6 TGGCCCGACTTCAACTGCTGCCACAGTTGACACCAACAATGACGATAAGAGCAACGGCAG 587

Smik_SWI6 TGATTCAACTCCGAATACTGCCAGAGCCAAGACCAACAATAAAAATAAAGGCTCCGCCAA 590

Scas_SWI6 ----------------ACGACAACAG-TAATAAAAATAATAACAGTAATA-CAAAACCA- 489

* * * ** * ** ** * *** *

Scer_SWI6 CG---GCGACAATGAAAATAATGGCACTGATGACAATGACAGAACCGCTGGACCTATCAT 644

Sbay_SWI6 CGCCAGCAGCGACAACAACAATGACATAAATAACAAGGAGAGGACTGCTGAACCTATTGT 647

Smik_SWI6 TG---TTAGCGAGAGTAGCGACAACACTGATAAAAACGGAAGCACGGCCAAGCCAATAAT 647

Scas_SWI6 ------CAACAAAATAAGCTGCAACAGCGACAGGAA-GAGATCAATGACAGACCCATTAT 542

* * * ** * ** * * * * ** ** *

Scer_SWI6 AACATTCACTCATGACCTAACT---TCTGACTTTTTAAGCAGTCCACTGAAAATCATGAA 701

Sbay_SWI6 TACATTCACTCATGACCTAACT---TCTGAATTCTTGAACAATCCATTGAAAATTATGAA 704

Smik_SWI6 TACGTTCACTCATGACCTTACT---TCTGAATTTTTGAACACTCCATTGAAAATTATGAA 704

Scas_SWI6 TACTTTTGACCACGACTTACGTGACTCGACCATTACGAACTTGCCGTTGAAGATGAACCA 602

** ** ** *** * * ** * * * ** **** ** * *

Scer_SWI6 AGCACTACCTTCTCCAGTTGTAAATGATAATGAACAGAAGATGAAACTAGAGGCATTCTT 761

Sbay_SWI6 GACATCACCTTCTCCAATTGTAAACGATAATGAACAAAAGATGAAACTGGAGGCATTTCT 764

Smik_SWI6 AACATTGCCTTCTCCGATTGTAAACGATAATGAACAGAAGATGAAACTGGAGGCATTTTT 764

Scas_SWI6 CGCTTTGCAACTATCGTCTGAGATAGATAATGACGAAAGATTAAAATTGGAGAATTTCCT 662

* * * ** * ******** * * * *** * *** ** *

Scer_SWI6 ACAACGGTTGTTATTTCCAGAAATTCAAGAAATGCCTACATCCCTTAATAATGACAGCAG 821

Sbay_SWI6 CCAACGGTTGTTATTCCCAGAGATCCAAGAAATGCCCGCGTCTTTAAACAATGAAAGTAC 824

Smik_SWI6 GCAACGTTTGTTATTCCCGGAAATTCAAGAAGTGCGCACATCCCTGAATAATGACAATAA 824

Scas_SWI6 ACAAAGGTTATTATTACCCAATATTCAAAATA-ACTCAGATTCCTC--TAATGCTTATGA 719

*** * ** ***** ** * ** *** * * * * ****

Scer_SWI6 TAATAGAAATTCAGAAGGGGGGAGCTCAAACCAACAACAACAGCACGTATCATTTGATAG 881

Sbay_SWI6 TACTAGAAATTTGCAACAGGGAAGCTCAAGCCAACAGCAGCAGCATGTTTCATTTGACAG 884

Smik_SWI6 TACCAGAAATTTGGAGGAGGAGAACTCGAACCAAAAACAACAGCACGTGTCGTTTGATAC 884

Scas_SWI6 TAATGGA------------------------------------TCTGTATCATTTAATTC 743

** ** ** ** *** *

Scer_SWI6 CCTTTTGCAAGAGGTAAACGACGCTTTTCCTAATACTCAATTAAATCTTAATATTCCTGT 941

Sbay_SWI6 TGTTTTCCAAGAGGTCAATGACGCTTTTCCTCACACTCAATTAAATCTCAATATTCCCGT 944

Smik_SWI6 TGTTTTTCAAGAGGTTAACAACGCCTTTCCTAACACTCAATTAAATCTTAATATTCCCGT 944

Scas_SWI6 CTTGATGCATGAAATGGATGCTACTTTCCCACACACGTCATTAAATCTAAATATCCCAAT 803

* * ** ** * * * ** ** * ** ********* ***** ** *

Scer_SWI6 AGATGAGCATGGAAACACACCATTACATTGGCTGACTTCAATAGCAAACCTGGAATTAGT 1001

Sbay_SWI6 TGATGAACATGGAAATACTCCACTGCATTGGTTAACTTCGATAGCGAACCTAGAACTAGT 1004

Smik_SWI6 CGATGAACATGGAAACACACCCTTGCATTGGCTGACTTCAATAGCTAATCTCGAATTAGT 1004

Scas_SWI6 TGATGAATATGGGAATACCCCCCTACATTGGTTAACCTCTACGGCAAACATTGACTTAGT 863

***** **** ** ** ** * ****** * ** ** * ** ** * ** ****

Scer_SWI6 GAAACACCTGGTTAAGCATGGTTCAAACAGATTATATGGTGATAATATGGGGGAGTCATG 1061

Sbay_SWI6 GAAAAATTTAGTGAAGCATGGCTCAAACAGATTATACGGTGATAATATGGGAGAGTCGTG 1064

Smik_SWI6 GAAAAACCTGGTTAAGCATGGTTCAAACAGATTATATGGGGATAATACTGGAGAGTCGTG 1064

Scas_SWI6 GAAAGAAATGGTGAAAAATGGTGCTAATAGACTATTGGGAGACAATTCAGGTGAATCTGC 923

**** * * ** ** **** * ** *** *** ** ** *** ** ** **

Scer_SWI6 CCTAGTGAAAGCTGTCAAATCAGTAAATAATTACGACTCTGGTACTTTTGAGGCACTTCT 1121

Sbay_SWI6 TCTAGTGAAAGCTGTCAAATCAGTCAACAATTATGATTCTGGGACTTTTGAGGCACTCTT 1124

Smik_SWI6 TCTAGTGAAAGCTGTCAAATCAGTTAACAACTACGATTCAGGAACTTTTGAGGCACTCCT 1124

Scas_SWI6 TTTGGTGAAAGCTACCAAGACTGTAAACAACTACGATTCAGGGACATTTGAGGAACTCCT 983

* ********* *** * ** ** ** ** ** ** ** ** ******* *** *

Scer_SWI6 AGATTATTTATATCCATGTTTAATTTTGGAAGACTCAATGAATAGAACAATTTTGCACCA 1181

Sbay_SWI6 AGATTATCTATATCCATGTCTAATCTTGGAAGATTCAATGAGTAGAACAATTTTGCATCA 1184

Smik_SWI6 AGATTATCTATATCCATGCTTAATCTTGGAGGATTCAATGAATAGAACAATTCTGCATCA 1184

Scas_SWI6 AGACTATTTATATCCCTGTCTAATCTTATTAGATGCCATGGATAGAACTATATTACATCA 1043

*** *** ******* ** **** ** ** * *** ****** ** * ** **

Scer_SWI6 TATCATTATTACGTCTGGTATGACTGGCTGTTCAGCAGCTGCGAAATATTATTTAGATAT 1241

Sbay_SWI6 TATCATCATTACATCTGGTATGGCCGGTTGTTCGTCTGCTGCTAAATATTACTTAGATAT 1244

Smik_SWI6 TATCATTATTACGTCTGGTATGACTGGTTGTTCCTCTGCTGCCAAGTACTATTTAGATAT 1244

Scas_SWI6 TATTGTGATTACATCGGGGATGAAAGATCATGCTCTTGTAGCCAAATATTATCTAGATAT 1103

*** * ***** ** ** *** * * * * ** ** ** ** *******

Scer_SWI6 TTTAATGGGATGGATTGTCAAGAAACAAAATAGACCCATTCAAAGTGGTACTAACGAAAA 1301

Sbay_SWI6 TTTGATGGGATGGATCGTTAAGAAACAAAACAGACCCATTCAAAGTGGTAATAATGAC-- 1302

Smik_SWI6 TTTGATGGGATGGATTGTTAAAAAACAAAATAGGCCCATTCAAATTGGTAGTAGTGGTAG 1304

Scas_SWI6 ACTAATGGGATGGATAGTGAAAAAGCAACCAAGACCTATCCATGGTGTGGGAAATGGA-- 1161

* *********** ** ** ** *** ** ** ** ** ** * *

Scer_SWI6 AGAAAGCAAACCGAATGACAAAAATGGGGAAAGAAAGGACTCTATACTAGAAAATTTGGA 1361

Sbay_SWI6 -GGGAATAAGAGCGATAACGAAAAAGAAGAAAAGAGAGATTCAATTCTAGAAAATCTGGA 1361

Smik_SWI6 AGGAGGTAAAGTAGATGATGAAAACGATGAGAAACGGGATTCAATACTAGAAAATCTAGA 1364

Scas_SWI6 ----------------------------------------CCAGTGATTGATAGTTTAGA 1181

* * * ** * * * **

Scer_SWI6 CCTGAAATGGATAATAGCAAACATGCTCAATGCGCAGGACTCTAATGGCGATACTTGCTT 1421

Sbay_SWI6 TTTGAAATGGATAATAACTAACATGCTGAATGCCCAAGACTCTAACGGTGATACTTGTCT 1421

Smik_SWI6 CCTGAAATGGATAATAACAAATATGCTCAATGCACAGGACTCTAATGGCGATACTTGCTT 1424

Scas_SWI6 TTTGAAATGGATCTTAACAAATATGTTGAACGCTCAAGATGTTAATGGTGACACTTGTCT 1241

********** ** * ** *** * ** ** ** ** *** ** ** ***** *

Scer_SWI6 GAACATTGCAGCAAGATTGGGAAACATTTCAATAGTAGACGCTTTATTAGATTACGGTGC 1481

Sbay_SWI6 GAATATCGCGGCAAGACTGGGAAATATATCCATAGTAGATGCTTTATTAGATTACGGTGC 1481

Smik_SWI6 GAACATTGCAGCAAGATTGGGAAACATTTCAATAGTAGACGCCCTATTAGATTACGGTGC 1484

Scas_SWI6 AAATATTGCTGCTCGATTAGGTAATGTCGGGATTGTAGATGCATTATTGGAATATGGTGC 1301

** ** ** ** ** * ** ** * ** ***** ** **** ** ** *****

Scer_SWI6 TGACCCATTTATTGCAAACAAATCAGGGTTGAGACCCGTGGATTTTGGGGCAGGTACTTC 1541

Sbay_SWI6 AGACCCATTTATTGCAAACAAGTCCGGTCTAAGACCTGTGGATTTTGGGGCAGGTACTTC 1541

Smik_SWI6 AGACCCATTTATTGCTAATAAGTCAGGTTTAAGACCCGTAGATTTTGGGGCTGGTACTTC 1544

Scas_SWI6 AGATCCTTATATTGCTAACAAGTCTGGGTTAAGACCTTTAGACTTTGGAGCTGGTACTTC 1361

** ** * ****** ** ** ** ** * ***** * ** ***** ** ********

Scer_SWI6 AAAATTACAAAATACGAACGGCGGTGACGAAAACTCGAAGATGGT--CTCTAAGGGTGAT 1599

Sbay_SWI6 AAAATTACAAAATAACAACAGCGATGACAAAAACTCTACTATGAT--CTCTAAGGAAGAT 1599

Smik_SWI6 AAAATTACAAAATAGTAACGACGGTGACGAAAACTCGAAGTTGAC--CACTAAGGGAGAT 1602

Scas_SWI6 GAAGTTTCAAAC--GCATGAACTAGAGCTAAATCTAGATCATGACGGTGCTAATAAACAT 1419

** ** **** * * * *** ** * ** **** **

Scer_SWI6 TACGACGGTCAAAAAAATG--------GA------AAGGCCAAAAAAAT-----AAGGTC 1640

Sbay_SWI6 TCTGATGGTACAAACAGCG--------GA------AGCAACAAAAAAAT-----AAGGGC 1640

Smik_SWI6 TCTGAAGATCAACTTAACG--------GA------AACCCCCCAAAAAT-----AAGGCC 1643

Scas_SWI6 CATGGAGGTGATGATGACGCCATTTTAGATCATCCAACATCCTCAGATTTATTGGAGGAC 1479

* * * * ** * * * * * *** *

Scer_SWI6 -------TCAACTG-TTGAAAAACCCACCTGAAACAACTTCGTTAATTAATGATGTCCAA 1692

Sbay_SWI6 -------TCAACTA-CTGAAAAATCCCCCTGAAACGACCTCGTTAATTAATGAAGTTCAA 1692

Smik_SWI6 -------TCAAATA-TTGAAAAATCCCCCTGAGACGACTTCCCTAATTAATGATGTTCAA 1695

Scas_SWI6 GGTATTGTCAGTTCTCCAAAGAATAAACCAGATACGAAAGCCTTGGTTAGTGAATTGCAA 1539

*** * ** ** ** ** ** * * * *** *** * ***

Scer_SWI6 AATTTACTGAATTCAATCTCGAAAGATTATGAGAATGAAACAGTGCAATATAATGAGAAA 1752

Sbay_SWI6 AATTTACTAAACTCTATCTCAAAAGACTATGAGTCAGAGACAATACAATATAACGAAAAA 1752

Smik_SWI6 AGTTTACTGAACTCCATTTCGAAAGATTACGAAAGCGAGACAATGCAATACGATGAGAAG 1755

Scas_SWI6 ACATTACTTGACGCTGTCTCAAAGGATTACGATATAGAGATGTCAACGAATAAGGAAAAG 1599

* ***** * * * ** ** ** ** ** ** * * * ** **

Scer_SWI6 TTAGAAAAACTACACAAGGAATTGAACGAACAGCGAGAAGAATTAGCTAATTCTAGAGAA 1812

Sbay_SWI6 TTAGAAAATCTCCATAAAGAATTGAATGAACAGCGAAATAAATTGGCTAGTTCGAGAGAT 1812

Smik_SWI6 CTAAAAATTCTTCATAAGGAACTAAACAAACAGCGAGAAGAGTTAGCTAATTCAAGAGAC 1815

Scas_SWI6 CTTGAGAAACTGAAAGAAAAATTAAACACAAAACGTCAAGAACTTTCTACTTCAAGAGAT 1659

* * * ** * * ** * ** * * ** * * * *** *** *****

Scer_SWI6 CAACTAGCAAATGTGAAGCAATTGAAAGATGAATACTCACTAATGCAAGAGCAATTGACC 1872

Sbay_SWI6 CAATTAGCAAGTGTGAAGCAATTAAAAGACGAATACTCATTAATGCAAGAGCAATTGACT 1872

Smik_SWI6 CAGTTAGCAAATGTCAAACAGTTGAAGGACGAATATTCATTAATGCAAGAACAATTGACC 1875

Scas_SWI6 CGGTTGACGCAAACAAAGCAATTAAGAGATGAATATCAACTATTGAAGGAGCAGGTAAAT 1719

* * * ** ** ** * ** ***** * ** ** * ** ** * *

Scer_SWI6 AATTTAAAAGCAGGAATTGAAGAAGAAGAGGAAAGTTTTAGGGAAGAGAGCAAAAAGCTA 1932

Sbay_SWI6 AATTTGAAGTCTGGCATCGAAGAAGAAGAAGCAAATTTTAGGGAGGAAAGCAAAAAGCTG 1932

Smik_SWI6 AATCTGAAAGCAGGCATAGAAGAAGAAGAGGCAAGTTTTAGAGAAGAGAGCAAAAAGTTG 1935

Scas_SWI6 AATATTAAAAAAGGTATTGCAGAGCAGGAGGAGAGCTTCCAACACGAAAGTGAGAAGTTA 1779

*** * ** ** ** * *** * ** * * ** * ** ** * *** *

Scer_SWI6 GGAATAATTGCAGATGAAAGTTCAGGTATTGATTGGGACTCTAGCGAATATGATGCAGAT 1992

Sbay_SWI6 GGAATTATTACAGACGAAAGTTCGGGTATTGATTGGGAATCCAGTGAATATGATGCAGAT 1992

Smik_SWI6 GGAATTGTAGCGGATGAAAGTTCAGCTATTGACTGGGACTCCAGTGAATATGATGCAGAT 1995

Scas_SWI6 GGTATTTCGGCCGAAGATTCTGTTGGTATTGATTGGGATTCCAGTGAATTTGATGCAGAT 1839

** ** * ** ** * * ****** ***** ** ** **** **********

Scer_SWI6 GAACCCTTCAAAGTAGAGTTCCTTTCAGATTTCTTGGAGGATAAATTACAAAAGAATTAT 2052

Sbay_SWI6 GAGCCCTTCAAAGTGGAGCTCATTTCAGATTTCATGGAGGAAAAACTACAAACGGACTAT 2052

Smik_SWI6 GAGCCATTTAAAGTCGAGTTCATTTCAGATTTTTTGGAGGATAAGTTGCAAAGGGATTAT 2055

Scas_SWI6 GAGCCATTTAGAATTGAGTTCATATATGATCTGTTAGAGAACAGGTTAACTGATGTATAT 1899

** ** ** * * * *** ** * * *** * * *** * * * ***

Scer_SWI6 GAAGGCGATATTTCCAAACTATTAGAAGCGGAGTCGAAAGAGCAGATAATGGAACAGATA 2112

Sbay_SWI6 GACGGTGACATTTCCAAATTGCTAGAATCAGAGTCAAAAGAGCAAATAATGGAACAAATA 2112

Smik_SWI6 GAAGGTGATATTTCCAAACTACTAGAATCGGAGCCAAAAGACCAAATAATTGAACAAATA 2115

Scas_SWI6 AATGGTGATATTGAAAAATTATTGAAGGAAGAGAATGTAGATGAACTTGTCTCGAAAATA 1959

* ** ** *** *** * * * *** *** * * * * ***

Scer_SWI6 CGAAATCAACTTCCCGCAGAAAAAATCCAATCGATGCTTCCACCAACGGTCTTATTGAAG 2172

Sbay_SWI6 CGAAATCAAATGTCGATAGAAAAAATCGATTCCATGCTACCACCTACCGTTTTATTAAAA 2172

Smik_SWI6 CGAAATCAAATGCCAGTAGAAAAAATCAATTCGATGCTTCCACCAACTGTTCTACTAAAA 2175

Scas_SWI6 CGAAAT--GGTTTTGAT-GAACGATTAAATTCCATATTACCTCCCAAATTCCTATTACAG 2016

****** * *** * * * ** ** * ** ** * * ** * *

Scer_SWI6 GCAAGAATAAATGCATACAAGCGGAATGATAAACACCTAACCAATGTATTGGATACAATC 2232

Sbay_SWI6 GCAAGAATAAACGCATACAAGAGAAACGATAAACACTTAAGCAATGTCTTGCACACAATT 2232

Smik_SWI6 GCAAGAATAAATGCATACAAAAGGAACGATAAACACCTAAATAATGTTTTACACACAATC 2235

Scas_SWI6 GCAAGAGTCAATGCGTATAAGAGAAATGATGATCAATTACAAAAAATATATGATGGGATA 2076

****** * ** ** ** ** * ** *** * ** ** ** * * * **

Scer_SWI6 AGCACCAAACAATCAGAATTAGAGAATAAATTTAGAAGAGTGTTATCTTTATGTTTGAAA 2292

Sbay_SWI6 AGTACCAAGCAATCAGAATTAGAAAATAAATTTAGAAGAGTGTTATCCCTATGTTTAAAG 2292

Smik_SWI6 AGTACCAAACAATCGGAATTGGAAAATAAGTTCAGGAGAGTGTTGTCTTTATGTTTGAAG 2295

Scas_SWI6 AAACAAAAACAAGATGATTTGGAAAGTAAATTCAGAAGAGTACTATCTTTATGTTTAAAG 2136

* ** *** ** ** ** * *** ** ** ***** * ** ******* **

Scer_SWI6 ATCGACGAAAATAAAGTTGACAATATGCTTGATGGTTTGTTACAGGCCATATCTTCTGAA 2352

Sbay_SWI6 ATTGATGAAAACAAAGTCGACGATATGCTTGACGGTTTGTTGCAAGCCATATCATCCGAA 2352

Smik_SWI6 ATTGACGAAAACAAGGTCGACGATATGCTTGATGGATTGTTACAGGCCATATCTTCCGAA 2355

Scas_SWI6 ATTGATGAAGACAAAGTGGATGGGATGCTTGATGGCTTATTACAAGCAATTTCATTTGAA 2196

** ** *** * ** ** ** ******** ** ** ** ** ** ** ** * ***

Scer_SWI6 GACCCACAAGACATTGACACTGACGAAATGCAAGATTTTTTAAAAAAGCATGCTTCATGA 2412

Sbay_SWI6 GACCCGCAAGATATCGATACCGATGAAATGCAAGATTTCTTAAAAAAGCATGCTTCGTGA 2412

Smik_SWI6 GACCCGCAGGATATCGACACCGACGAAATGCAGGATTTTCTTAGAAAGCATGCTTCGTGA 2415

Scas_SWI6 GATCCCCATGACATTGACACGGACGAAATGCAAGATTTCTTGAAAAAACATGCAGTTTAA 2256

** ** ** ** ** ** ** ** ******** ***** * * *** ***** * *

B


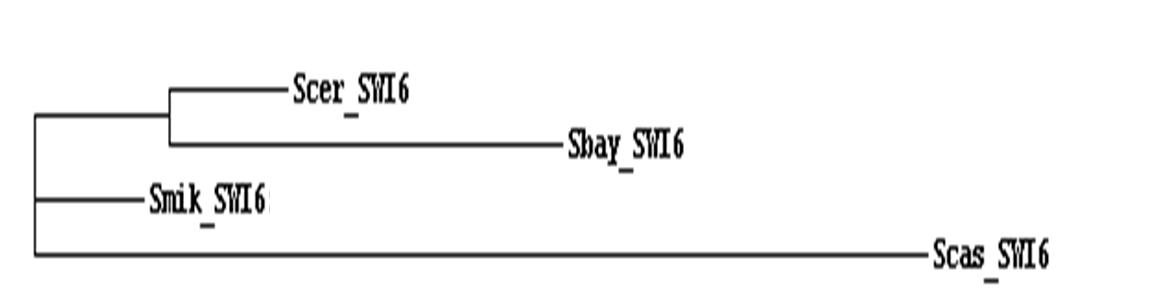

Supplement: Figure S25 — The sequence alignment and gene tree of SWI6 gene. Sequence alignments of SWI6 of S. cerevisiae, S. mikatae and S. uvarum (Panel A) and the relative gene tree (Panel B).The sensu lato species S. castelli was used as outgroup. (DOC) [file pgen.1003836.s025.doc]
