# Supplementary material for: Chimeric Protein Complexes in Hybrid Species Generate Novel Phenotypes
Source: PLoS Genet. 2013 Oct 3;9(10):e1003836. doi: 10.1371/journal.pgen.1003836 (PMC3789821; doi:10.1371/journal.pgen.1003836)
Supplement: Figure S26 — Growth curves for strains bearing the different types of TRP2/TRP3 protein complex in absence (A) and presence (B) of tryptophan. (DOC) [file pgen.1003836.s026.doc]

Figure S26

A

B
