# Supplementary material for: Chimeric Protein Complexes in Hybrid Species Generate Novel Phenotypes
Source: PLoS Genet. 2013 Oct 3;9(10):e1003836. doi: 10.1371/journal.pgen.1003836 (PMC3789821; doi:10.1371/journal.pgen.1003836)
Supplement: Figure S28 — Competition fitness assay between TRP2pSu/TRP3pSc strain and the reference strain in presence (red line) or absence (blue line) of tryptophan. Three biological replicas were tested (A, B and C). (DOC) [file pgen.1003836.s028.doc]

Figure S28

A

B

C
