# Supplementary material for: Chimeric Protein Complexes in Hybrid Species Generate Novel Phenotypes
Source: PLoS Genet. 2013 Oct 3;9(10):e1003836. doi: 10.1371/journal.pgen.1003836 (PMC3789821; doi:10.1371/journal.pgen.1003836)
Supplement: Figure S29 — Fitness of Sc/Su hybrids carrying a deletion of one of the member of the MBF complex. The construction of Sc/Su hybrids carrying different type of deletions of homologous member of the MBF complex is shown in Panel A. The growth of such strains in both YPD and YP-glycerol is shown in Panel B. Deletion of either Mbp1Su or Swi6Su (4 and 5) affect the growth of the hybrids when glycerol is present as sole carbon source. (DOC) [file pgen.1003836.s029.doc]

Figure S29
