# Supplementary material for: Chimeric Protein Complexes in Hybrid Species Generate Novel Phenotypes
Source: PLoS Genet. 2013 Oct 3;9(10):e1003836. doi: 10.1371/journal.pgen.1003836 (PMC3789821; doi:10.1371/journal.pgen.1003836)
Supplement: Figure S30 — Fitness of parental strains (Sc and Sm) and Sc/Sm hybrids carrying different combination of members of the MBF complex. The construction of Sc/Sm hybrids carrying different type of MBF complexes, either chimeric or uni-specific is shown in Panel A. The growth of such strains in both YPD and YP-glycerol is shown in Panel B. No difference in fitness is detected among hybrids carrying the different complexes (1–5) and the parental strains (Sc and Sm). (DOC) [file pgen.1003836.s030.doc]

Figure S30

**A**

**B**
