# Supplementary material for: Chimeric Protein Complexes in Hybrid Species Generate Novel Phenotypes
Source: PLoS Genet. 2013 Oct 3;9(10):e1003836. doi: 10.1371/journal.pgen.1003836 (PMC3789821; doi:10.1371/journal.pgen.1003836)
Supplement: Figure S31 — Evolutionary perspective of chimeric protein interaction in hybrids. In yeast hybrids, where two proteomes co-exist, there could be preferential formation of uni-parental protein complexes (A) or the potential to establish chimeric interactions (B). The ability or not to form fully functional chimeric complexes will have an impact on gene loss during genome evolution, and on the adaptability potential of the cells, since different types of complexes can confer diverse phenotypic traits to the hybrids (represented by the different colours of the yeast cell wall), upon which natural selection may act. (DOC) [file pgen.1003836.s031.doc]

Figure S31

**A**

**B**
