# Supplementary material for: Chimeric Protein Complexes in Hybrid Species Generate Novel Phenotypes
Source: PLoS Genet. 2013 Oct 3;9(10):e1003836. doi: 10.1371/journal.pgen.1003836 (PMC3789821; doi:10.1371/journal.pgen.1003836)
Supplement: Table S1 — Protein complexes selected for analysis. The complexes encompass different biological functions and orthologs members have sufficient divergence in terms of tryptic digest profile. (DOCX) [file pgen.1003836.s032.docx]

**Table S1**

| **Protein complexes** | **Components MIPS** | **Systematic deletion** | **Sc-Sm aa**  **identity** | **Sc-Sm tryptic digest identity** | **Sc-Su aa**  **identity** | **Sc-Su tryptic digest identity** | **Biological role** |
| --- | --- | --- | --- | --- | --- | --- | --- |
| **Sec 62/63** | ***SEC62***  ***SEC63***  ***SEC66***  ***SEC72*** | **Lethal**  **Viable**  **Viable**  **Viable** | **91 %**  **90 %**  **96 %**  **95 %** | **70 %**  **35 %**  **60%**  **80%** | **89%**  **88%**  **93%**  **91%** | **70%**  **30%**  **50%**  **60%** | **Translocation to ER** |
| **MBF complex** | ***MBP1***  ***SWI6*** | **Viable**  **Viable** | **90%**  **85%** | **55%**  **17%** | **85%**  **83%** | **40%**  **23%** | **Transcription factors, G1 to S phase transition** |
| **Farnesyltransferase** | ***RAM1***  ***RAM2*** | **Viable**  **Lethal** | **84%**  **86%** | **24%**  **55%** | **84%**  **86%** | **24%**  **55%** | **Protein farnesylation** |
| **Ku complex** | ***YKU70***  ***YKU80*** | **Viable**  **Viable** | **83%**  **82%** | **21%**  **19%** | **80%**  **81%** | **18%**  **13%** | **DNA DSB repair**  **NHEJ** |
| **Anthranile synthase** | ***TRP2***  ***TRP3*** | **Viable**  **Viable** | **95%**  **93%** | **60%**  **38%** | **94%**  **91%** | **50%**  **28%** | **Tryptophan biosynthesis** |
| **CTK** | ***CTK1***  ***CTK2***  ***CTK3*** | **Viable**  **Viable**  **Viable** | **87%**  **91%**  **88%** | **33%**  **48%**  **40%** | **83%**  **87%**  **83%** | **30%**  **44%**  **37%** | **Pre-mRNA processing** |
