# Supplementary material for: Chimeric Protein Complexes in Hybrid Species Generate Novel Phenotypes
Source: PLoS Genet. 2013 Oct 3;9(10):e1003836. doi: 10.1371/journal.pgen.1003836 (PMC3789821; doi:10.1371/journal.pgen.1003836)
Supplement: Table S2 — Summary table of the biochemical and MS data for the Sec 62–63 protein complex in the Sc/Sm hybrid. (DOCX) [file pgen.1003836.s033.docx]

**Table S2**

| Protein complex  Member | Molecular weight *Sc* (kDa) | Isoelectic point *Sc* (pI) | Molecular weight *Sm* (kDa) | Isoelectic point *Sm* (pI) | *Sc* peptides | *Sm* peptides | *Sc/Sm* shared peptides |
| --- | --- | --- | --- | --- | --- | --- | --- |
| Sec62p- TAP | 31,3 | 10.5 | 31,9 | 9.69 | 2 * | none | 7 |
| Sec63p | 75,3 | 4.97 | 75,5 | 4.97 | 13 | 9 * | 10 |
| Sec66p | 24,2 | 7.27 | 24,2 | 5.89 | 1 * | none | 8 |
| Sec72p | 21,6 | 5.48 | 21,6 | 5.33 | 1 * | 2 * | 4 |

* see Figure S9, S13, S14 and S15 for spectra
