# Supplementary material for: Chimeric Protein Complexes in Hybrid Species Generate Novel Phenotypes
Source: PLoS Genet. 2013 Oct 3;9(10):e1003836. doi: 10.1371/journal.pgen.1003836 (PMC3789821; doi:10.1371/journal.pgen.1003836)
Supplement: Table S3 — Summary table of biochemical and MS data for the Sec 62–63 protein complex in the Sc/Su hybrid. (DOCX) [file pgen.1003836.s034.docx]

**Table S3**

| Protein complex  Member | Molecular weight *Sc* (kDa) | Isoelectic point *Sc* (pI) | Molecular weight *Su* (kDa) | Isoelectic point *Su* (pI) | *Sc* peptides | *Su* peptides | *Sc/Su* shared peptides |
| --- | --- | --- | --- | --- | --- | --- | --- |
| Sec62p- TAP | 31,3 | 10.5 | 34,6 | 9.68 | 2 * | none | 6 |
| Sec63p | 75,3 | 4.97 | 75,3 | 5.00 | 15 | 5 * | 6 |
| Sec66p | 24,2 | 7.27 | 24,1 | 5.60 | 2 * | none | 7 |
| **Sec72p** | **21,6** | **5.48** | **21,5** | **5.19** | **2 *** | **4** | **3** |

* see Figure S10, S11, S14 and S16 for spectra
