# Supplementary material for: Chimeric Protein Complexes in Hybrid Species Generate Novel Phenotypes
Source: PLoS Genet. 2013 Oct 3;9(10):e1003836. doi: 10.1371/journal.pgen.1003836 (PMC3789821; doi:10.1371/journal.pgen.1003836)
Supplement: Table S4 — Summary table of biochemical and MS data for the TRP2/TRP3 complex in the Sc/Sm hybrid. (DOCX) [file pgen.1003836.s035.docx]

**Table S4**

| Protein complex member | Molecular weight *Sc* (kDa) | Isoelectic point *Sc* (pI) | Molecular weight *Sm* (kDa) | Isoelectic point *Sm* (pI) | *Sc* peptides | *Sm* peptides | ***Sc/Sm* shared peptides** |
| --- | --- | --- | --- | --- | --- | --- | --- |
| Trp2 -TAP | 56,7 | 5.7 | 56,6 | 5.81 | 6 | none | 15 |
| Trp3p | 53,4 | 6.9 | 53,3 | 6.16 | 5 | 2 * | 8 |

*see Figure S18 for spectra
