# Supplementary material for: Chimeric Protein Complexes in Hybrid Species Generate Novel Phenotypes
Source: PLoS Genet. 2013 Oct 3;9(10):e1003836. doi: 10.1371/journal.pgen.1003836 (PMC3789821; doi:10.1371/journal.pgen.1003836)
Supplement: Table S5 — Summary table of biochemical and MS data for the TRP complex in the Sc/Su hybrid. (DOCX) [file pgen.1003836.s036.docx]

**Table S5: Summary table of biochemical and MS data for the TRP complex in the *Sc/Su* hybrid**

| Protein complex member | Molecular weight *Sc* (kDa) | Isoelectic point *Sc* (pI) | Molecular weight *Su* (kDa) | Isoelectic point *Su* (pI) | *Sc* peptides | *Su* peptides | Sc/Su shared peptides |
| --- | --- | --- | --- | --- | --- | --- | --- |
| Trp2p- TAP | 56,7 | 5.7 | 56,6 | 5.62 | 11 | none | 11 |
| Trp3p | 53,4 | 6.9 | 53,5 | 5.64 | 10 | 5 | 5 |
