# Supplementary material for: Chimeric Protein Complexes in Hybrid Species Generate Novel Phenotypes
Source: PLoS Genet. 2013 Oct 3;9(10):e1003836. doi: 10.1371/journal.pgen.1003836 (PMC3789821; doi:10.1371/journal.pgen.1003836)
Supplement: Table S6 — Summary table of biochemical and MS data for the CTK complex in the Sc/Sm hybrid. (DOCX) [file pgen.1003836.s037.docx]

**Table S6: Summary table of biochemical and MS data for the CTK complex in the *Sc/Sm* hybrid**

| Protein complex member | Molecular weight *Sc* (kDa) | Isoelectic point *Sc* (pI) | Molecular weight *Sm* (kDa) | Isoelectic point *Sm* (pI) | *Sc* peptides | *Sm* peptides | ***Sc/Sm* shared peptides** |
| --- | --- | --- | --- | --- | --- | --- | --- |
| Ctk1p TAP | 60,5 | 10.3 | 62,2 | 9.63 | 6 | none | 9 |
| Ctk2p | 37,9 | 8.05 | 37,8 | 8.03 | 3 | 3 | 2 |
| Ctk3p | 34,8 | 6.29 | 34,7 | 5.49 | 1 * | 1 * | 2 |

* see Figure S19 and S20 for spectra
