# Supplementary material for: Chimeric Protein Complexes in Hybrid Species Generate Novel Phenotypes
Source: PLoS Genet. 2013 Oct 3;9(10):e1003836. doi: 10.1371/journal.pgen.1003836 (PMC3789821; doi:10.1371/journal.pgen.1003836)
Supplement: Table S7 — Summary table of biochemical and MS data for the CTK complex in the Sc/Su hybrid. (DOCX) [file pgen.1003836.s038.docx]

**Table S7**

| Protein complex member | Molecular weight *Sc* (kDa) | Isoelectic point *Sc* (pI) | Molecular weight *Su* (kDa) | Isoelectic point *Su* (pI) | *Sc* peptides | *Su* peptides | ***Sc/Su* shared peptides** |
| --- | --- | --- | --- | --- | --- | --- | --- |
| Ctk1p TAP | 60,5 | 10.3 | 60,4 | 9.61 | 7 | none | 9 |
| Ctk2p | 37,9 | 8.05 | 41,4 | 7.10 | none | none | none |
| Ctk3p | 34,8 | 6.29 | 34,5 | 5.86 | none | none | 2 |
