# Supplementary material for: Chimeric Protein Complexes in Hybrid Species Generate Novel Phenotypes
Source: PLoS Genet. 2013 Oct 3;9(10):e1003836. doi: 10.1371/journal.pgen.1003836 (PMC3789821; doi:10.1371/journal.pgen.1003836)
Supplement: Table S8 — Summary table of biochemical and MS data for the MBF protein complex in the Sc/Sm hybrid. (DOCX) [file pgen.1003836.s039.docx]

**Table S8**

| Protein complex  member | Molecular weight *Sc* (kDa) | Isoelectic point *Sc* (pI) | Molecular weight *Sm* (kDa) | Isoelectic point *Sm* (pI) | *Sc* peptides | *Sm* peptides | ***Sc/Sm* shared peptides** |
| --- | --- | --- | --- | --- | --- | --- | --- |
| Mbp1p TAP | 93,9 | 10.8 | 94,3 | 9.22 | 7 | none | **14** |
| Swi6p | 90,5 | 4.58 | 90,9 | 4.72 | 19 | none | 8 |
