# Supplementary material for: Chimeric Protein Complexes in Hybrid Species Generate Novel Phenotypes
Source: PLoS Genet. 2013 Oct 3;9(10):e1003836. doi: 10.1371/journal.pgen.1003836 (PMC3789821; doi:10.1371/journal.pgen.1003836)
Supplement: Table S9 — Summary table of biochemical and MS data for the MBF protein complex in the Sc/Su hybrid. (DOCX) [file pgen.1003836.s040.docx]

**Table S9**

| Protein complex member | Molecular weight *Sc* (kDa) | Isoelectic point *Sc* (pI) | Molecular weight *Su* (kDa) | Isoelectic point *Su* (pI) | *Sc* peptides | *Su* peptides | *Sc/Su* shared peptides |
| --- | --- | --- | --- | --- | --- | --- | --- |
| Mbp1p TAP | 93,9 | 10.8 | 94,7 | 9.15 | 9 | none | 11 |
| Swi6p | 90,5 | 4.58 | 90,7 | 4.63 | 10 | 7 | 5 |

* see Figure S21 for examples of *S. uvarum* Swi6 spectra
