# Supplementary material for: Chimeric Protein Complexes in Hybrid Species Generate Novel Phenotypes
Source: PLoS Genet. 2013 Oct 3;9(10):e1003836. doi: 10.1371/journal.pgen.1003836 (PMC3789821; doi:10.1371/journal.pgen.1003836)
Supplement: Table S10 — Targeted MS of Sm Swi6p in Sc/Sm background. The table includes a selection of peptides specific to Swi6p that were used to determine whether the protein was present. Mass to charge ratios that are underlined and in bold were used to direct the mass spectrometer via an inclusion list. (DOCX) [file pgen.1003836.s041.docx]

**Table S10**

| **Sequence** | **Theoretical** | **[M+H]** | **[M+2H]** | **[M+3H]** |
| --- | --- | --- | --- | --- |
| ARINAYK | 834.47 | 835.48 | **418.24** | 279.16 |
| QSELENK | 846.41 | 847.42 | **424.21** | 283.14 |
| ELGSPLKK | 870.52 | 871.53 | **436.27** | 291.18 |
| RVLSLCLK | 930.57 | 931.58 | **466.29** | 311.2 |
| MALEEVVR | 945.5 | 946.5 | **473.76** | 316.17 |
| MKLEAFLQR | 1134.62 | 1135.63 | **568.32** | 379.22 |
| QSELENKFR | 1149.58 | 1150.59 | **575.8** | 384.2 |
| DSILENLDLK | 1158.61 | 1159.62 | **580.31** | 387.21 |
| VEFLSDFLEDK | 1340.65 | 1341.66 | **671.33** | 447.89 |
| VLSLCLKIDENK | 1373.76 | 1374.77 | **687.89** | 458.93 |
| SGLRPVDFGAGTSK | 1390.72 | 1391.73 | **696.37** | 464.58 |
| VLSLCLKELGSPLK | 1498.88 | 1499.89 | **750.45** | 500.63 |
| YYLDILMGWIVK | 1512.81 | 1513.81 | **757.41** | 505.28 |
| YLGPHNEIPLTLTR | 1622.88 | 1623.89 | **812.45** | 541.97 |
| DEYSLMQEQLTNLK | 1710.81 | 1711.82 | **856.41** | 571.28 |
| QLKDEYSLMQEQLTNLK | 2080.05 | 2081.06 | 1041.03 | **694.36** |
| MALEEVVRYLGPHNEIPLTLTR | 2550.36 | 2551.37 | 1276.19 | **851.13** |
| LGIIADESSGIDWDSSEYDADEPFK | 2758.21 | 2759.22 | 1380.11 | **920.41** |
| KLGIIADESSGIDWDSSEYDADEPFK | 2886.31 | 2887.32 | 1444.16 | **963.11** |
