# Supplementary material for: Chimeric Protein Complexes in Hybrid Species Generate Novel Phenotypes
Source: PLoS Genet. 2013 Oct 3;9(10):e1003836. doi: 10.1371/journal.pgen.1003836 (PMC3789821; doi:10.1371/journal.pgen.1003836)
Supplement: Table S11 — Summary table of biochemical and MS data for the RAM complex in the Sc/Sm hybrid. (DOCX) [file pgen.1003836.s042.docx]

**Table S11**

| Protein complex member | Molecular weight *Sc* (kDa) | Isoelectic point *Sc* (pI) | Molecular weight *Sm* (kDa) | Isoelectic point *Sm* (pI) | *Sc* peptides | *Sm* peptides | *Sc/Sm* shared peptides |
| --- | --- | --- | --- | --- | --- | --- | --- |
| Ram1p- TAP | 48,1 | 6.35 | 48,6 | 5.86 | 5 | None | 1 |
| Ram2p | 37,5 | 4.83 | 37,5 | 5.40 | 6 | None | 5 |
