# Supplementary material for: Chimeric Protein Complexes in Hybrid Species Generate Novel Phenotypes
Source: PLoS Genet. 2013 Oct 3;9(10):e1003836. doi: 10.1371/journal.pgen.1003836 (PMC3789821; doi:10.1371/journal.pgen.1003836)
Supplement: Table S13 — Summary table of biochemical and MS data for the KU complex in the Sc/Sm hybrid. (DOCX) [file pgen.1003836.s044.docx]

**Table S13: Summary table of biochemical and MS data for the KU complex in the *Sc/ Sm* hybrid.**

| Protein complex member | Molecular weight *Sc* (kDa) | Isoelectic point *Sc* (pI) | Molecular weight *Sm* (kDa) | Isoelectic point *Sm* (pI) | *Sc* peptides | *Sm* peptides | *Sc/Sm* shared peptides |
| --- | --- | --- | --- | --- | --- | --- | --- |
| Yku70p TAP | 70,6 | 6.31 | 72,4 | 6.25 | 6 | none | 1 |
| Yku80p | 71,2 | 4.99 | 71,1 | 5.33 | 5 | none | 2 |
