# Supplementary material for: Chimeric Protein Complexes in Hybrid Species Generate Novel Phenotypes
Source: PLoS Genet. 2013 Oct 3;9(10):e1003836. doi: 10.1371/journal.pgen.1003836 (PMC3789821; doi:10.1371/journal.pgen.1003836)
Supplement: Table S14 — Summary table of biochemical and MS data for the KU complex in the Sc/Su hybrid. (DOCX) [file pgen.1003836.s045.docx]

**Table S14**

| Protein complex member | Molecular weight *Sc* (kDa) | Isoelectic point *Sc* (pI) | Molecular weight *Su* (kDa) | Isoelectic point *Su* (pI) | *Sc* peptides | *Su* peptides | *Sc/Su* shared peptides |
| --- | --- | --- | --- | --- | --- | --- | --- |
| Yku70p TAP | 70,6 | 6.31 | 70,2 | 6.22 | 5 | none | None |
| Yku80p | 71,2 | 4.99 | 70,7 | 5.07 | 3 | none | 2 |
