# Supplementary material for: Chimeric Protein Complexes in Hybrid Species Generate Novel Phenotypes
Source: PLoS Genet. 2013 Oct 3;9(10):e1003836. doi: 10.1371/journal.pgen.1003836 (PMC3789821; doi:10.1371/journal.pgen.1003836)
Supplement: Table S15 — List of primers for the specific amplification of the 16 S. cerevisiae chromosomes. (DOCX) [file pgen.1003836.s046.docx]

| **Chromosome** | **Forward primer sequence 5’-3’** | **Reverse primer sequence 5’-3’** |
| --- | --- | --- |
| I | GTTGGCTAGTTTCGCATTC | CCTTCATTGTTCTTGTCTGATG |
| II | TGCGGTAGCATTTCCATC | GGTTAGTGGCCAGATTGAGA |
| III | ACCTTTCCCTGATGTACGTC | CTTCACATCAAACTTGTGCC |
| IV | GACAAGCAACACAGTGTTTTAG | AGCTACTGTGACGACCAGG |
| V | ACGACATTCCATCTCATCG | GAGAGCGTGAGAAAATACTGC |
| VI | TTAGCAACTCTATGTACAACCG | TGATAAGTTTGATTGCGTCC |
| VII | GAATCTCTTGGTAGACTTGACC | GAAATGAACCTGCCAGAAG |
| VIII | AGAAACTGTGAGGTTCCCTAAG | AATACTGGTTCAAAACGTGG |
| IX | ATAATGGCAATTGTGGAATG | TTACCAGGCGTACTATTTGC |
| X | CGAATCAAGAACCTTGGTG | AAACGTCGCAGATATGGAC |
| XI | GCACCATCCTTTAACTCCAC | GAAAAAGATGCACTCTGTCG |
| XII | TGAGTCGTCATCACCATACG | AGATTTTGTCCAAAGTTAGCAG |
| XIII | GTCATCCTGACATTGCTTTC | ACGATGAGAGAGGAGAAACG |
| XIV | GGTGAAGAAAAGTTTGTAAAATG | TCGACCTCGATCTACTTCG |
| XV | TGTTAAACTACAATTTCGGATTC | CACTGCCATTTTCATGACC |
| XVI | AAGAAGACTTCAAACAATAACGG | GCTAGTAAATTGGTAGCCACC |
