# Supplementary material for: Chimeric Protein Complexes in Hybrid Species Generate Novel Phenotypes
Source: PLoS Genet. 2013 Oct 3;9(10):e1003836. doi: 10.1371/journal.pgen.1003836 (PMC3789821; doi:10.1371/journal.pgen.1003836)
Supplement: Table S16 — List of primers for the specific amplification of the 16 S. mikatae chromosomes. (DOCX) [file pgen.1003836.s047.docx]

| **Chromosome** | **Forward primer sequence 5’-3’** | **Reverse primer sequence 5’-3’** |
| --- | --- | --- |
| I | TGACTAGCCGTTGGATGTAC | CAACGACAGATTTCGAGTACC |
| II | CTAATTAAATCATTAGTGGGGC | GATGGCAATAGATTACCTATGC |
| III | TGATATACGGTACAAGAAAGGG | AAGCTCAAACAAATCCAATG |
| IV | GCAAATAATTCTTATGGCCC | TGGAGGAACAACATTACAAAC |
| V | CTTCTCATAAATAGGCTTGGC | GTTTTACAAAGAAAGCGTGC |
| VI | TGAAATAACTGACTGTCGTTTG | AGGAACATGCCATTCAAAG |
| VII | GCAAAAATCTATAATACTGCTCG | CTACGGATACTGGTGAAATCG |
| VIII | TTGTCCTTTTGTCAGTATGTTG | TTTATCATTTATCTCGTAGGACC |
| IX | CTAGAACATCCTCCGGAATC | GGGTAGACTCCCTAAGTGTTG |
| X | TCCATGGAGCTTAATAGCG | AACTTTGAAGCATCCTTTGAC |
| XI | AGATTGAAGTTCGGATACGTG | TGATCAGCTAGCACATATTGC |
| XII | TTTCACAGTCGTCTCGATTG | CAATTCGGGTTTCCATAAC |
| XIII | CACAGTCATAGGTGAACTGAGG | TTCGGTAAAAACATCCTGG |
| XIV | AAGAGTGCAATGTTACGGG | TAAGTCATGGCAGGTCGG |
| XV | GCTCATTTCTTTACTTGCTTG | ACGTAGAGCTCAAGTAGACGAC |
| XVI | GTCGCCATATAAATGAGATAGC | ACATACAGAGAAGAATACCTTGC |
