# Supplementary material for: Chimeric Protein Complexes in Hybrid Species Generate Novel Phenotypes
Source: PLoS Genet. 2013 Oct 3;9(10):e1003836. doi: 10.1371/journal.pgen.1003836 (PMC3789821; doi:10.1371/journal.pgen.1003836)
Supplement: Table S17 — List of primers for the specific amplification of the 16 S. uvarum chromosomes. (DOCX) [file pgen.1003836.s048.docx]

| **Chromosome** | **Forward primer sequence 5’-3’** | **Reverse primer sequence 5’-3’** |
| --- | --- | --- |
| I | GAACTCAACGTTTTTGGCTG | AACCAGCAGCAGCCTTTG |
| II | GCAGTGTTTTGTATCAGAGGG | CTCGAGTTCAACAGCACTAATAC |
| III | CCGATAAAGTCACACCTCTAGTC | TCAAACCGTAAATAGCTGCG |
| IV | GAATTTGCGTCCTGGTCTC | CGGATGTAAACAAAAACTCTAGC |
| V | AAAGCTGATTTAGCACAATTACC | CGGATAGTGCATTTGATAGG |
| VI | CACCATCAGTCTTACCAGAATAG | GAATCCACATTTGCGATAATTAC |
| VII | CAATGATGAAATGCCACAGTC | TTCCATTTAGAAGAACTAGCGG |
| VIII | CAAGTAAACAAAACAGGTACTTTG | CATATTTGTGTCCCAAGAGATG |
| IX | TTACAACAAAATACCGCCAAG | CGAGCCATATTGGTGCAG |
| X | TTCCATTTTAAAAAACGTTGTG | CCGATCCCTAGTAATGATTCAC |
| XI | CTTAACACGTCCTTATATACCTGG | TGGAGGAACTACAAAGTCAAGTC |
| XII | GGAGAGAACAATCAAGAGAGATG | ATTTTCCAAGTGTAGTCCCATG |
| XIII | GTTCAGAATTGGAAGTGCACAG | CCCAAGGATTTAATATGTTGATG |
| XIV | GGCGTTAGGTAGCTTGCG | ACTTTTGTCGGTGACTTGACC |
| XV | GTAAATTCTTCCTTGCAAATGTG | CTTGCACCTGTTTATCCTGG |
| XVI | CGTAGTTCATTGAAACCTTGG | CAAGTGTAGTGTCCTCAACAGTAG |
