# Supplementary material for: Chimeric Protein Complexes in Hybrid Species Generate Novel Phenotypes
Source: PLoS Genet. 2013 Oct 3;9(10):e1003836. doi: 10.1371/journal.pgen.1003836 (PMC3789821; doi:10.1371/journal.pgen.1003836)
Supplement: Table S18 — List of primers for Real Time PCR. (DOCX) [file pgen.1003836.s049.docx]

| **Primer names** | **Primer sequences 5’-3’** |
| --- | --- |
| Trp2FScer | GGAAAAGGAGATGTCTACCT |
| Trp2RScer | GTGCTGCAGCTTGGTAACCT |
| Trp2FSuv | CACTAACGAGACTTCGCTCG |
| Trp2RSuv | CTTGACCGACGTTTGATTCG |
| Trp3FScer | GAAGGTTACTGCGAGTACCG |
| Trp3RScer | CGAGCATAGATACGGTCCAA |
| Trp3FSuv | CTAACAGTTACTGCCAACAC |
| Trp3RSuv | CGCGTAGATGCGATCCAAAA |
