# Supplementary material for: Chimeric Protein Complexes in Hybrid Species Generate Novel Phenotypes
Source: PLoS Genet. 2013 Oct 3;9(10):e1003836. doi: 10.1371/journal.pgen.1003836 (PMC3789821; doi:10.1371/journal.pgen.1003836)
Supplement: Supplementary file 35 [file pgen.1003836.s027.doc]

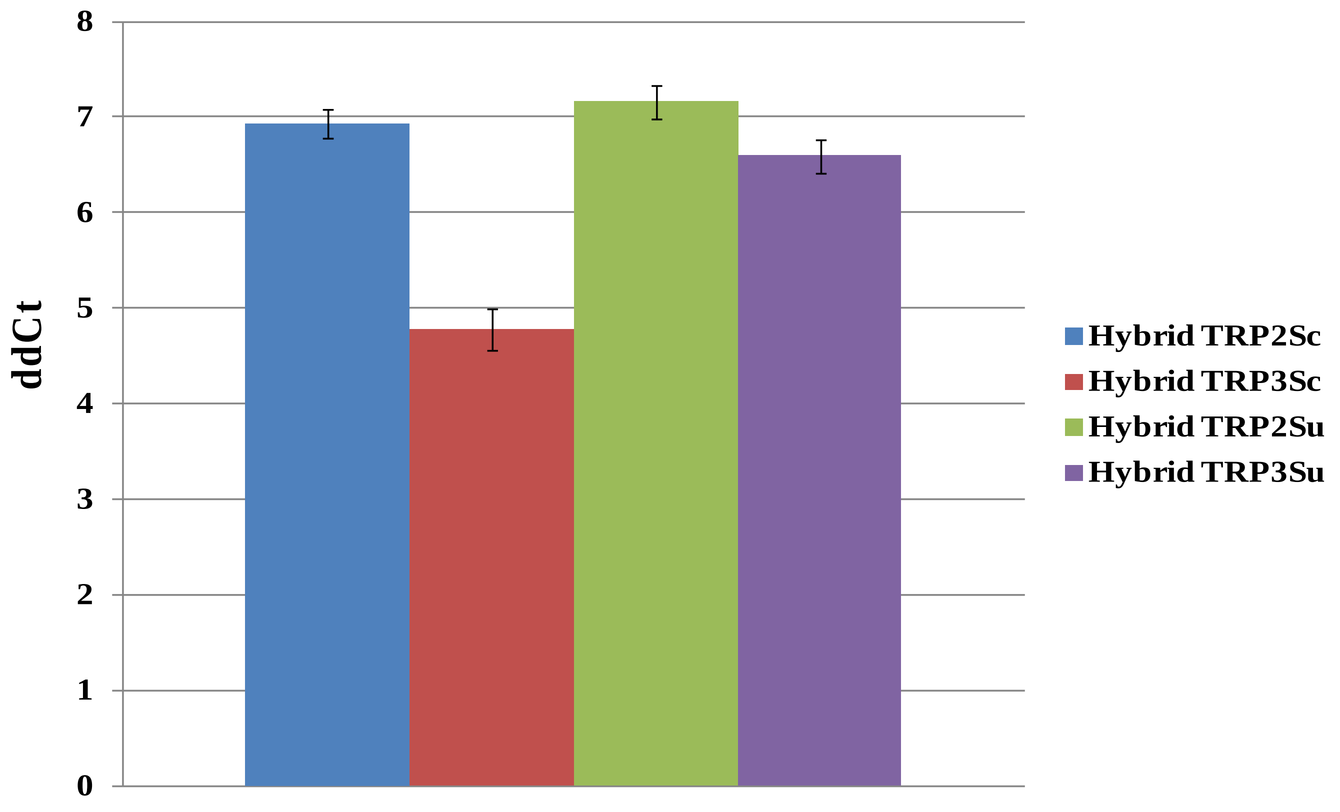

8
7
6
5
4
3
2
1
0
ddCt
Hybrid TRP2Sc
Hybrid TRP3Sc
Hybrid TRP2Su
Hybrid TRP3Su
